# Dioxygen Splitting by a Tantalum(V) Complex Ligated by a Rigid, Redox Non‐Innocent Pincer Ligand**

**DOI:** 10.1002/chem.202203266

**Published:** 2022-11-29

**Authors:** Jack Underhill, Eric S. Yang, Till Schmidt‐Räntsch, William K. Myers, Jose M. Goicoechea, Josh Abbenseth

**Affiliations:** ^1^ Chemistry Research Laboratory Department of Chemistry University of Oxford 12 Mansfield Rd. Oxford OX1 3TA United Kingdom; ^2^ Institut für Anorganische Chemie Georg-August-Universität Göttingen Tammannstraße 4 37077 Göttingen Germany

**Keywords:** oxygen splitting, pincer ligands, radicals, redox chemistry, tantalum

## Abstract

The reaction of TaMe_3_Cl_2_ with the rigid acridane‐derived trisamine H_3_NNN yields the tantalum(V) complex [TaCl_2_(NNN^cat^)]. Subsequent reaction with dioxygen results in the full four‐electron reduction of O_2_ yielding the oxido‐bridged bimetallic complex [{TaCl_2_(NNN^sq^)}_2_O]. This dinuclear complex features an open‐shell ground state due to partial ligand oxidation and was comprehensively characterized by single crystal X‐ray diffraction, LIFDI mass spectrometry, NMR, EPR, IR and UV/VIS/NIR spectroscopy. The mechanism of O_2_ activation was investigated by DFT calculations revealing initial binding of O_2_ to the tantalum(V) center followed by complete O_2_ scission to produce a terminal oxido‐complex.

## Graphical abstract

**The synthesis of a tantalum(V) dichloride complex** ligated by a rigid and redox non‐innocent pincer ligand is reported. Cyclic voltammetry and derivatization reactions show that up to two electrons can be transferred from the ligand to the metal. Exposing this complex to O_2_ leads to the formation of an oxygen bridged dinuclear complex resulting from the full reduction of O_2_ to O^2−^.

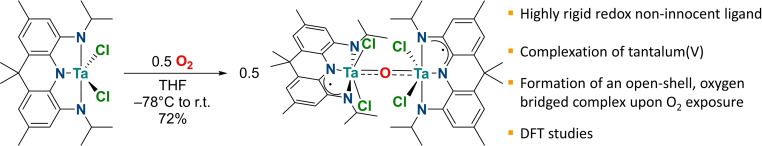

## Introduction

Dioxygen (O_2_) is a green and sustainable oxidant. It plays a key role in the reductive half‐cell reaction of fuel cells, and in biologically‐relevant oxidation reactions mediated by enzymes.^[1]^ However, despite their pivotal role in such processes, molecular complexes with oxido‐ and peroxido‐ligands are typically synthesized by reaction of a metal complex with organic peroxides. This is because the direct reaction of O_2_ with transition metal compounds tends to be low yielding and unselective.[^2^, ^3^] Direct activation of O_2_ has been primarily studied for late and mid‐to‐late transition metals to enable catalytic oxygen atom transfer (OAT) reactions.[^1^, ^4^] Reduction of the O_2_ molecule is usually facilitated by oxidation of the respective transition metal. Consequently, high valent early transition metals, for example zirconium(IV) and tantalum(V), usually cannot reduce O_2_ in the same manner. Nevertheless, it has been shown that d^0^ systems that exhibit alkyl‐alkylidene hydride isomerism can reduce O_2_.^[5]^ The most prominent reaction pathway available to such complexes on reaction with O_2_ is that of radical chain mechanisms, in which O_2_ inserts into metal‐carbon and/or metal‐silicon bonds.[^2^, ^6^]

**Scheme 1:**
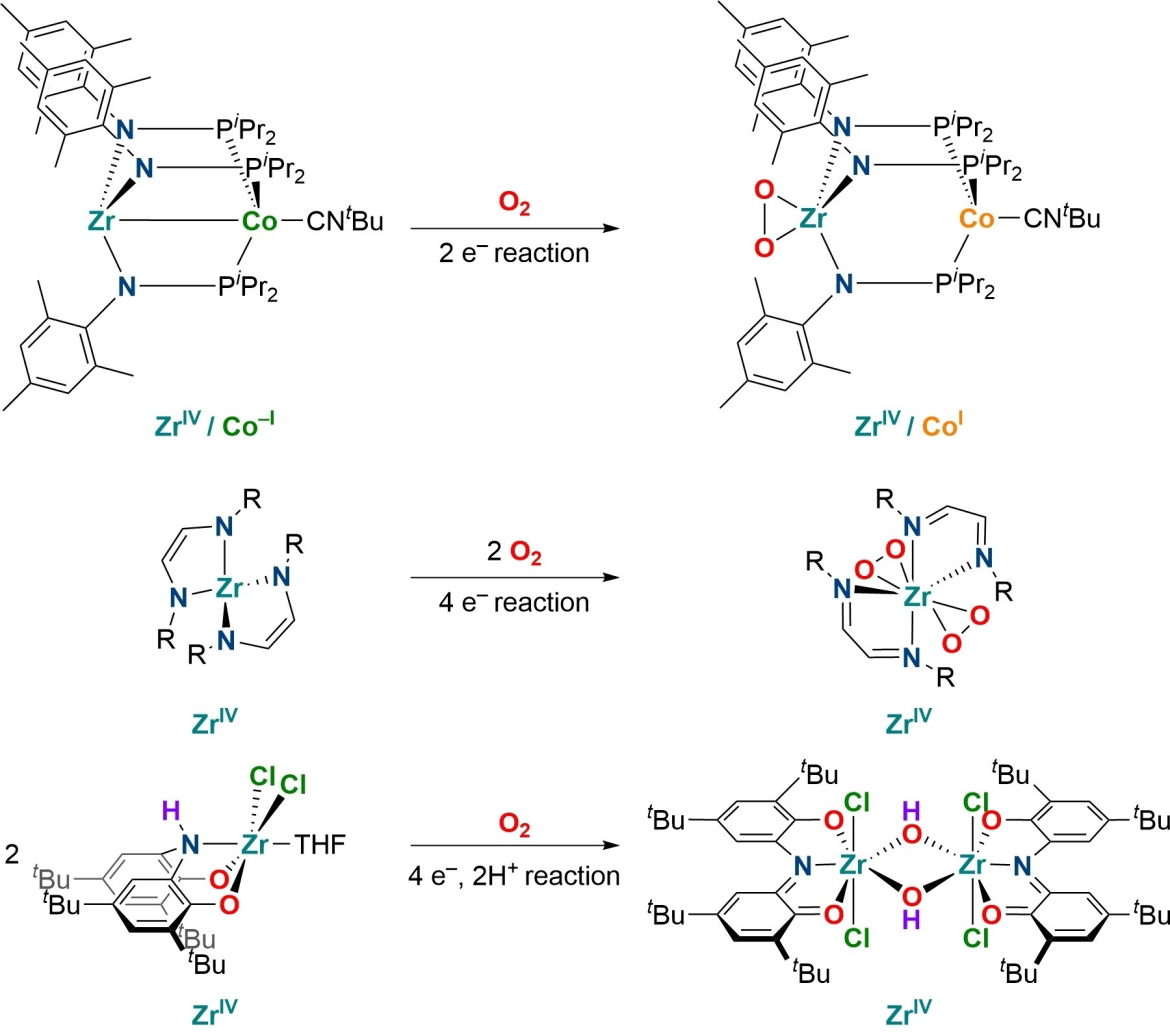
Dioxygen fixation by zirconium(IV) complexes ligated by redox‐active ligands.[^10c^, ^12^, ^13^]

Redox‐active ligands have emerged as powerful supports with which to tune the reactivity of transition metal complexes as they facilitate electron‐transfer to the ligand periphery. Tridentate NNN/ONO pincer ligands are among the most prominent representatives of this class of functional ligands.^[7]^ These redox‐active platforms have been shown to allow for one‐ and two‐electron transformations involving group 3, 4 and 5 metals with a d^0^ configuration. This reactivity has been applied to alkyl‐alkyl cross‐coupling catalysis,^[8]^ nitrene transfer catalysis,^[9]^ and many other processes.^[10]^ However, the application of such systems to small molecule activation reactions, in particular in O_2_ fixation reactions is scarcely explored.^[11]^ O_2_ binding by a Zr/Co hetero‐bimetallic complex was reported to be possible at a formal zirconium(IV) center. This reactivity is facilitated by the synergistic oxidation of the cobalt center of the ligand backbone (Scheme 1, top).^[12]^ Abu‐Omar and co‐workers have demonstrated the reduction of two equivalents of O_2_ to O_2_^2−^ at a zirconium(IV) complex through the participation of redox‐active ligands in an overall 4e^−^ transfer reaction (Scheme 1, middle).^[13]^ Furthermore, it was recently shown that a zirconium(IV) complex ligated by a redox‐active ligand can react with O_2_ to form a dimeric hydroxide bridged complex with the ligand acting as a source of protons and electrons (Scheme 1, bottom).^[10c]^ The full 4 e^−^ reduction of O_2_ towards well defined complexes featuring an O^2−^ ligand remains elusive with these kind of systems.

The use of redox non‐innocent pincer ligands in combination with high valent group 5 elements for dioxygen activation is, to the best of our knowledge, unprecedented, and may offer new strategies for oxidation catalysis by allowing multi‐electron transformation at d^0^ systems reminiscent of mid‐to‐late biomimetic transition metal complexes. Therefore, we set out to investigate the possibility of using a tantalum(V) complex ligated by a redox‐active pincer ligand to study O_2_ reduction.

## Results and Discussion

**Scheme 2:**
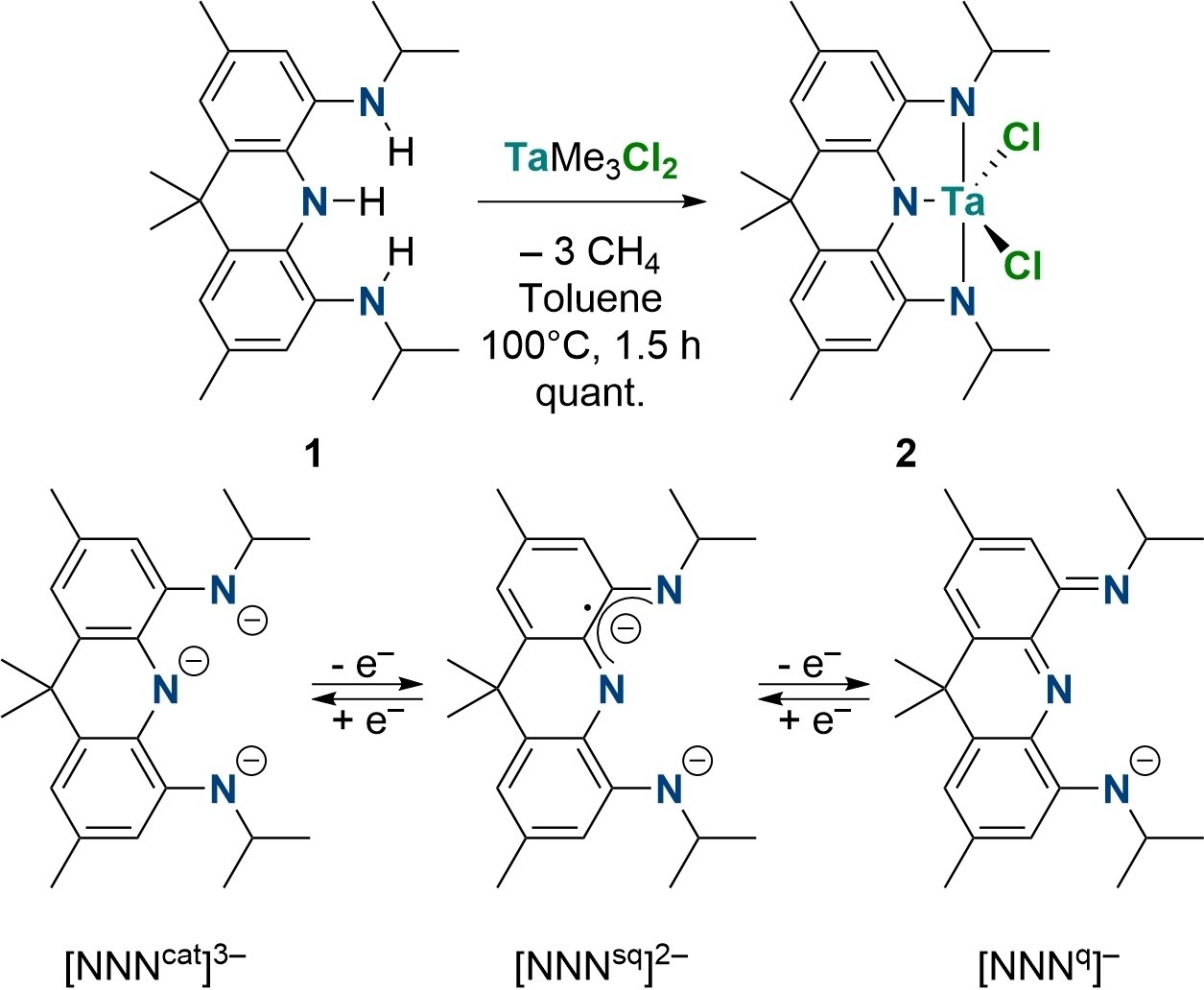
Synthesis of **2** (top), possible oxidation states of the NNN pincer ligand (bottom).

**Figure 1:**
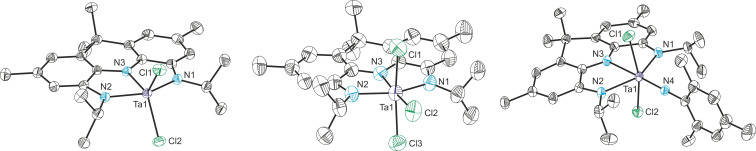
Molecular structures of **2** (left), **3** (middle) and **4** (right) in the solid state as determined by single crystal X‐ray diffraction, hydrogen atoms, counterions and solvent molecules are omitted for clarity.^[14]^ Bond lengths in Å and bond angles in °. **2**: Ta1–N1 2.001(3), Ta–N2 1.992(3), Ta1–N3 2.037(2), Ta1–Cl1 2.3162(8), Ta1–Cl2 2.3113(8); N1–Ta1–N3 72.62(10), N2–Ta1–N3 72.75(10); **3**: Ta1–N1 2.018(3), Ta1–N2 2.034(3), Ta1–N3 2.062(3), Ta1–Cl1 2.4345(10), Ta1–Cl2 2.4737(11), Ta1–Cl3 2.4179(11), N1–Ta1–N2 72.43(11), N2–Ta1–N3 71.98(11); **4**: Ta1–N1 2.151(2), Ta1–N2 2.173(2), Ta1–N3 2.221, Ta1–N4 1.798(2), Ta1–Cl1 2.4201(7), Ta1–Cl2 2.4297(7), N1–Ta1–N3 73.14(8), N1–Ta1–N4 107.09(9), N2–Ta1–N4 107.07(9), N2–Ta1–N3 72.61(8); N3–Ta1–N4 178.12(9).

**Scheme 3:**
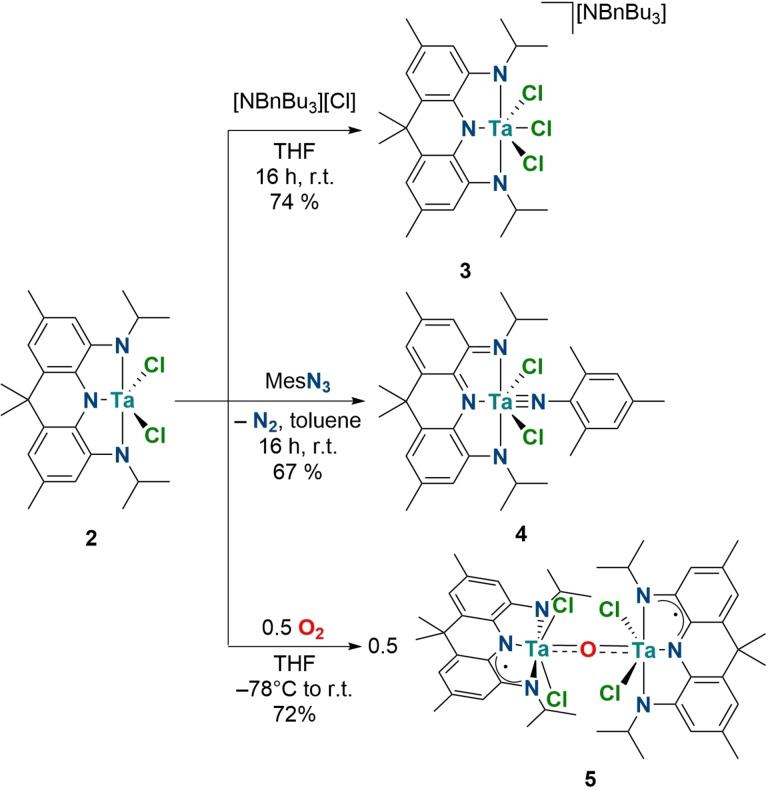
Formation of the anionic trichlorido complex **3** by addition of [NBnBu_3_]Cl to **2** (top); two‐electron oxidation of the NNN pincer upon reaction of **2** with MesN_3_ to produce **4** (middle); four‐electron reduction of O_2_ by **2** to produce **5**. (bottom).

The trisamine **1** was accessed by nitration of 2,7,9,9‐tetramethyl‐9,10‐dihydroacridine with isoamylnitrite, followed by reduction over Pd/C and reductive amination with acetone.^[14]^ **1** displays *C*_2*v*_ symmetry on the NMR timescale and decomposes over the course of several hours when solutions are exposed to oxygen. Heating a mixture of **1** and TaMe_3_Cl_2_ in toluene to 100 °C for 2 h results in the quantitative formation of the tantalum(V) complex [TaCl_2_(NNN^cat^)] (**2**), accompanied by the release of methane (Scheme 2, top). **2** is diamagnetic (*S*=0) and exhibits *C*_2*v*_ symmetry on the NMR timescale. On coordination, the resonance for the isopropyl methine protons shifts to significantly lower fields (Δδ=1.24 ppm), indicative of coordination of the nitrogen donors to tantalum. The proposed structure was confirmed by single crystal X‐ray diffraction (Figure 1), revealing a trigonal bipyramidal coordination mode around the tantalum center. The Ta−N bond distance to the central nitrogen of the pincer ligand is slightly longer (Ta1‐N3: 2.037(2) Å) when compared to the flanking donors (Ta1‐N1: 2.001(3) and Ta1‐N2: 1.992(3) Å). **2** exhibits a dark red color in toluene solutions stemming from multiple low intensity absorptions in the visible range up to 450 nm attributed to ligand‐to‐metal charge transfer excitations, which was corroborated by time‐dependent DFT calculations (TDDFT). DFT analysis of **2** further supports the tantalum(V) oxidation state with no indication of significant ground state electron transfer of the ligand to the metal center, rendering it to be in its fully reduced, trianionic state (Scheme 2, bottom). The highest occupied frontier molecular orbitals (HOMOs) are ligand centered, whereas the lowest unoccupied molecular orbital (LUMO) is metal based.^[14]^ While **2** is stable at elevated temperatures, exposure to wet solvents or air leads to immediate decomposition. Slow decay was also observed when samples were exposed to a dynamic vacuum for extended periods of time, precluding the obtainment of satisfactory combustion analysis data. To investigate the possible redox non‐innocence of the ligand framework, the more stable anionic trichloride complex [NBnBu_3_][TaCl_3_(NNN^cat^)] (**3**) was synthesized by addition of [NBnBu_3_]Cl to **2** in THF (isolated yield of 74 %; Scheme 3). **3** also exhibits *C*_2*v*_ symmetry on the NMR timescale suggesting the formation of an octahedral tantalum trichloro complex, which was confirmed by single crystal X‐ray diffraction. The increase of the coordination number results in an elongation of the Ta−N and Ta−Cl bonds when compared to five‐coordinate **2** (Figure 1). The cyclic voltammogram of **3** in MeCN displays two oxidative redox events at *E*_ox1,1/2_=−0.03 V and *E*_ox2_≈0.38 V (referenced relative to Fc^0/+^). This is in line with recent studies by Heyduk and co‐workers on tantalum pincer complexes with an unfused ligand backbone.^[15]^ While the first oxidation is fully reversible, the second redox event shows quasi‐reversibility (maximum scan rate: 1 V/s).^[14]^

Two‐electron oxidation of the acridane ligand was achieved by addition of MesN_3_ (Mes=2,4,6‐Me_3_−C_6_H_2_) to a freshly prepared toluene solution of **2**. Stirring the solution for 16 h at room temperatures leads to the disappearance of the characteristic deep red color of **2** and a dark green suspension is obtained. The ^1^H NMR spectrum of the purified product reveals the formation of a diamagnetic, *C*_2*v*_ symmetric species, accompanied by a significant shift of the isopropyl methine proton resonance towards higher fields (Δδ=0.24 ppm). Single crystal X‐ray diffraction confirms that [Ta(NMes)Cl_2_(NNN^q^)] (**4**) could be successfully prepared (67 % isolated yield, Figure 1; Scheme 3). Significantly shortened C−N bond distances are obtained and the aromatic ring system of the ligand shows more pronounced alternating single and double bond character of the C−C bonds suggesting a ligand centered oxidation event.[^10f^, ^15^] The imido ligand exhibits a linearly coordinated NMes fragment indicative of a 6 e^−^ donor interaction between the ligand and the tantalum(V) center.[^16^, ^17^] The tantalum imido bond length (Ta1‐N4: 1.798(2) Å) compares well to prior reported examples of octahedral tantalum(V) imido complexes ligated by oxidized redox‐active pincer ligands, lying in between the expected bond lengths, derived from covalent radii, for Ta−N double (1.86 Å) and triple bonds (1.73 Å), respectively.[^10f^, ^15^, ^18^] The UV/VIS spectrum shows a broad transition at *λ*=720 nm which was identified as a ligand centered π−π transition by TDDFT calculations, reflecting the oxidation of the aromatic pincer ligand.^[14]^ Having established the possibility of oxidizing the NNN pincer ligand backbone by up to two electrons when coordinated to a tantalum(V) center, we were interested to see if this reactivity could be applied to the activation of oxygen, in line with the aforementioned studies on zirconium(IV) complexes ligated by redox‐active ligands.

**Figure 2:**
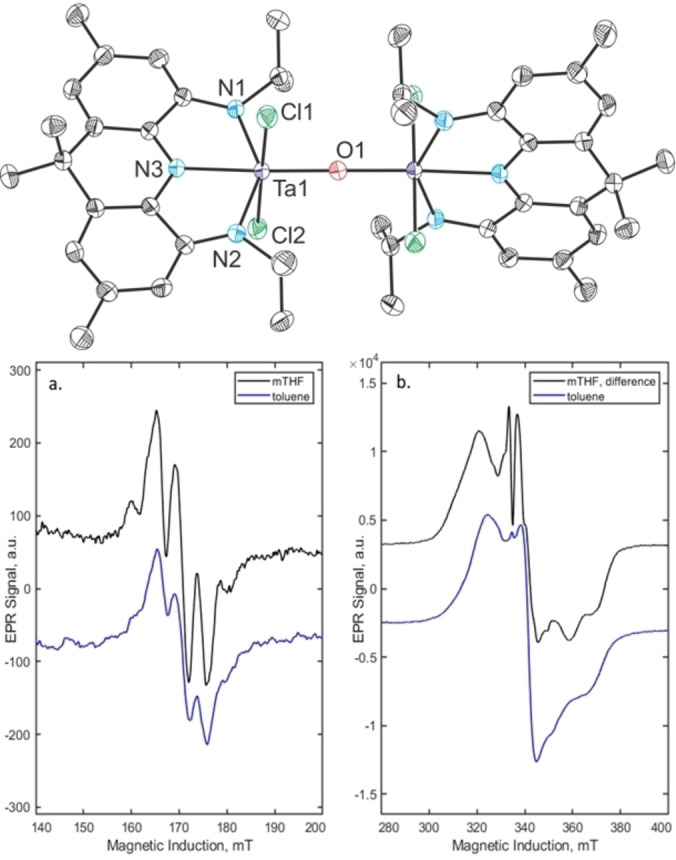
Molecular structure of **5** (top) in the solid state determined by single crystal X‐ray crystallography, hydrogen atoms and solvent molecules were omitted for clarity. Bond lengths in Å and bond angles in °. **5**: Ta1–O1 1.934(1), Ta1–N1 2.042(2), Ta1–N2 2.043(2), Ta1–N3 2.161(2), Ta1–Cl1 2.39(1), Ta1–Cl2 2.420(1), Ta1–O1–Ta1 177.61(14), N1–Ta1–N3 71.69(8), N2–Ta1–N3 71.32(8); CW‐EPR of frozen solutions from dried crystals of **5** collected at 90 K, at X‐band with a 5 mM concentration (bottom). Panel a. shows the half‐field region of Δ$\tilde{M}=\pm 2$ , whilst panel b. has the allowed Δ$\tilde{M}=\pm 1\;$ transitions. Two solvents were used, 2‐methyl‐THF (mTHF, black) and toluene (blue). Spectral subtraction with a 0.5 mM sample was used in the mTHF difference spectrum.^[14]^

When a THF solution of **2** is exposed to an oxygen atmosphere at −78 °C and gradually warmed to room temperature, a color change from dark red to purple and finally brown is observed. This is accompanied by the disappearance of all the NMR signals ascribed to **2**. Instead, multiple broadened resonances can be observed in the ^1^H NMR spectrum suggesting the presence of a paramagnetic product.^[14]^ Prolonged stirring under an oxygen atmosphere at room temperature leads to decomposition and precipitation of a black solid that is insoluble in common organic solvents. Slow diffusion of pentane into a concentrated THF solution of the reaction mixture affords large crystals of the complex [{TaCl_2_(NNN^sq^)}_2_O] (**5**) in 72 % isolated yield (Scheme 3).^[19]^ The presence of an O^2−^ ligand indicates that exposure of **2** to dioxygen leads to the complete scission of O_2_ during the reaction, which represents a 4‐electron reductive process. The complex crystallizes in the orthorhombic space group *I*2/*a* with half a molecule in the asymmetric unit. The two tantalum fragments are bridged by a linearly coordinated oxygen ligand (Ta1‐O1: 1.934(1) Å; Ta1‐O1‐Ta1′: 177.61(14)°) and rotated by ca. 90° with respect to one another (Figure 2). The C−N and C−C bond lengths of the NNN pincer ligand lie in between those measured for **3** and **4**, supporting the presence of a singly oxidized pincer ligand. An intense absorption in the IR spectrum at *ν*=706 cm^−1^ is attributed to an asymmetric Ta−O−Ta vibration (calcd. 703 cm^−1^). LIFDI‐MS measurements of the dissolved crystalline material in toluene show only the expected peak for **5** (M=1216.0 Da). The magnetic moment of **5** was determined by the Evans NMR method to be *μ*_eff_=2.4±0.1 *μ*_B_ in agreement with the presence of two weakly coupled unpaired electrons (spin‐only value: *μ*_S.O._=$\sqrt{6}$ ≈2.45 *μ*_B_). EPR measurements in toluene at room temperature show a single resonance at *g*=1.965, with no detectable hyperfine splitting, suggesting that **5** is a ligand centered diradical, contrasting with previously reported open‐shell NNN Ta trichlorides which featured significant ^181^Ta hyperfine interactions.[^10^, ^15^] Upon cooling a broadened EPR signal was detected as shown in Figure 2, with a half‐field signal that could be detected in the range of 160–185 mT, confirming the presence of two unpaired electrons.

**Figure 3:**
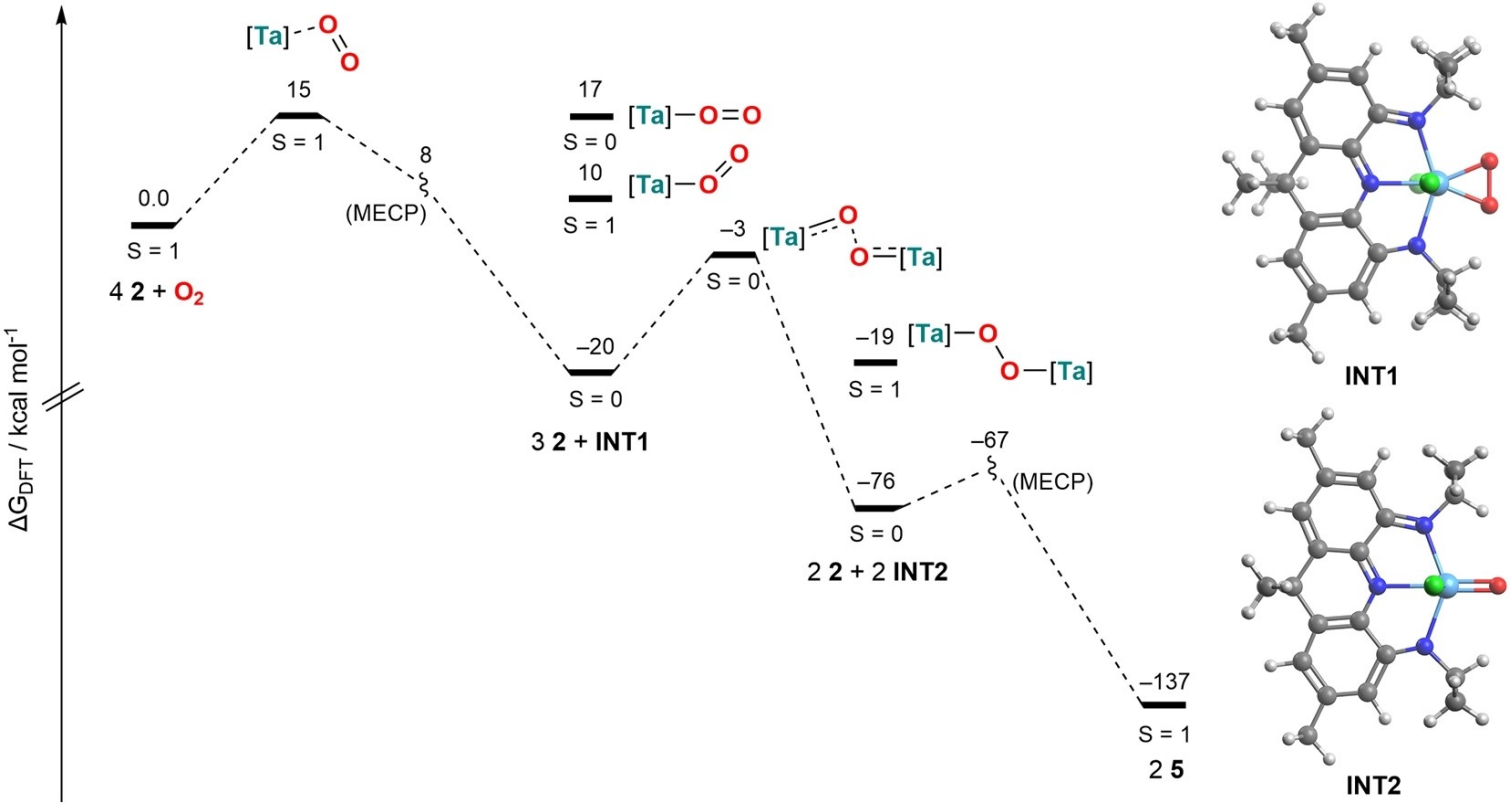
Computationally evaluated mechanism of dioxygen splitting mediated by **2** to produce **5** (left, [Ta]=[TaCl_2_(NNN^x^)] using the ZORA‐ωB97X−D3/{ZORA‐def2‐TZVPP, SARC‐ZORA‐TZVPP(Ta)} method; Molecular structures of computed intermediates **INT1** (top right) and **INT2** (bottom right).

Solutions of **5** are deep purple with *λ*=573 nm. Additionally a broad absorption in the NIR region at *λ*=1098 nm, attributable to ligand centered π−π transitions, again confirms the presence of unpaired electrons on the ligand scaffolds.^[20]^ Theoretical calculations further confirm the experimentally observed open‐shell ground state. While a broken‐symmetry singlet and a triplet state are energetically degenerate (*J*=1.6 cm^−1^), a closed‐shell singlet configuration lies significantly higher in energy. The calculated spin density displays localization of the unpaired electrons on the NNN pincer ligands (92 %) while only minor spin density was found on the tantalum centers (8 %) and none on the bridging oxygen atom. This is in accordance with the localization of the SOMOs (singly occupied molecular orbitals) on the ligand backbone.^[14]^ The operating mechanism for the full reduction of dioxygen was further investigated by DFT calculations (Figure 3). The coordination of O_2_ to **2** is associated with a modest barrier of Δ*G* ^≠^_DFT_=15 kcal/mol on the triplet potential energy surface, followed by a MECP (minimum energy crossing point)^[21]^ towards the *S*=0 surface (Δ*G* ^≠^_DFT_=8 kcal/mol), producing the seven‐coordinate tantalum(V) peroxido‐complex [Ta(*η* ^2^‐O_2_)Cl_2_(NNN^q^)] (**INT1**, Δ*G*_DFT_=−20 kcal/mol, Figure 3), which features a two‐electron oxidized ligand. Terminally bound linear and bent O_2_ adducts were found to be associated with significantly higher ground state energies. A subsequent electrophilic attack by **2** towards **INT1** affords a transition state in which the peroxido coordination mode is disturbed and oxygen atom transfer from **INT1** to **2** takes place (Δ*G* ^≠^_DFT_=−3 kcal/mol) akin to rhenium(V) mediated O_2_ homolysis reported by Sherril and Soper.^[11]^ This results in the formation of two equivalents of the tantalum(V) oxido complex [Ta(O)Cl_2_(NNN^q^)] (**INT2**, Δ*G*_DFT_=−76 kcal/mol). The NNN pincer ligand shows again more pronounced differences of the C−C and C−N bond lengths within the ligand framework, attributable to ligand oxidation. The formation of dinuclear complexes with a bridging O_2_^2−^ ligand was predicted to be thermodynamically unfavorable compared to O_2_ scission on the *S*=0 and *S*=1 potential energy surface.^[14]^ The barrierless coordination of **INT2** towards another equivalent of **2** finally yields the observed dinuclear complex **5** (Δ*G*_DFT_=−137 kcal/mol) in the *S*=1 state. The corresponding MECP was calculated to be thermally accessible with Δ*G* ^≠^_DFT_=−67 kcal/mol. Since no reaction intermediates could be identified by ^1^H NMR spectroscopy the general accessibility of **INT2** was probed by reaction of **2** with 0.5 equivalents of the oxygen atom transfer reagent pyridine‐*N*‐oxide which also produced **5**, albeit with lower isolated yields (40 %).

## Conclusion

In summary, we report the synthesis of a new acridane NNN ligand and the subsequent synthesis of the tantalum(V) dichloride complex **2**. Cyclic voltammetry of the anionic trichloride complex **3**, and reaction of **2** with MesN_3_ producing the imido complex **4** show that oxidation of the pincer ligand by up to two electrons is possible. This reactivity pattern could be used for small molecule activation as shown by O_2_ splitting by **2** to give the oxygen bridged dinuclear complex **5**, which features an open‐shell ground state and represents a rare example of full dioxygen splitting towards O^2−^ mediated solely by redox‐active ligands.

## Experimental Section

For detailed experimental data, including the multi‐step synthesis of **1**, see the electronic Supporting Information. All reactions and product manipulations were carried out under an inert atmosphere of argon or dinitrogen using standard Schlenk or glovebox techniques (MBraun UNILab glovebox maintained at <0.1 ppm O_2_ and <0.1 ppm H_2_O) unless stated otherwise. All solvents that were used for reactions requiring an inert atmosphere were degassed and dried with an MBraun SPS‐800 solvent drying system (SPS) or over Na/K alloy prior to use. NMR spectra were recorded on a Bruker NEO 600 spectrometer with a broadband helium cryoprobe (^1^H: 600 MHz ^13^C: 151 MHz), a Bruker Avance III NMR spectrometer (^1^H: 500 MHz, ^13^C: 126 MHz) and a Bruker Avance III HD nanobay NMR spectrometer (^1^H: 400 MHz, ^13^C: 100 MHz). ^1^H and ^13^C NMR spectra were referenced to the proton resonance of the corresponding solvent (^1^H NMR Tol‐d_8_ δ=2.09 ppm, THF‐d_8_ δ=3.58 ppm; ^13^C NMR Tol‐d_8_ δ=20.4 ppm, THF‐d_8_ δ=67.57 ppm,).

Elemental analysis was performed by: Elemental Analysis Services Team, Science Centre, London Metropolitan University, 29 Hornsey Road, London, N7 7DD, United Kingdom.

Infrared spectra were acquired on a Thermo Scientific iS5 FTIR spectrometer using an iD3 ATR stage and a Bruker ALPHA FTIR spectrometer with a Platinum ATR module.

UV/Vis/NIR spectra were recorded on an Agilent Cary 8454 or Varian Cary 5000 spectrophotometer using quartz cuvettes and air tight caps. All UV/Vis samples were prepared in a glovebox and transferred out of the glovebox prior to the measurement.

LIFDI‐MS measurements were performed by the Zentrale Massenabteilung, Fakultät für Chemie, Georg‐August‐Universität, Göttingen (JEOL AccuTOF JMS‐T100GCV; inert conditions).

Cyclic voltammetry was performed in a MBraun UNILab glovebox with a PalmSens Emstat3+ Blue potentiostat using a glassy carbon working electrode, a Pt wire as counter electrode and an Ag wire as pseudo‐reference electrode.

Continuous wave electron paramagnetic resonance (CW‐EPR) was collected in the Centre for Advanced ESR of the Department of Chemistry of the University of Oxford. The spectrometer was an EMXmicro Premium with a SHQE−W1 resonator. Temperature was controlled with an Oxford Instruments ITC‐503S for an ESR900 cryostat and a Mercury ITC for a CF935O cryostat with liquid N_2_ and liquid helium cryogens. For data presented the microwave frequency was 9.3900(5) GHz, with a microwave power of 633 μW and a field modulation of 0.3 mT amplitude.

Single‐crystal X‐ray diffraction data were collected using an Oxford Diffraction Supernova dual‐source diffractometer equipped with a 135 mm Atlas CCD area detector. Crystals were selected under Paratone‐N oil, mounted on micromount loops and quench‐cooled using an Oxford Cryosystems open flow N_2_ cooling device. Data were collected at 150 K using mirror monochromated Cu Kα (*λ*=1.54184 Å) radiation and processed using the CrysAlisPro package, including unit cell parameter refinement and inter‐frame scaling (which was carried out using SCALE3 ABSPACK within CrysAlisPro).^[22]^ Structures were subsequently solved using direct methods.^[23]^

Density functional theory (DFT) calculations were performed using the ORCA 5.0.2 software package.^[24]^ All methods were used as implemented. Geometries were optimized using the B97‐D3 functional, corrected for relativistic effects using the zeroth order regular approximation (ZORA), the Resolution of Identity approximation (RIJCOSX), and using the segmented all‐electron relativistically contracted split‐valence basis set SARC‐ZORA‐SVP for tantalum and the relativistically contracted split‐valence basis set ZORA‐def2‐SVP for all other atoms, along with the SARC/J auxiliary basis set.^[25]^ Analytical frequency calculations were carried out to verify all geometries were true minima or saddle points. Single point calculations on all compounds and fragments were performed using the ωB97X‐D3 functional and the Resolution of Identity approximation (RIJCOSX).[^25a^, ^25b^, ^26^] The segmented all‐electron relativistically contracted basis set SARC‐ZORA‐TZVPP was used for Ta, and the relativistically contracted triple‐zeta basis set ZORA‐def2‐TZVPP was used for all other atoms, along with the SARC/J auxiliary basis set.[^25c^, ^25d^] The mechanism for the splitting of dioxygen by **2** to produce **5** was probed by relaxed surface scans on singlet, broken‐symmetry open‐shell singlet, and triplet potential energy surfaces. Transition state geometries were subsequently optimized using an eigenvector following algorithm.^[27]^ Minimum energy crossing points (MECPs) between the singlet and triplet potential energy surfaces were located and optimized following the principles suggested by Harvey *et al*.^[28]^

**Synthesis of 2**: **1** (30.0 mg, 85.3 μmol, 1.00 equiv.) and tantalum trimethyl dichloride (25.3 mg, 85.3 μmol, 1.00 equiv.) were dissolved in deuterated toluene (0.5 ml) before being heated to 100 °C for 2 h. The resulting product was then used for characterization and further reactions due to quick decomposition upon attempted workup. Analytical data: NMR (Tol‐d_8_, RT): ^1^H (600 MHz): δ=6.59 (d, ^*4*^ *J*_HH_=1.06 Hz, 2H, C_1/8_ **H**), 6.36 (d, ^4^ *J*_HH_=1.06 Hz, 2H, C_3/6_ **H**), 4.55 (sep, ^3^ *J*_HH_=6.48 Hz, 2H, C_4/5_NC**H**), 2.33 (s, 6H, C_2/7_(C**H**_3_)_2_), 1.47 (s, 6H, C_9_(C**H**_3_)_2_), 1.44 ppm (d, ^3^ *J*_HH_=6.48 Hz, 12H, C_4/5_NC(CH_3_)_2_); ^13^C{^1^H} (151 MHz): δ=144.5 (s, 2 C, **C**_9a/8a_), 140.2 (s, 2 C, **C**_2/7_), 132.1 (s, 2 C, **C**_4a/10a_), 130.0 (s, 2 C, **C**_4/5_), 119.6 (s, 2 C, **C**_1/8_), 109.5 (s, 2 C, **C**_3/6_), 48.2 (s, 2 C, C_4/5_N**C**H), 35.5 (s, 1 C, **C**_9_), 33.0 (s, 2 C, C_9_(**C**H_3_)_2_), 21.4 (s, 2 C, C_2/7_ **C**H_3_), 17.3 ppm (s, 2 C, C_4/5_NC(**C**H_3_)_2_). NMR (THF‐d_8_, RT): ^1^H (400 MHz): δ=6.52 (d, ^4^ *J*_HH_=1.06 Hz, 2H, C_1/8_ **H**), 6.27 (d, ^4^ *J*_HH_=1.06 Hz, 2H, C_3/6_ **H**), 5.14 (sep, ^3^ *J*_HH_=6.48 Hz, 2H, C_4/5_NC**H**), 2.38 (s, 6H, C_2/7_(C**H**_3_)_2_), 1.49 (d, ^3^ *J*_HH_=6.48 Hz, 12H, C_4/5_NC(CH_3_)_2_), 1.47 ppm (s, 6H, C_9_(C**H**_3_)_2_). NMR (C_6_D_6_, RT): ^1^H (400 MHz): δ = 6.64 (d, ^4^ *J*_HH_=1.06 Hz, 2H, C_1/8_ **H**), 6.38 (d, ^4^ *J*_HH_=1.06 Hz, 2H, C_3/6_ **H**), 4.58 (sep, ^3^ *J*_HH_=6.48 Hz, 2H, C_4/5_NC**H**), 2.30 (s, 6H, C_2/7_(C**H**_3_)_2_), 1.53 (s, 6H, C_9_(C**H**_3_)_2_), 1.42 ppm (d, ^3^ *J*_HH_=6.48 Hz, 12H, C_4/5_NC(CH_3_)_2_). UV/Vis (THF, 50 μM, RT, nm): *λ*_max_=450.

**Synthesis of 3**: **1** (50.0 mg, 142 μmol, 1.00 equiv.) and tantalum trimethyl dichloride (42.2 mg, 142 μmol, 1.00 equiv.) were dissolved in toluene (25 ml) before being heated to 100 °C for 2 h. The solvent was removed in vacuo before the addition of *N*‐benzyl‐*N*,*N*‐dibutyl butan‐1‐aminium chloride (44.4 mg, 142.2 μmol, 1.00 equiv.) and THF (25 ml). The mixture was left to stir overnight. The solvent was removed in vacuo and the solid extracted with DCM (10 ml). The DCM solution was layered with pentane (40 ml) and cooled to −30 °C overnight. The solution was filtered, and the solvent was removed, leaving **3** as a dark red solid (95.0 mg, 104 μmol, 74 %). Analytical data: Anal. Calcd. for C_42_H_64_Cl_3_N_4_Ta (912.3): C, 55.3; H, 7.07; N, 6.14 Found: C, 55.6: H, 7.15; N, 6.13. NMR (THF‐d_8_, RT): ^1^H (600 MHz): δ(ppm) 7.47 (m, 5H, C_12‐16_ **H**) 6.30 (d, ^4^ *J*_HH_=1.18 Hz, 2H, C_1/8_ **H**), 6.00 (d, ^4^ *J*_HH_=1.06 Hz, 2H, C_3/6_ **H**), 5.30 (sep, ^3^ *J*_HH_=5.27 Hz, 2H, C_4/5_NC**H**), 3.15 (m, br, 6H, C_18_ **H**_2_), 2.29 (s, 6H, C_2/7_(C**H**_3_)_2_), 1.79 (m, br, 6H, C_19_ **H**_2_), 1.45 (s, 6H, C_9_(C**H**_3_)_2_), 1.33 (m, br, 6H, C_20_ **H**_2_), 1.36 (d, ^3^ *J*_HH_=6.48 Hz, 12H, C_4/5_NC(CH_3_)_2_), 0.96 (t, ^3^ *J*_HH_=7.42 Hz, 9H, C_21_ **H**_2_); ^13^C{^1^H} (151 MHz): δ=146.8 (s, 2 C, **C**_4a/10a_), 136.1 (s, 2 C, **C**_4/5_), 130.5 (s, 2 C, **C**_2/7_), 129.4 (s, 2 C, **C**_9a/8a_), 132.6 (s, 1 C, **C**_11‐16_), 130.4 (s, 1 C, C_11‐16_), 129.1 (s, 1 C, **C**_11‐16_), 127.8 (s, 1 C, **C**_11‐16_), 116.7 (s, 2 C, **C**_1/8_), 108.9 (s, 2 C, **C**_3/6_), 62.1 (s, 1 C, **C**_17_), 58.1 (s, 3 C, **C**_18_), 48.3 (s, 2 C, C_4/5_N**C**H), 34.7 (s, 1 C, **C**_9_), 33.7 (s, 2 C, C_9_(**C**H_3_)_2_), 24.4 (overlapping with THF peak, 3 C, **C**_19_), 20.8 (s, 2 C, C_2/7_ **C**H_3_), 19.5 (s, 3 C, **C**_20_), 17.8 (s, 2 C, C_4/5_NC(**C**H_3_)_2_), 13.0 ppm (s, 3 C, **C**_21_). UV/Vis (THF, 50 μM, RT, nm): *λ*_max_=450.

**Synthesis of 4**: **1** (30.0 mg, 85.3 μmol, 1.00 equiv.) and tantalum trimethyl dichloride (25.3 mg, 85.3 μmol, 1.00 equiv.) were dissolved in toluene (25 ml) before being heated to 100 °C for 2 h. Mesityl azide was added (13.8 mg, 85.3 μmol, 1.00 equiv.) and the reaction mixture was stirred overnight. The solvent was removed in vacuo, the resulting solid was then washed with pentane (3×10 ml) and with diethyl ether (3×10 ml) before any remaining solvent was removed in vacuo. **4** is obtained as a dark green powder (41.9 mg, 57.1 μmol, 67 % yield). Analytical data: Anal. Calcd. for C_32_H_41_Cl_2_N_4_Ta (733.6): C, 52.4; H, 5.63; N, 7.64 Found: C, 52.5: H, 5.87; N, 7.30. NMR (THF‐d_8_, RT): ^1^H (600 MHz): δ = 7.11 (d, ^4^ *J*_HH_=0.74 Hz, 2H, C_1/8_ **H**), 7.04 (d, ^4^ *J*_HH_=0.74 Hz, 2H, C_3/6_ **H**), 6.83 (s, 2H, TaNC**H**), 4.90 (sep, ^3^ *J*_HH_=6.50 Hz, 2H, C_4/5_NC**H**), 2.74 (s, 6H, TaNCC**H**_3_) 2.42 (s, 6H, C_2/7_(C**H**_3_)_2_), 2.32 (s, 3H, TaNCC**H**_3_), 1.69 (s, 6H, C_9_(C**H**_3_)_2_), 1.55 ppm (d, ^3^ *J*_HH_=6.50 Hz, 12H, C_4/5_NC(C**H**_3_)_2_); ^13^C{^1^H} (151 MHz): δ=161.2 (s, 2 C, **C**_4/5_), 150.9 (s, 2 C, TaN**C**CH_3_), 148.3 (s, 2 C, **C**_2/7_), 140.9 (s, 2 C, **C**_9a/8a_), 139.6 (s, 1 C, TaN**C**), 136.4 (s, 2 C, **C**_4a/5a_), 131.8 (s, 1 C, TaN**C**CH_3_), 127.6 (s, 2 C, TaN**C**H), 124.2 (s, 2 C, **C**_1/8_), 113.7 (s, 2 C, **C**_3/6_), 53.2 (s, 2 C, C_4/5_N**C**H), 38.4 (s, 1 C, **C**_9_), 32.9 (s, 2 C, C_9_(**C**H_3_)_2_), 23.7 (s, 2 C, C_4/5_NC(**C**H_3_)_2_), 22.6 (s, 2 C, C_2/7_ **C**H_3_), 20.2 (s, 2 C, TaNC**C**H_3_), 19.9 ppm (s, 2 C, TaNC**C**H_3_). UV/Vis (THF, 50 μM, RT, nm): *λ*_max_=390, 570, 720, 800, 885. LIFDI‐MS (RT, THF, m/z): 732.0 (M^+^).

**Synthesis of 5**: *Route A*: **1** (30.0 mg, 85.3 μmol, 1.00 equiv.) and TaMe_3_Cl_2_ (25.3 mg, 85.3 μmol, 1.00 equiv.) were dissolved in toluene (25 ml) before being heated to 100 °C for 2 h. The solvent was removed in vacuo before the addition of THF (0.5 ml). The solution was freeze/pumped/thawed twice before exposure to O_2_ at −78 °C. The solution was shaken and warmed to room temperature over 3 minutes. The solution was frozen and excess O_2_ was removed. The THF solution was warmed to RT and layered with pentane (5 ml). After five days, the supernatant was removed and the remaining solid was washed with Et_2_O (2×1 ml) and dried. **5** is obtained as a dark purple powder (37.3 mg, 30.7 μmol, 72 %). *Route B*: **1** (30.0 mg, 85.3 μmol, 1.00 equiv.) and tantalum trimethyl dichloride (25.3 mg, 85.3 μmol, 1.00 equiv.) were dissolved in toluene (0.5 ml) before being heated to 100 °C for 2 h. Pyridine‐*N*‐oxide (2.0 mg, 43 μmol, 1.0 equiv.) was added and the solution was heated to 70 °C for 30 minutes. The compound was left to precipitate out of toluene overnight before the suspension was filtered. The filtrate was washed with pentane (1 ml) and Et_2_O (1 ml) before being dried *in vacuo*. **5** is obtained as a dark purple powder (20.0 mg, 16.4 μmol, 40 %). Analytical data: NMR (THF‐d_8_, RT): ^1^H (400 MHz): δ = 42.2 (br), 13.8 (br), −0.14 (br), −1.16 (br), −41.0 ppm (br). **5** cocrystallizes with multiple equivalents of solvent (see Table S1) that cannot be completely removed without inducing decomposition of the complex. UV/Vis (THF, 50 μM, RT, nm): *λ*_max_=580. IR (ATR, RT, cm^−1^): 706 (Ta−O−Ta asym). NIR (RT, toluene, nm): 1100. LIFDI‐MS (RT, THF, m/z): 1216.0 (M^+^). Evans NMR method (THF‐d_8_, RT, *μ*_B_): *μ*_eff_=2.4±0.1 μ_B_.

Deposition Numbers 2191305 (for **IV**), 2191306 (for **1**), 2191307 (for **2**), 2191308 (for **3**), 2191309 (for **4**), 2191310 (for **5** 2pent), 2191311 (for **5** 3tol) These data are provided free of charge by the joint Cambridge Crystallographic Data Centre and Fachinformationszentrum Karlsruhe Access Structures service.

## Conflict of interest

The authors declare no conflict of interest.

1

## Supporting information

As a service to our authors and readers, this journal provides supporting information supplied by the authors. Such materials are peer reviewed and may be re‐organized for online delivery, but are not copy‐edited or typeset. Technical support issues arising from supporting information (other than missing files) should be addressed to the authors.

Supporting Information

## Data Availability

The data that support the findings of this study are available in the supplementary material of this article.
