# Supplementary material for: Dioxygen Splitting by a Tantalum(V) Complex Ligated by a Rigid, Redox Non‐Innocent Pincer Ligand
Source: Chemistry. 2022 Nov 29;29(5):e202203266. doi: 10.1002/chem.202203266 (PMC10098518; doi:10.1002/chem.202203266)
Supplement: Supplementary file 1 — Supporting Information [file CHEM-29-0-s001.pdf]

Chemistry–A European Journal

Supporting Information

Dioxygen Splitting by a Tantalum(V) Complex Ligated by a Rigid, Redox Non-Innocent Pincer Ligand

Jack Underhill, Eric S. Yang, Till Schmidt-Räntsch, William K. Myers, Jose M. Goicoechea,* and Josh Abbenseth*

Table of Contents

Materials and Methods S2
Experimental procedures S3
    Synthesis of **I** S3
    Synthesis of **II** S3
    Synthesis of **III** S3
    Synthesis of **IV** S4
    Synthesis of **V** S4
    Synthesis of **1** S4
Spectroscopic data S6
EPR spectroscopy S34
Crystallographic details S35
Computational details S38
References S43

Materials and Methods

<u>Solvents:</u> Hexane (Sigma Aldrich, HPLC grade), pentane (Sigma Aldrich, HPLC grade), diethyl ether (Sigma Aldrich), benzene (Sigma Aldrich), toluene (Sigma Aldrich), THF (Sigma Aldrich), methyl-THF (Sigma Aldrich), DCM (Sigma Aldrich), MeOH (Sigma-Aldrich, dry). Deuterated Solvents: C_6D_6 (Sigma Aldrich, 99.6%) was dried over CaH_2, vacuum distilled, and degassed before use. $CDCl_3$ (Sigma Aldrich, 99.6%) was used without further purification. Tol-d_8 (Sigma Aldrich, 99.6%) was dried over Na/K, vacuum distilled, and degassed before use. THF-d_8 (Sigma Aldrich, 99.6%) was dried over Na/K, vacuum distilled, and degassed before use. All dry solvents were stored under argon in gas-tight ampoules over 3 Å molecular sieves.

<u>Reagents purchased from commercial suppliers:</u> $TaCl_5$ (Thermo Fisher), $ZnMe_2$ (Sigma-Aldrich), pyridine-*N*-oxide (Sigma Aldrich), O_2 (Linde), *N*-benzyl-*N*,*N*-dibutyl-butan-1-aminium chloride (Sigma-Aldrich), methyl 2-amino-5-methyl benzoate (Fluorochem), 4-iodotoluene (Sigma-Aldrich), Cu powder (Sigma-Aldrich), K_2CO_3 (Sigma-Aldrich), NaCl (Sigma-Aldrich), MeLi (Sigma-Aldrich), AcOH (Sigma Aldrich), H_3PO_4 (85% in water, Sigma Aldrich), NaOH (Sigma-Aldrich), Na_2SO_4 (TCI), AcOH (Sigma-Aldrich), HNO_3 (70% solution, Sigma-Aldrich), isopentyl nitrite (Sigma-Aldrich), Pd/C (Sigma-Aldrich), H_2 (Sigma-Aldrich).

$TaMe_3Cl_2$ and mesityl azide were prepared according to previously reported literature procedures.[1] Pentane for the synthesis of $TaMe_3Cl_2$ was dried over NaK. *N*-benzyl-*N,N*-dibutyl-butan-1-aminium chloride was recrystallized two times from MeOH prior to use. Pyridine-*N*-oxide was sublimed prior to use. Methyl-THF was dried over sodium, vacuum distilled and degassed before use.

Experimental procedures

The synthesis of compounds **I** – **III** follows the procedure reported by Shi and co-workers and Emslie and co-workers with minor modifications.[2]

Synthesis of I

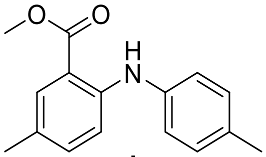

I

Methyl 2-amino-5-methyl benzoate (3.00 g, 18.2 mmol, 1.00 eq.), 4-iodotoluene (3.96 g, 18.2 mmol, 1.00 eq.), Cu powder (175 mg, 2.72 mmol, 0.15 eq.) and K_2CO_3 (2.51 g, 18.2 mmol, 1.00 eq.) were mixed together under an inert atmosphere and heated to 170 °C for 16 h. The product was extracted with chloroform (50.0 ml) and water (50.0 ml) after cooling. The aqueous phase was extracted twice with chloroform (2 × 20.0 ml). The organic phase was filtered to remove the Cu powder, before being washed twice with water (2 × 20.0 ml) and twice with brine (2 × 20.0 ml). The organic phase was dried over Na_2SO_4 before the solvent was removed, leaving a yellow brown oil. The oil was re-dissolved in PET 40/60 (10 ml) and filtered, after which the solvent was removed and the oil left to dry overnight. The obtained yellow oil is filtered over cotton wool to give **I** (4.27 g, 16.72 mmol, 92 %).
Analytical data: ^{1}H NMR spectra matched published spectra.[3]

Synthesis of II

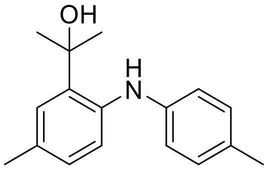

II

I (4.27 g, 16.9 mmol, 1.00 eq.) was dissolved in dry THF and cooled to –78 °C. MeLi (42.3 ml, 67.7 mmol, 4.00 eq.) was added dropwise. This was left to stir for 15 mins at –78 °C. The mixture was warmed to room temperature and stirred for 1 h. Water was slowly added under an argon counter stream and left to stir for 15 minutes. The product was extracted with Et_2O twice (20 mL). The organic phase was washed once with water (20 mL) and brine (20 mL), before being dried over Na_2SO_4, filtered and dried. **II** (4.24 g, 16.7 mmol, 99 %) is obtained as a yellow oil.
Analytical data: ^{1}H NMR spectra matched published spectra.[3]

Synthesis of III

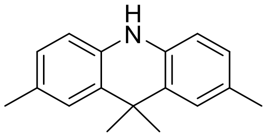

III

The obtained oil from the synthesis of **II** (4.24 g, 16.7 mmol, 1.00 eq.) was suspended in H_3PO_4 (25.0 ml, excess, 85 % in water) and heated to 120 °C for 2.5 h. The mixture was cooled to room temperature, before being diluted with water (20 mL) and extracted with water (50.0 ml) and Et_2O (50.0 ml). After separating the phases, the aqueous phase was neutralized with NaOH (2 M, 3 × 100 ml) in an ice bath and extracted again with Et_2O (50.0 ml). The combined organic phases were washed once with water (50.0 ml) and brine (50.0 ml). After drying over Na_2SO_4, the solvent was removed. The obtained oil was re-dissolved in hexane and the solvent was removed again. The product solidifies upon drying in vacuum affording **III** (2.82 g, 11.88 mmol, 72 %) as a pink/red solid.
Analytical data: ^{1}H NMR spectra matched published spectra.[3]

Synthesis of IV

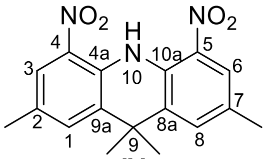

IV

III (1.00 g, 4.21 mmol, 1.00 eq.) was dissolved in 50 ml AcOH. Once fully dissolved, HNO_3 was added (1.75 ml, 70 % solution) followed by the dropwise addition of isopentyl nitrite (1.70 ml, 12.6 mmol, 3.00 eq.) The resulting suspension was stirred for 15 minutes and filtered using a Büchner funnel. After washing of the precipitate with water (40 mL) the product is dried under vacuum. **IV** (1.08 g, 3.31 mmol, 78 %) is obtained as a bright red powder.

Analytical data: NMR ($CDCl_3$, RT): ^{1}H (600 MHz): δ = 12.71 (s, 1H, N_{10}**H**), 7.99 (d, $^4J_{HH}$ = 1.59 Hz, 2H, $C_{3/6}$**H**), 7.49 (d, $^4J_{HH}$ = 1.59Hz, 2H, $C_{1/8}$**H**), 2.39 (s, 6H, $C_{2/7}$C**H$_3$**), 1.62 ppm (s, 6H, C_9(C**H**$_3$)$_2$); ^{13}C{^{1}H} (151 MHz): δ = 133.7 (s, 2C, **C**$_{4/5}$), 133.2 (s, 2C, **C**$_{1/8}$), 132.9 (s, 2C, **C**$_{4a/10a}$), 131.8 (s, 2C, **C**$_{9a/8a}$), 130.8 (s, 2C, **C**$_{2/7}$), 124.6 (s, 2C, **C**$_{3/6}$), 37.2 (s, 1C, **C**$_9$), 31.5 (s, 2C, $C_{2/7}$**C**H$_3$), 21.0 ppm (s, 2C, C_9(**C**H$_3$)$_2$). IR (ATR, RT, cm^{-1}): v = 1500 (NO_2, asym), 1305 (NO_2, sym).

Synthesis of V

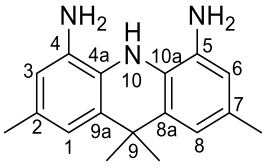

V

IV (50.0 mg, 0.153 mmol, 1.00 eq) and Pd/C (10 w%, 8.1 mg, 7.6 µmol, 0.05 eq.) were suspended in ethyl acetate (10 mL). This suspension was thoroughly degassed and backfilled with H_2 (1.00 atm, excess). The reaction was heated to 50.0 °C for 16 h. After the reaction was complete, filtration, extraction with THF (5 mL) and removal of the solvent afforded crude **V** as a white solid. NMR spectroscopy showed full conversion of the starting material. The high air and moisture sensitivity resulted in direct use in the following step.

Analytical data: NMR (THF-d$_8$, RT): ^{1}H (600 MHz): δ = 6.60 (d, $^4J_{HH}$ = 1.13 Hz, 2H, $C_{1/8}$**H**), 6.36 (d, $^4J_{HH}$ = 1.13 Hz, 2H, $C_{3/6}$**H**), 5.84 (s, 1H, N_{10}**H**), 4.04 (s, 4H, $C_{4/5}$N**H**$_2$), 2.18 (s, 6H, $C_{2/7}$C**H**$_3$), 1.50 ppm (s, 6H, C_9(C**H**$_3$)$_2$); ^{13}C{H} (151 MHz): δ = 134.5 (s, 2C, **C**$_{4/5}$), 129.9 (s, 2C, **C**$_{4a/5a}$), 129.4 (s, 2C, **C**$_{8a/9a}$), 127.0 (s, 2C, **C**$_{2/7}$), 117.2 (s, 2C, **C**$_{1/8}$), 115.7 ppm (s, 2C, **C**$_{3/6}$).

Synthesis of 1

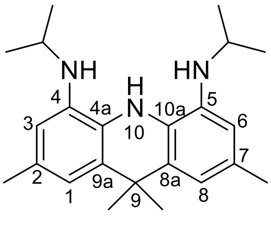

1

IV (1.08 g, 3.30 mmol, 1.00 eq.) and Pd/C (10 w%, 175 mg, 165 µmol, 0.05 eq.) were suspended in ethyl acetate (10 mL). The resulting suspension was thoroughly degassed and backfilled with H_2 (1.00 atm, excess). The reaction was heated to 50 °C for 16 h. On the completion of the reaction, the solvent was removed and the solid was extracted with MeOH (10 mL). Acetone (2.44 ml, 33.0 mmol, 10.0 eq.) and molecular sieves (4 Å) were added. The solution was heated to 60 °C and left to stir for 5 h followed by filtration onto Pd/C (10 w%, 351 mg, 330 µmol, 0.10 eq.). The mixture was degassed and backfilled with hydrogen (2.00 atm, excess) before the suspension was left to stir for 16 h at room temperature. The reaction mixture was filtered, the solvent was removed and **1** (800 mg, 2.28 mmol, 69 %) was lyophilized out of benzene leaving a white solid.

Analytical data: NMR (C_6D_6, RT): ^{1}H (600 MHz): δ = 7.01 (d, $^4J_{HH}$ = 1.16 Hz, 2H, $C_{1/8}$**H**), 6.51 (d, $^4J_{HH}$ = 1.16 Hz, 2H, $C_{3/6}$**H**), 3.34 (sep, $^3J_{HH}$ = 6.58 Hz, 1H, $C_{4/5}$NC**H**), 2.35 (s, 1H, N_{10}**H**), 2.34 (s, 6H, $C_{2/7}$C**H**$_3$), 1.69 (s, 6H, C_9(C**H**$_3$)$_2$), 1.04 ppm (d, $^3J_{HH}$ = 6.58 Hz, 12H, $C_{4/5}$NC(C**H**$_3$)$_2$); ^{13}C{^{1}H} (151 MHz): δ = 133.5 (s, 2C, **C**$_{4/5}$), 130.4 (s, 2C, **C**$_{4a/5a}$),

129.6 (s, 2C, **C**$_{2/7}$), 129.0 (s, 2C, **C**$_{8a/9a}$), 119.6 (s, 2C, **C**$_{1/8}$), 118.0 (s, 2C, **C**$_{3/6}$),46.4 (C$_{4/5}$N**C**H), 36.9 (s, 1C, **C**$_{9}$), 301.2 (s, 2C, C$_{9}$(**C**H$_{3}$)$_{2}$), 23.4 (s, 4C, C$_{4/5}$NC(**C**H$_{3}$)$_{2}$), 21.7 ppm (s, 2C, C$_{2/7}$**C**H$_{3}$).

Table S1. Elemental analysis measurements of **5**.

**$C_{46}H_{60}Cl_4N_6OTa_2$*$(C_5H_{12})_{0.6}$ (1260.0):**		**Calcd**	**Found 1**	**Found 2**
	C	46.7	46.8	46.8
	H	5.38	5.03	5.27
	N	6.67	6.59	6.63
**$C_{46}H_{60}Cl_4N_6OTa_2$*$(C_5H_{12})_{0.8}$ (1274.4):**		**Calcd**	**Found 1**	**Found 2**
	C	47.1	47.1	47.1
	H	5.50	5.30	5.33
	N	6.59	6.61	6.59
**$C_{46}H_{60}Cl_4N_6OTa_2$*$(C_5H_{12})_{0.9}$ (1281.7):**		**Calcd**	**Found 1**	**Found 2**
	C	47.3	47.4	47.0
	H	5.57	5.46	5.42
	N	6.56	6.65	6.64

Spectroscopic data

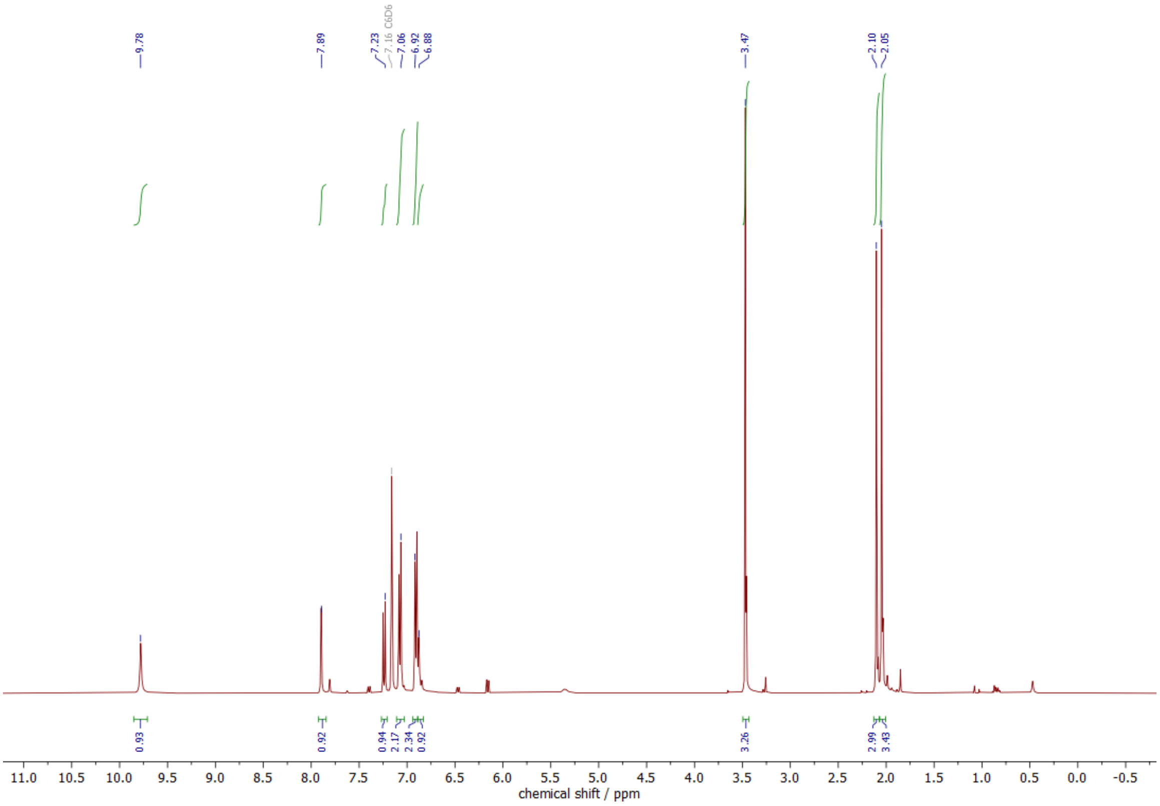

Figure S1. ^{1}H NMR spectrum of **I**, $CDCl_3$, RT (400 MHz).

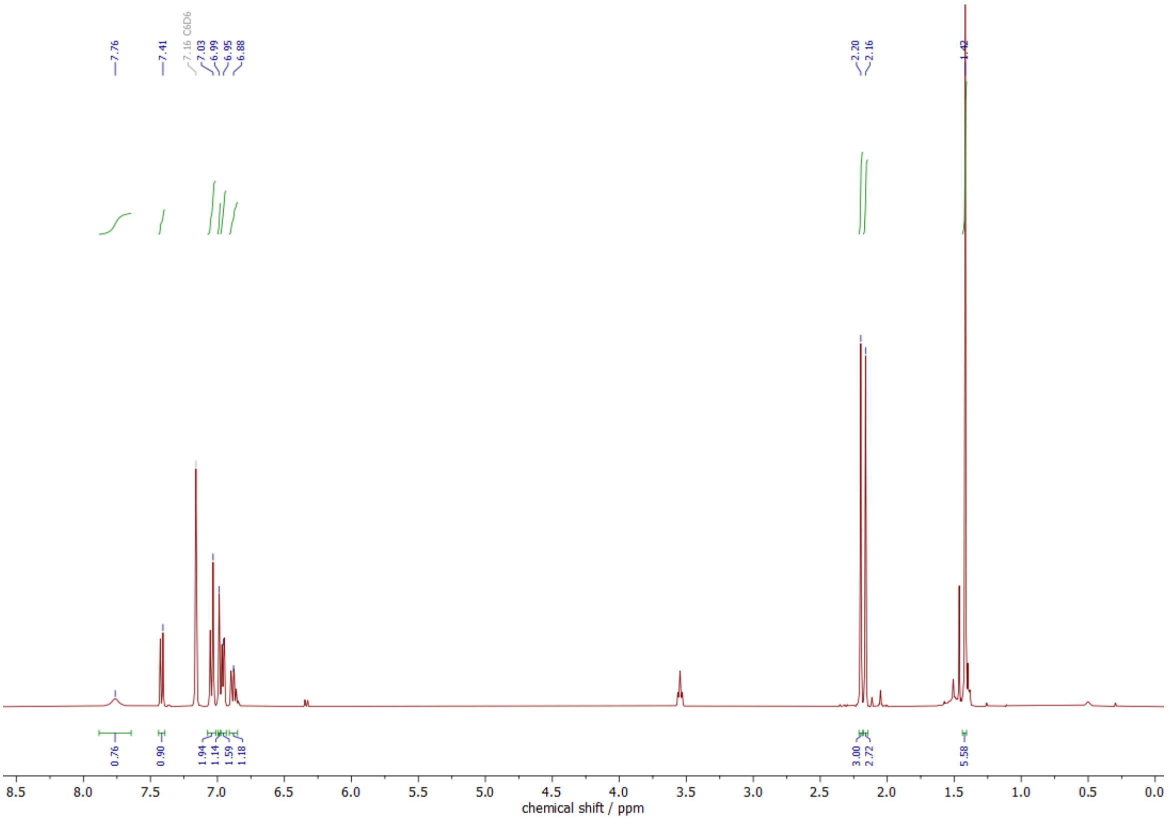

Figure S2. ^{1}H NMR spectrum of **II**, $CDCl_3$, RT (400 MHz).

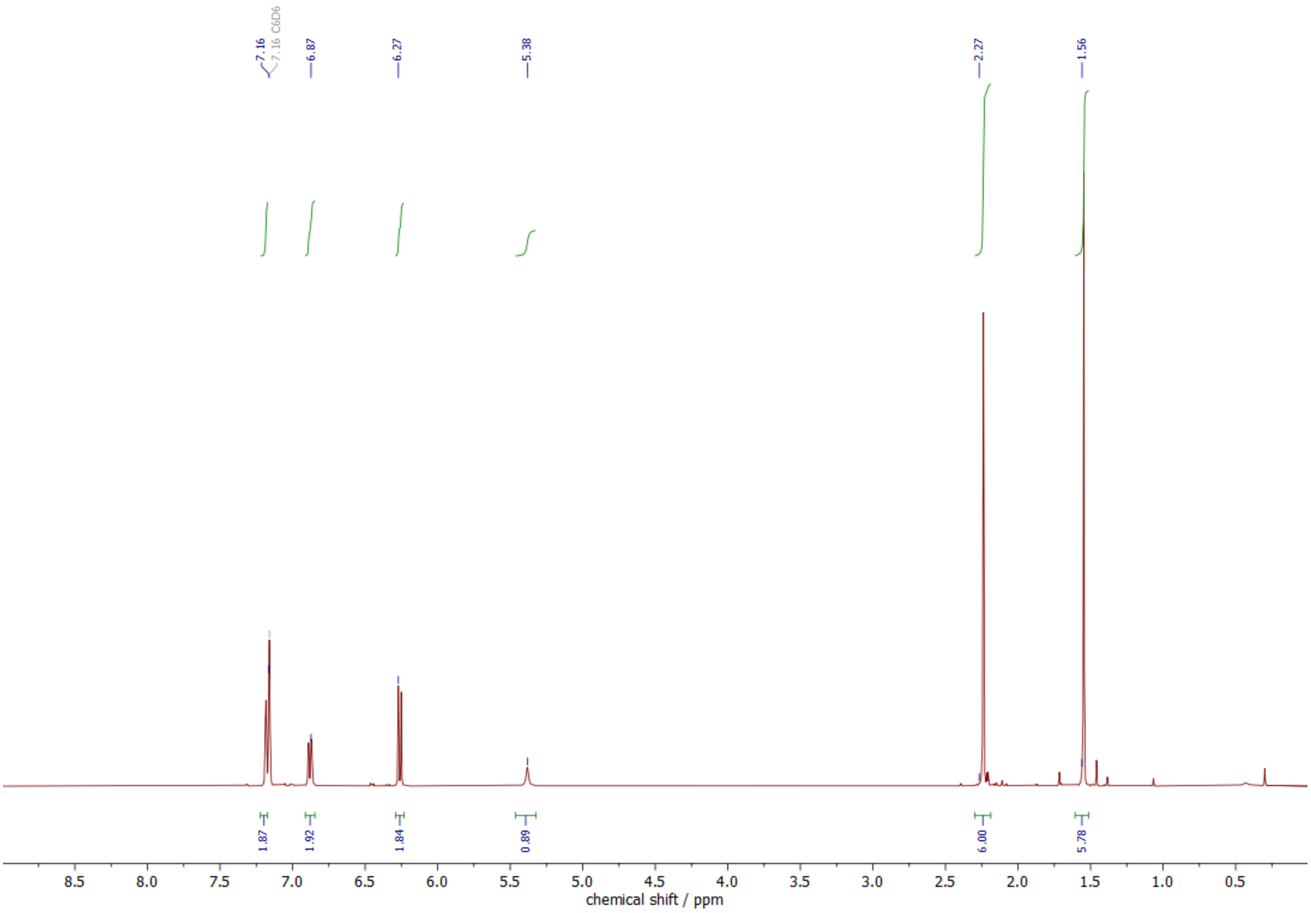

Figure S3. ^{1}H NMR spectrum of **III**, $CDCl_3$, RT (400 MHz).

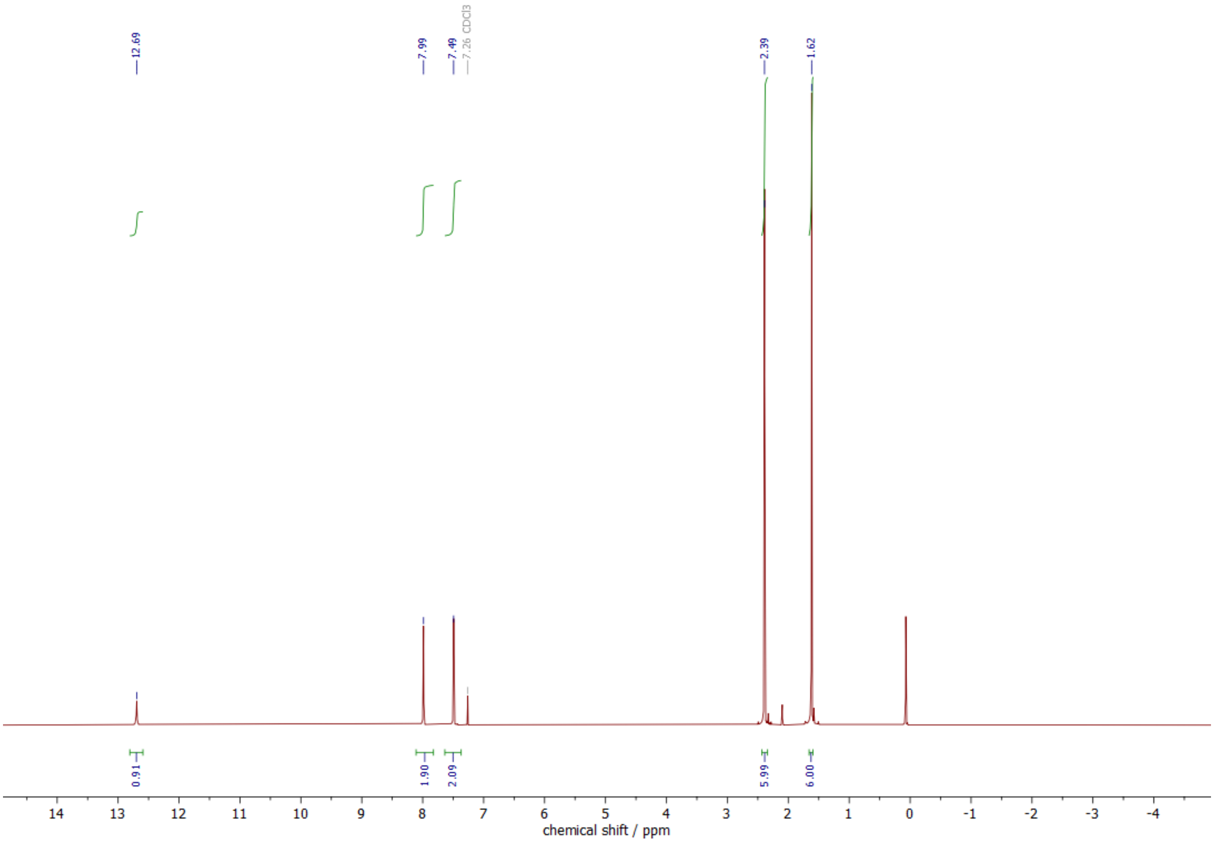

Figure S4. ^{1}H NMR spectrum of **IV**, $CDCl_3$, RT (600 MHz).

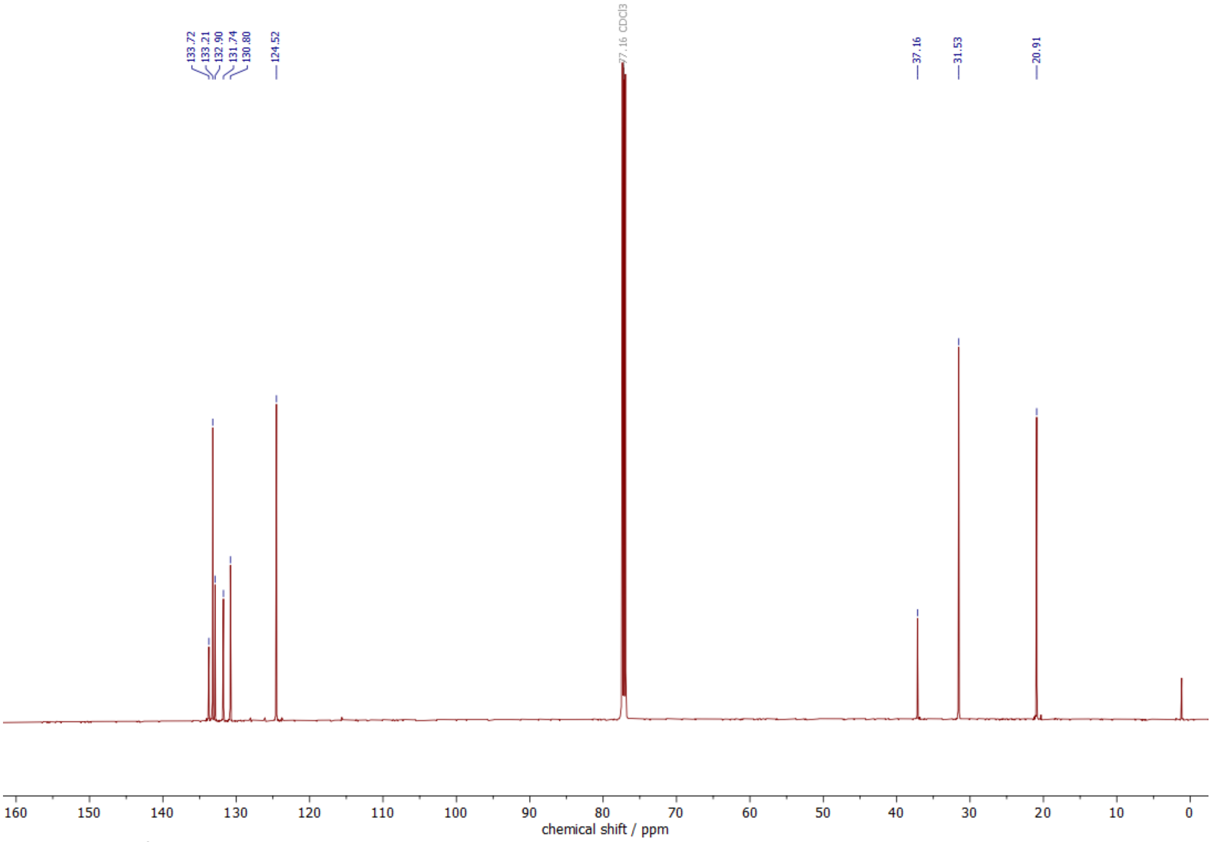

Figure S5. $^{13}C\{^{1}H\}$ NMR spectrum of **IV**, $CDCl_3$, RT (151 MHz).

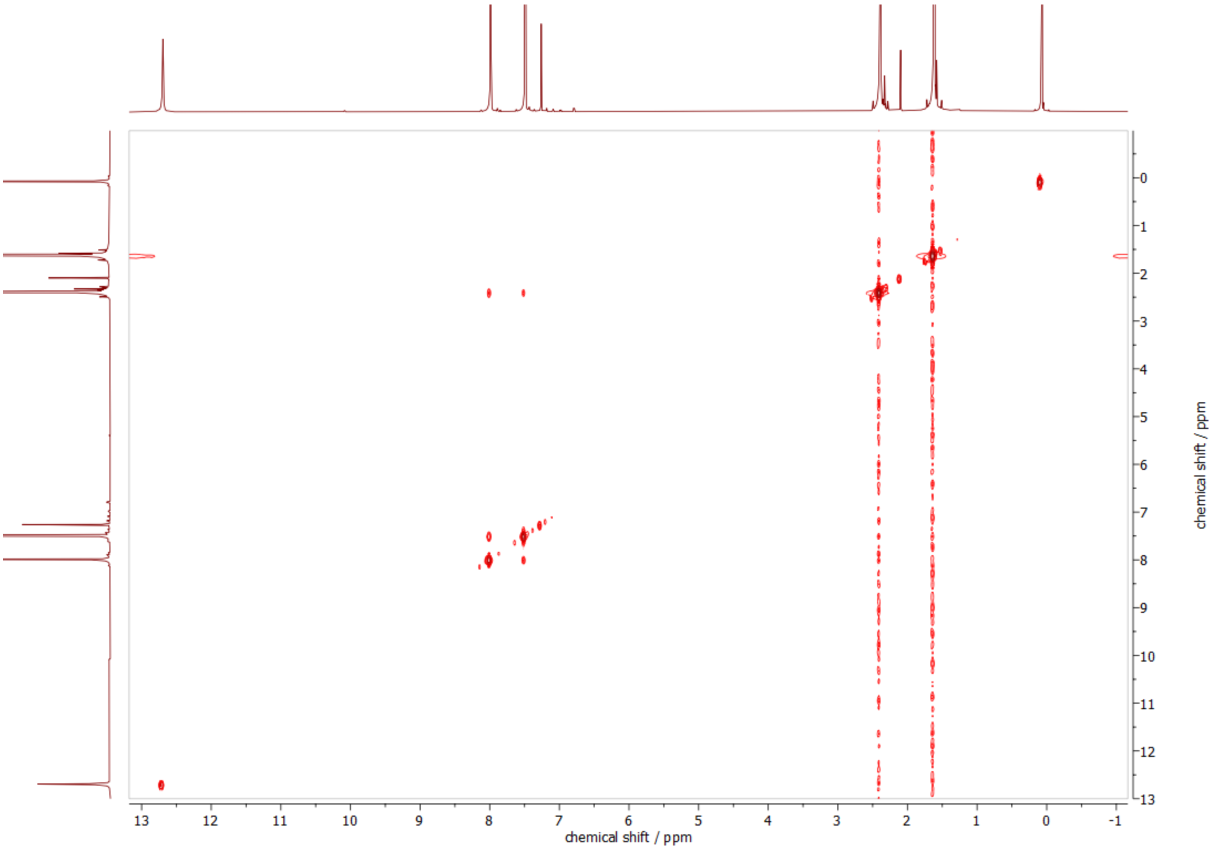

Figure S6. COSY NMR spectrum of **IV**, $CDCl_3$, RT.

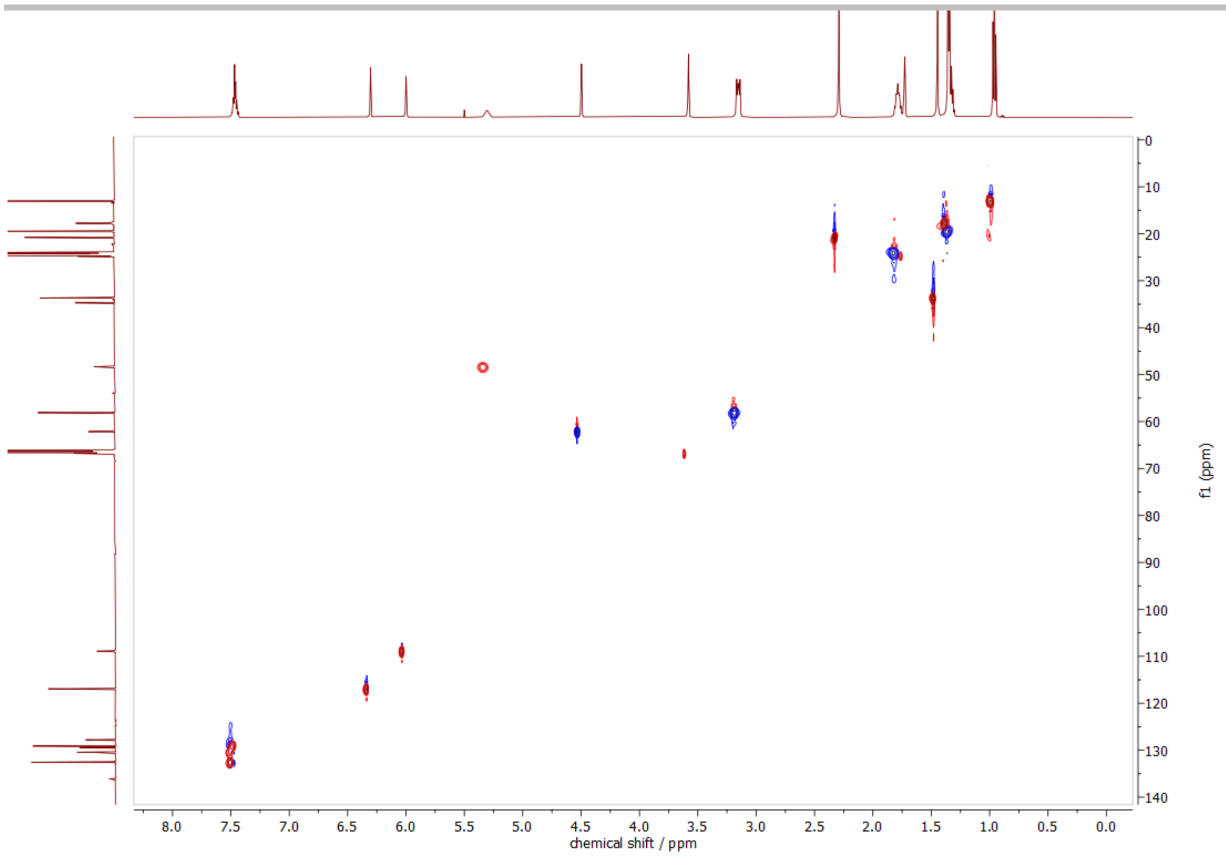

Figure S7. HSQC NMR spectrum of **IV**, $CDCl_3$, RT.

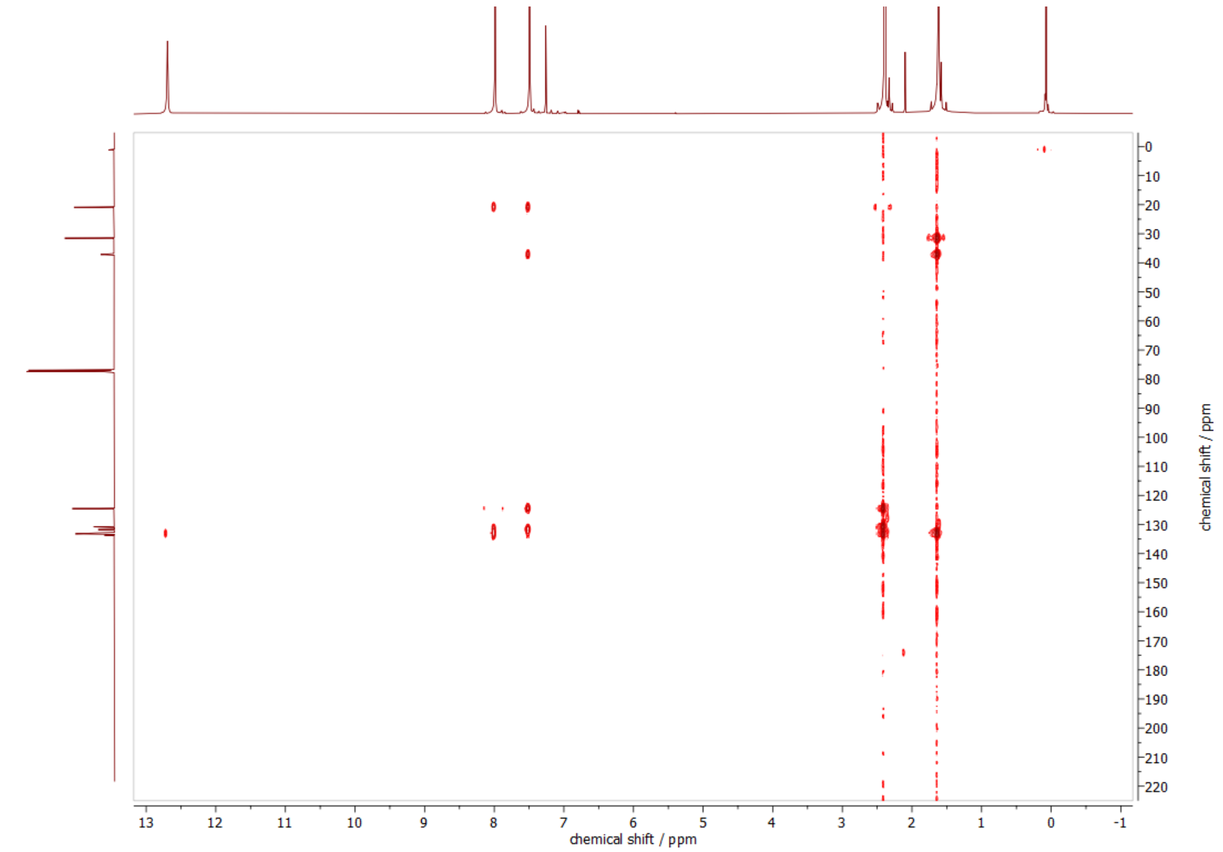

Figure S8. HMBC NMR spectrum of **IV**, $CDCl_3$, RT.

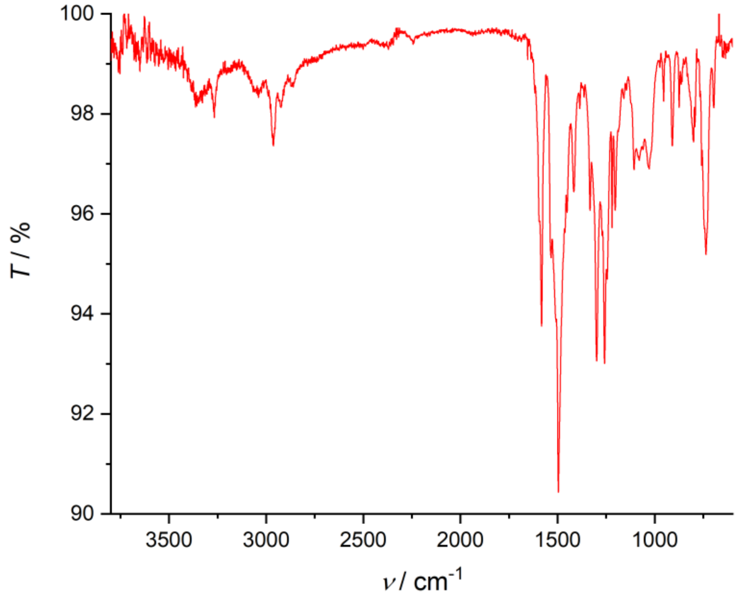

Figure S9. IR spectrum of **IV**, ATR, RT.

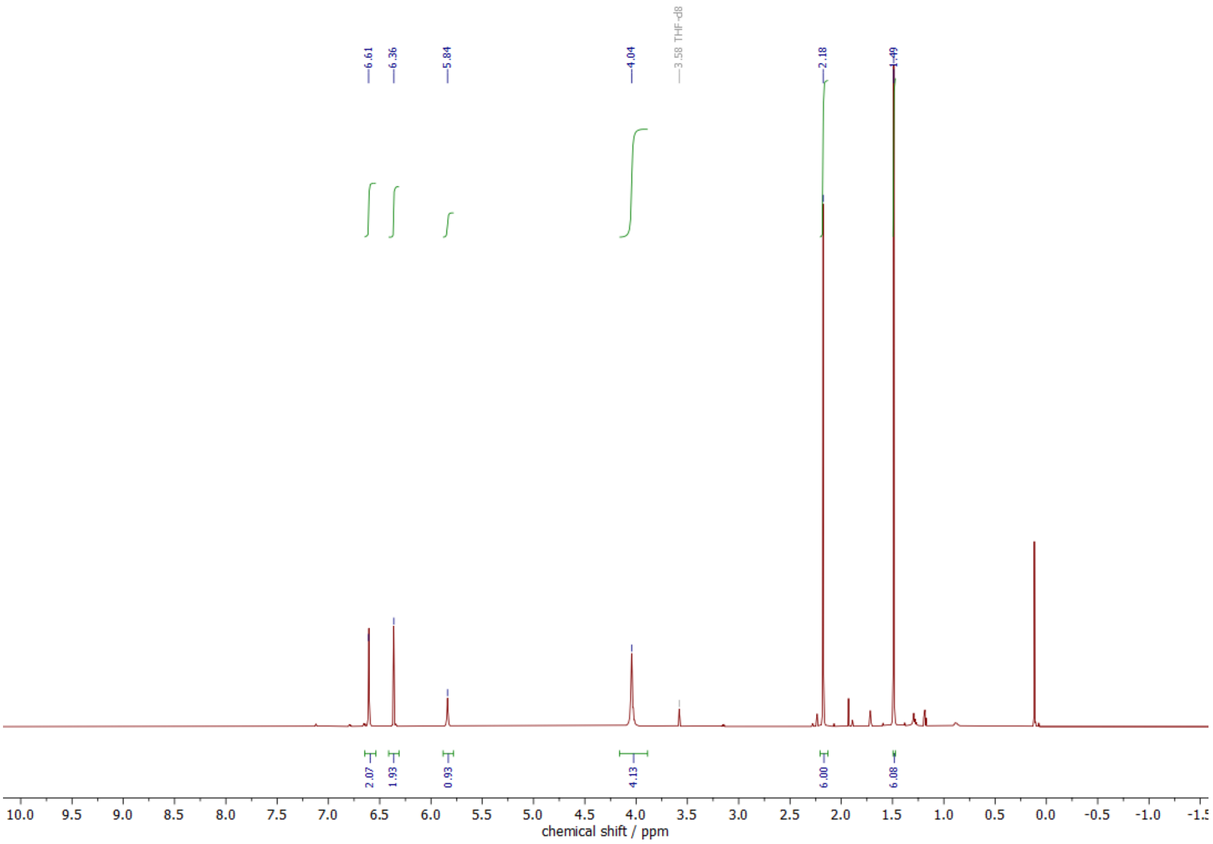

Figure S10. ^{1}H NMR Spectrum of **V**, THF-d_8, RT (600 MHz).

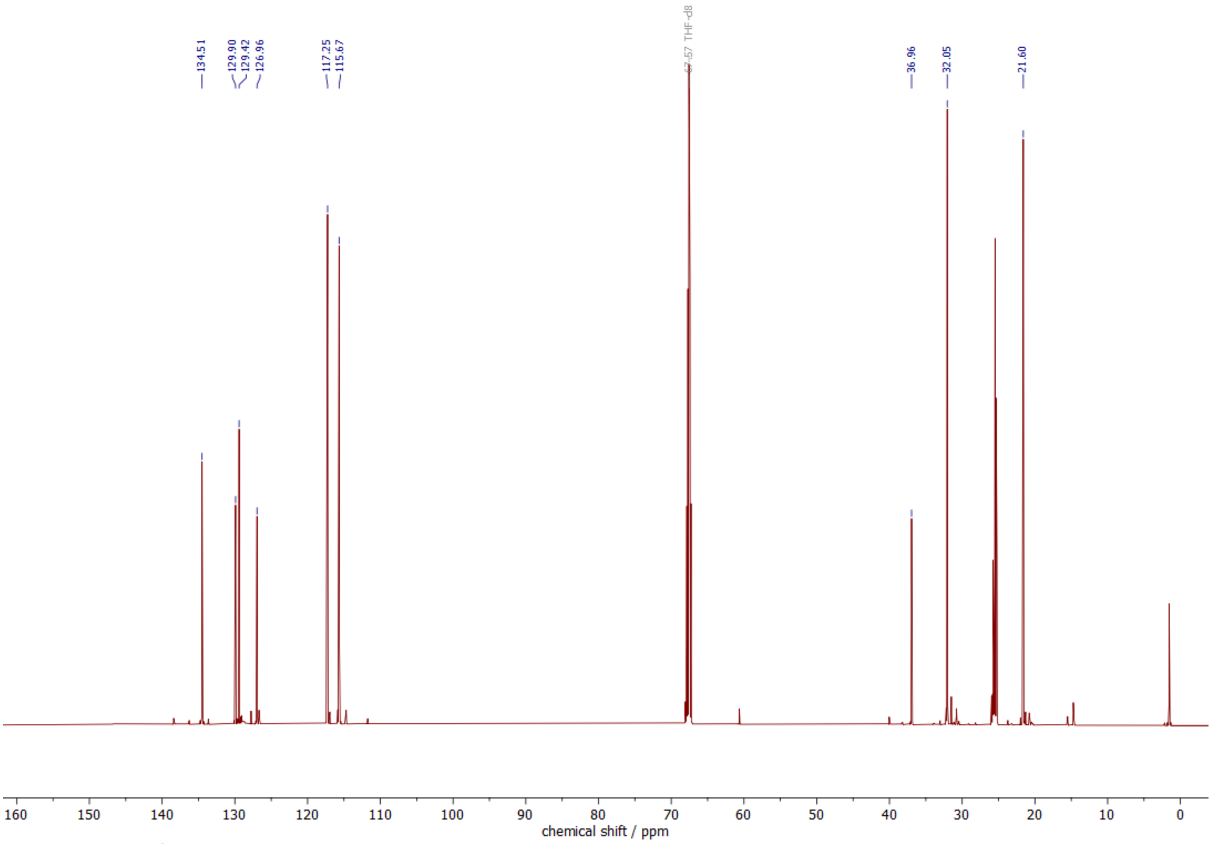

Figure S11. $^{13}C\{^{1}H\}$ NMR spectrum of **V**, THF-d_8, RT (151 MHz).

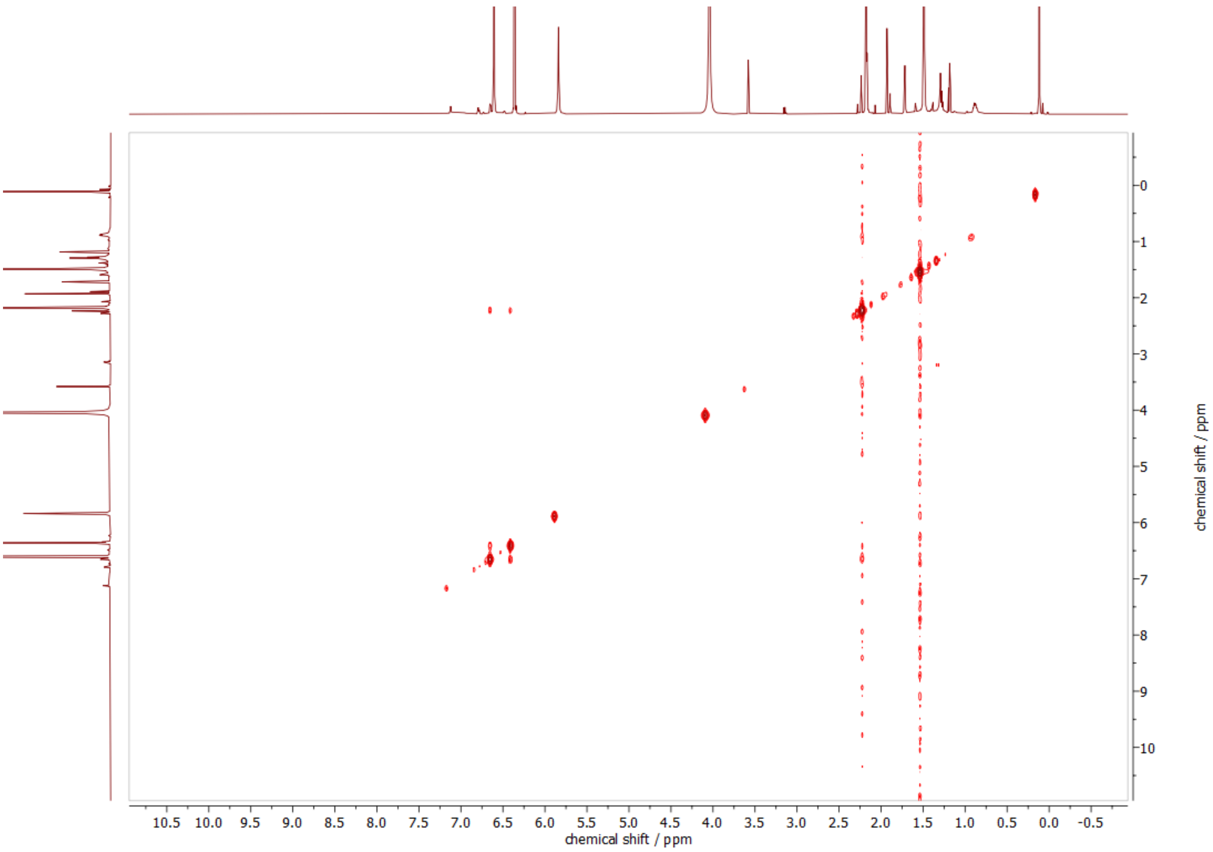

Figure S12. COSY NMR spectrum of **V**, THF-d_8, RT.

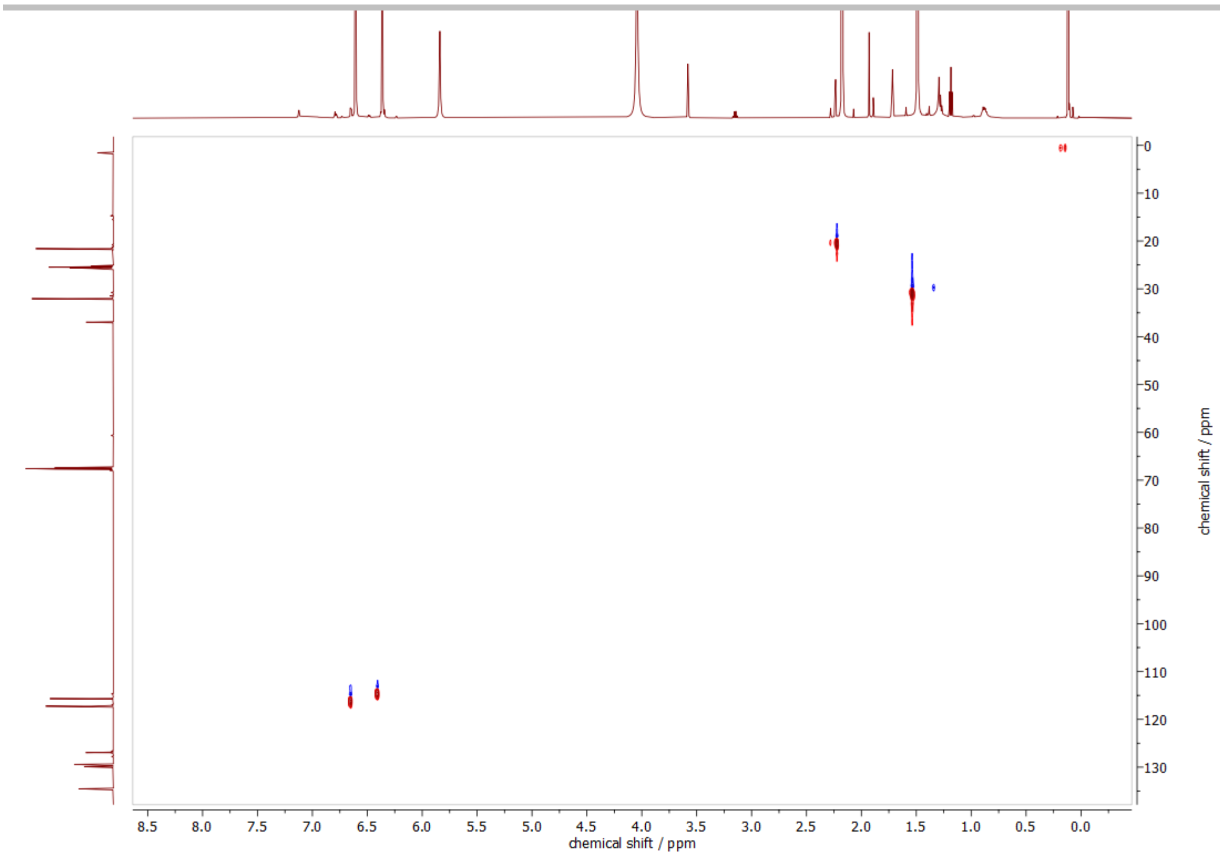

Figure S13. HSQC NMR spectrum of **V**, THF-d_8, RT.

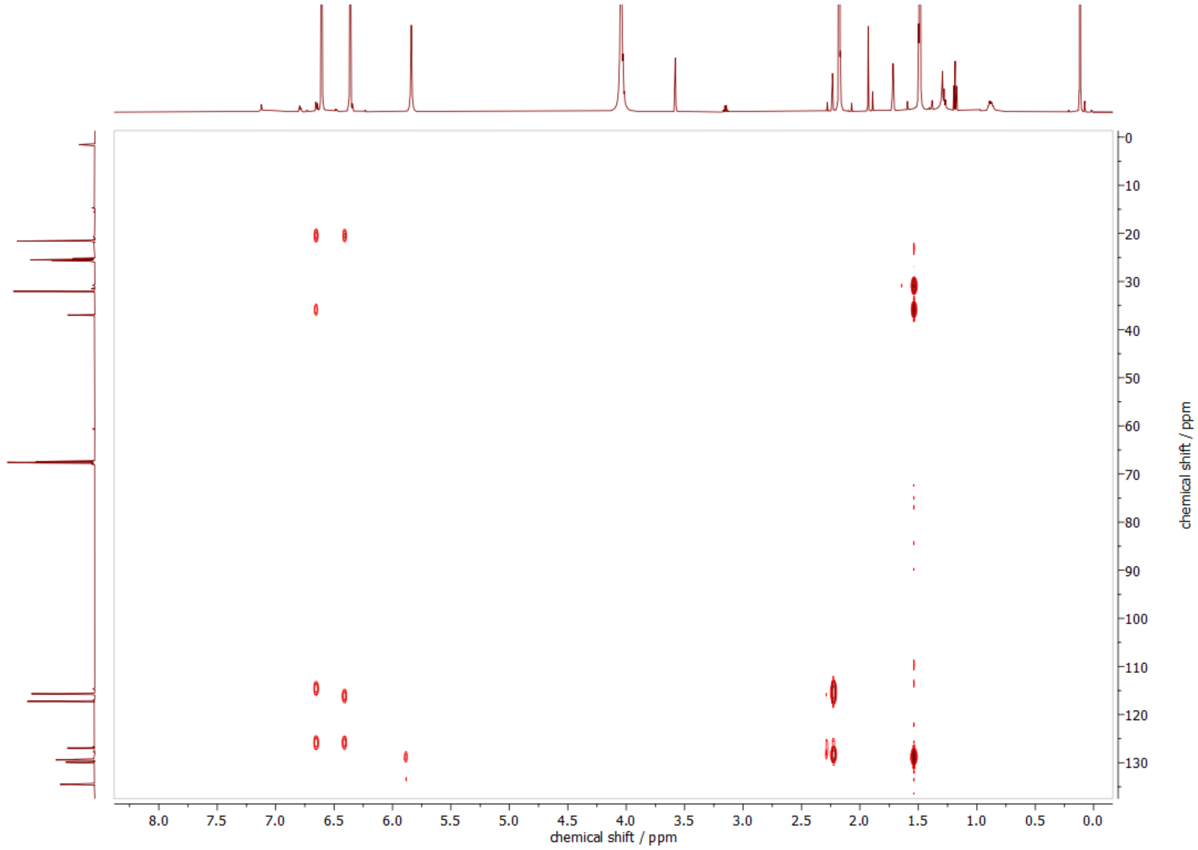

Figure S14. HMBC NMR spectrum of **V**, THF-d_8, RT.

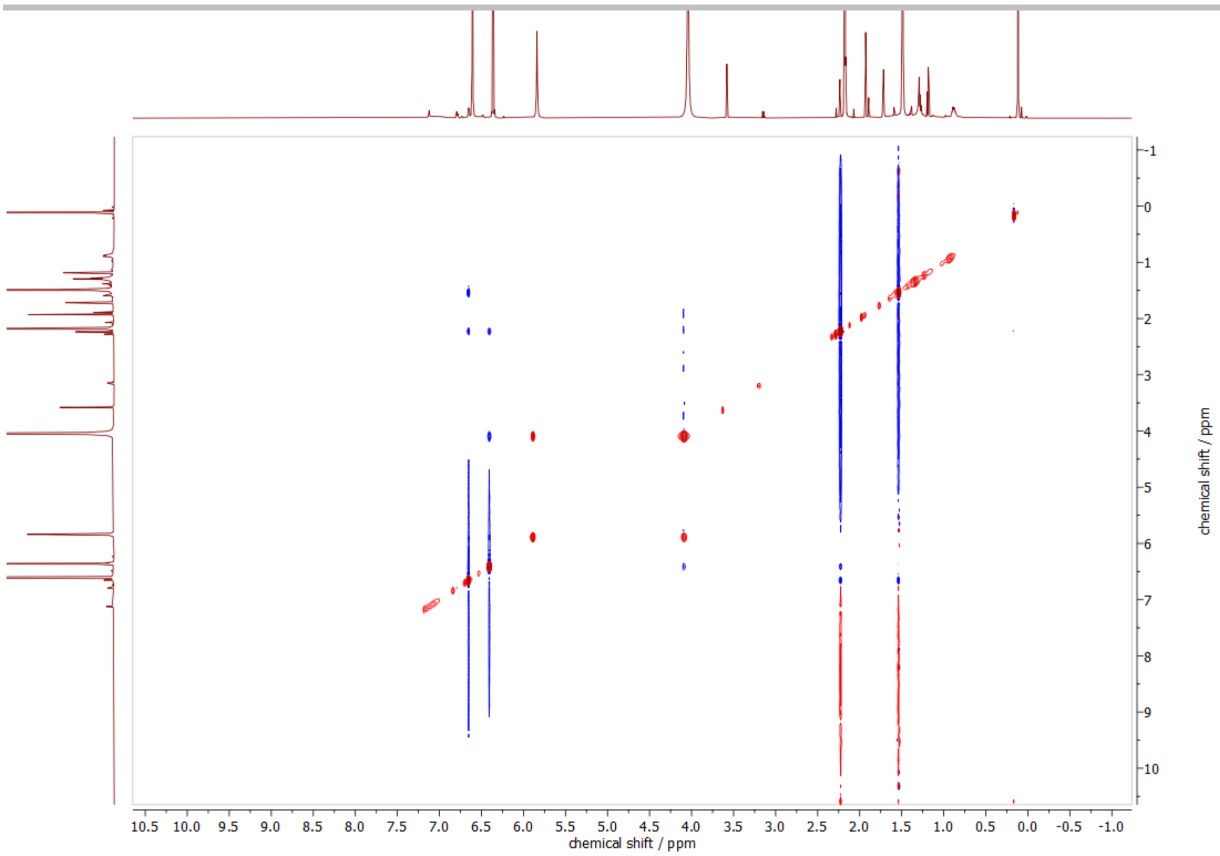

Figure S12. NOESY NMR spectrum of **V**, THF-d_8, RT.

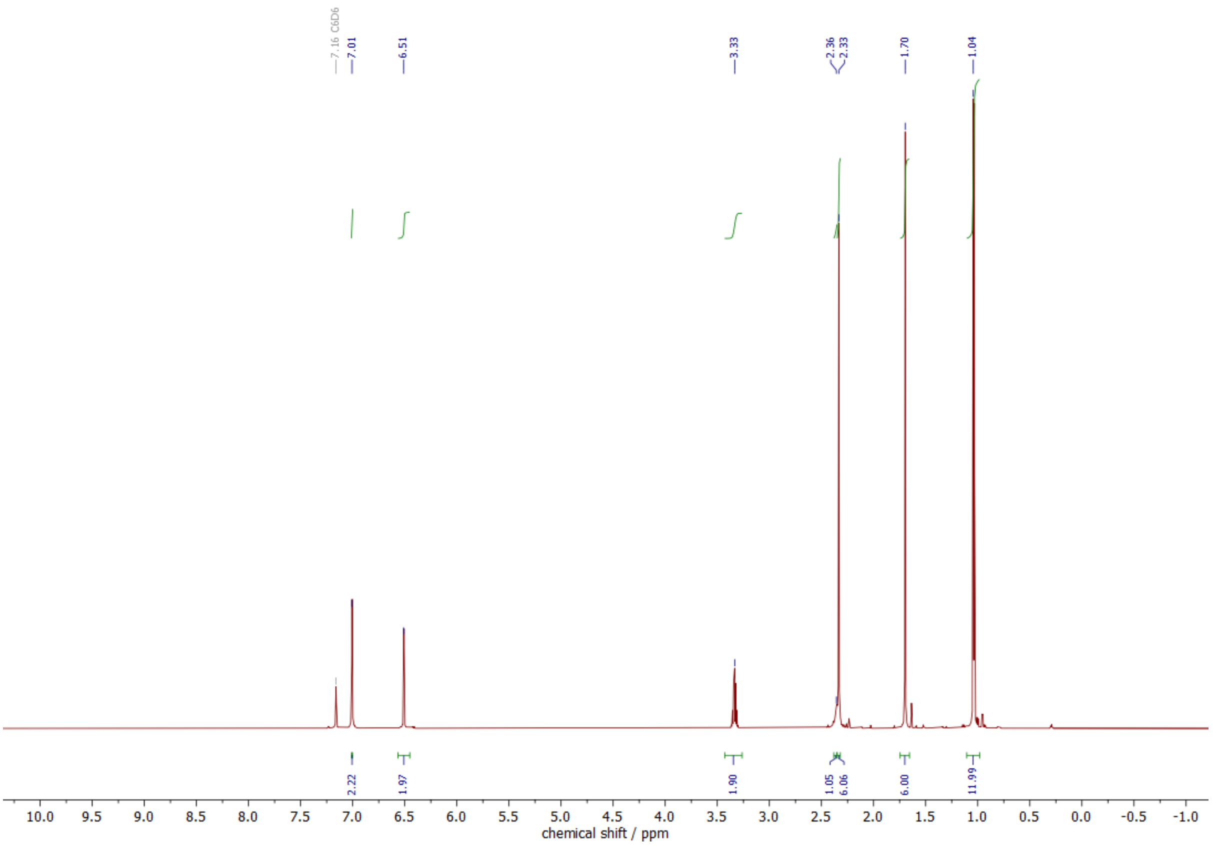

Figure S15. ^{1}H NMR spectrum of **1**, C_6D_6, RT (600 MHz).

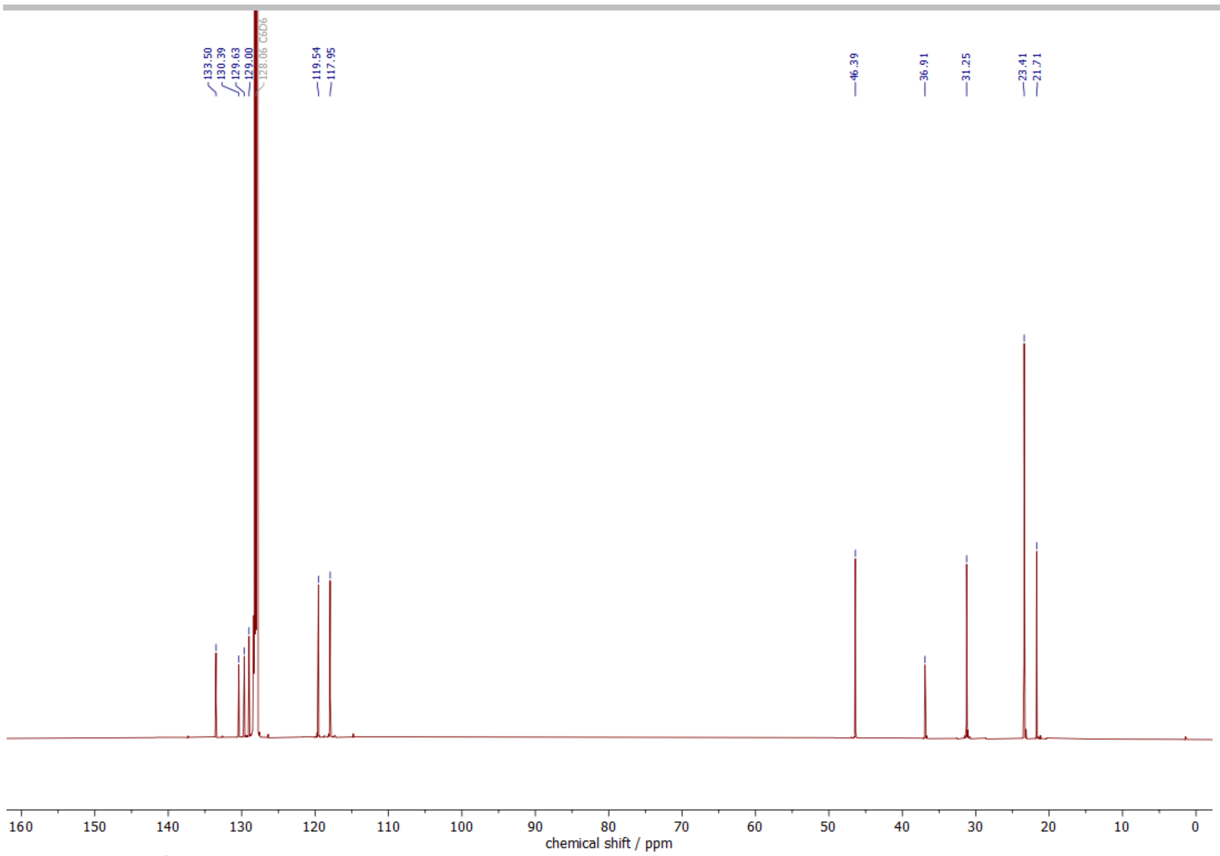

Figure S16. $^{13}C\{^1H\}$ NMR spectrum of **1**, C_6D_6, RT (151 MHz).

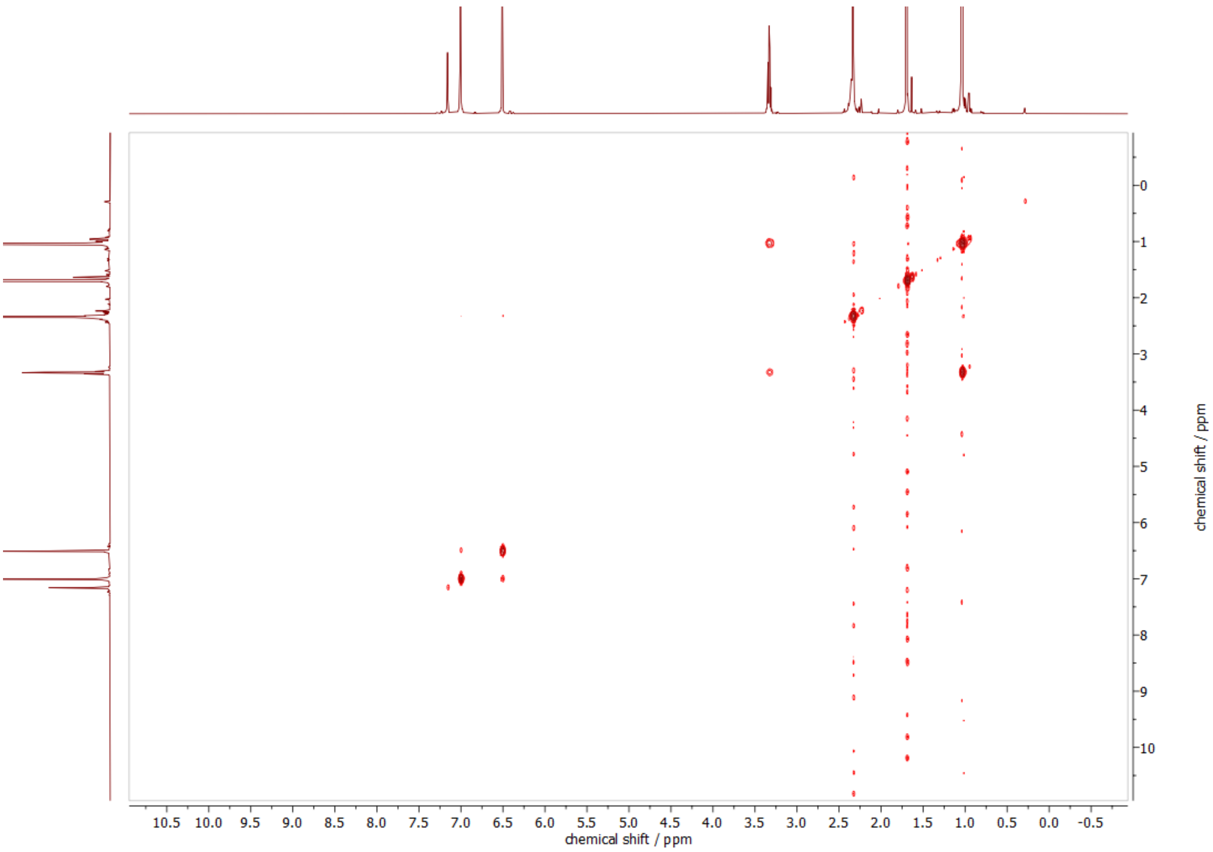

Figure S17. COSY NMR spectrum of **1**, C_6D_6, RT.

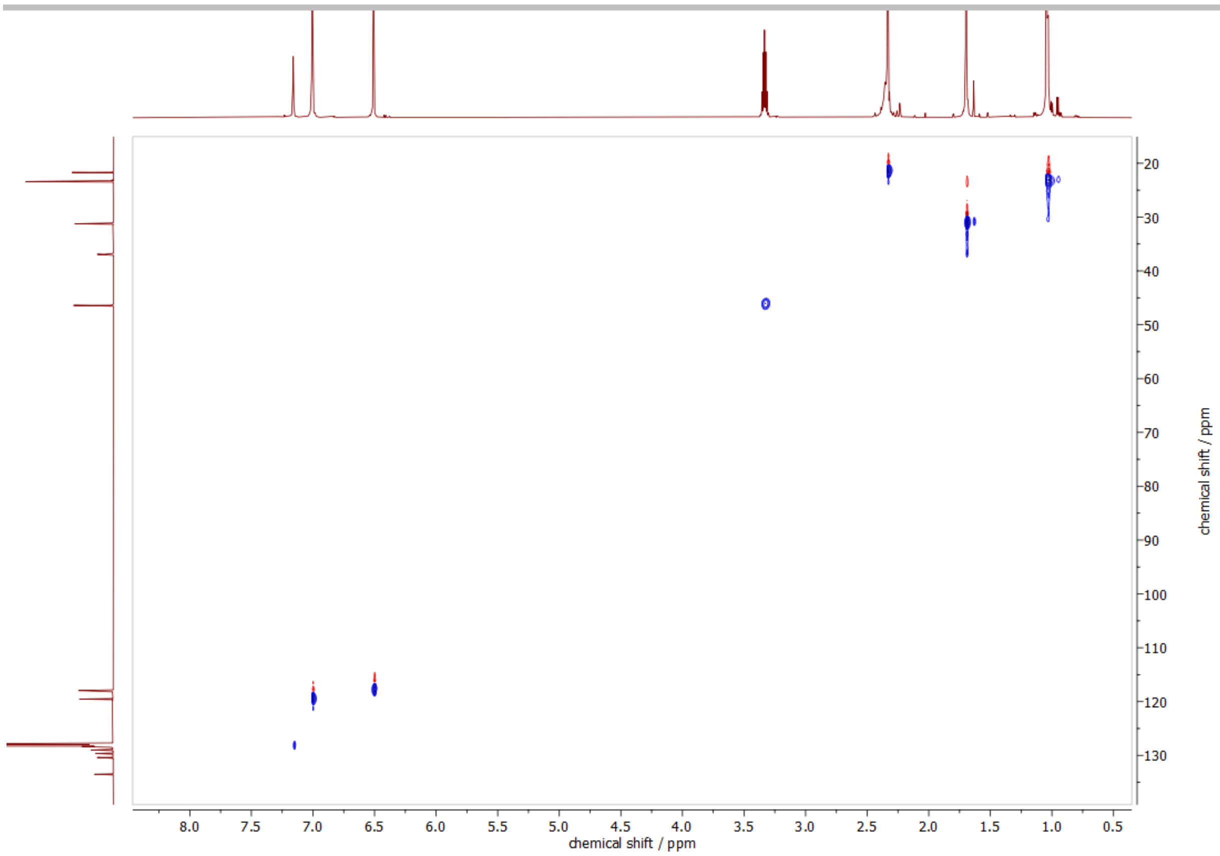

Figure S18. HSQC NMR spectrum of **1**, C_6D_6, RT.

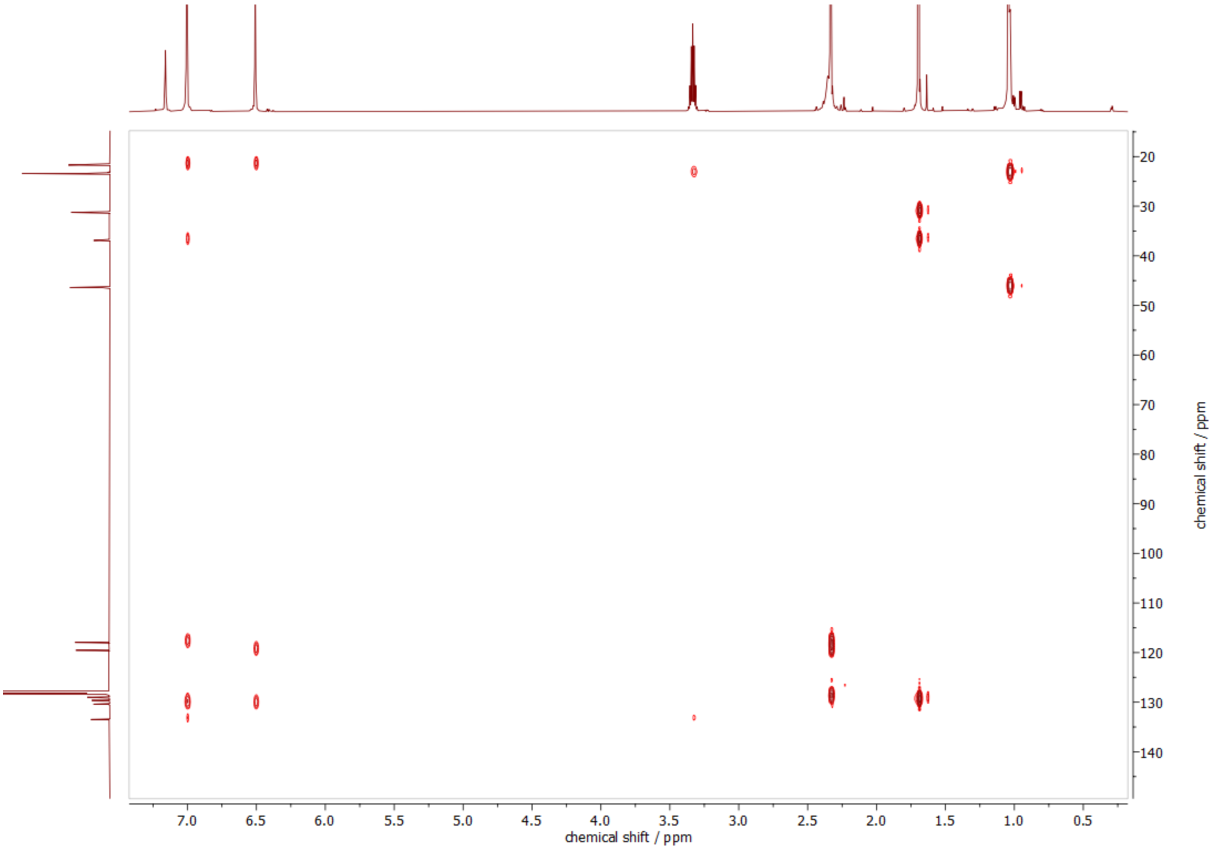

Figure S19. HMBC NMR spectrum of **1**, C_6D_6, RT.

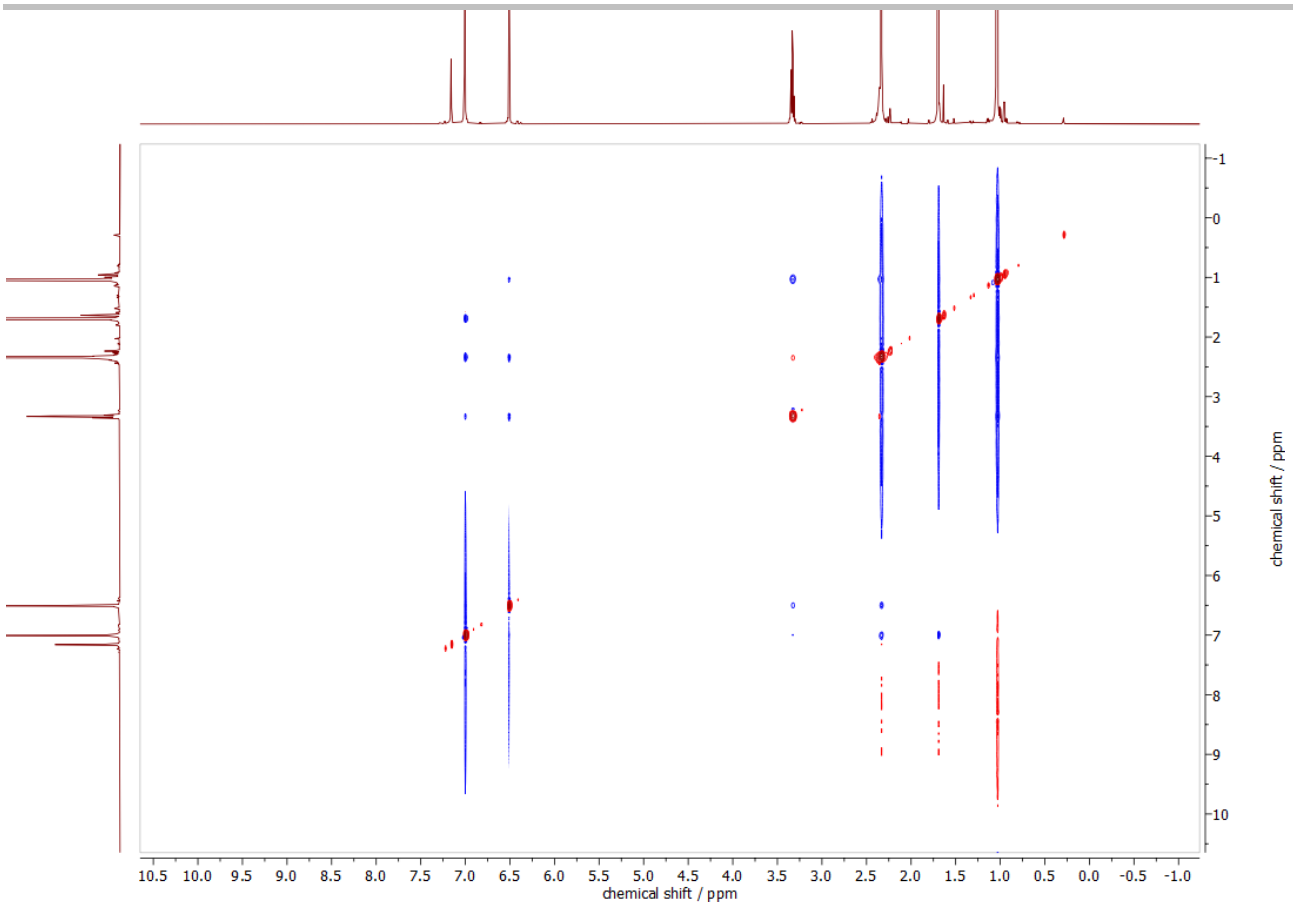

Figure S20. NOESY NMR spectrum of **1**, C_6D_6, RT.

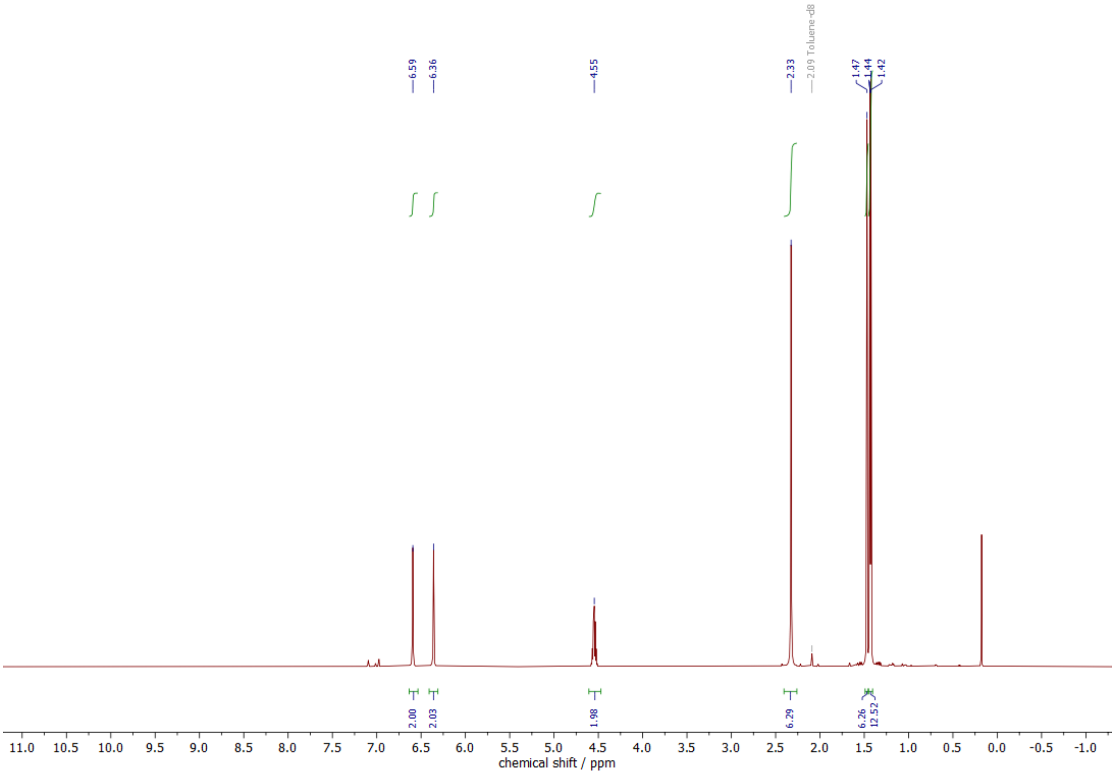

Figure S21. ^{1}H NMR spectrum of **2**, Tol-d_8, RT (600 MHz).

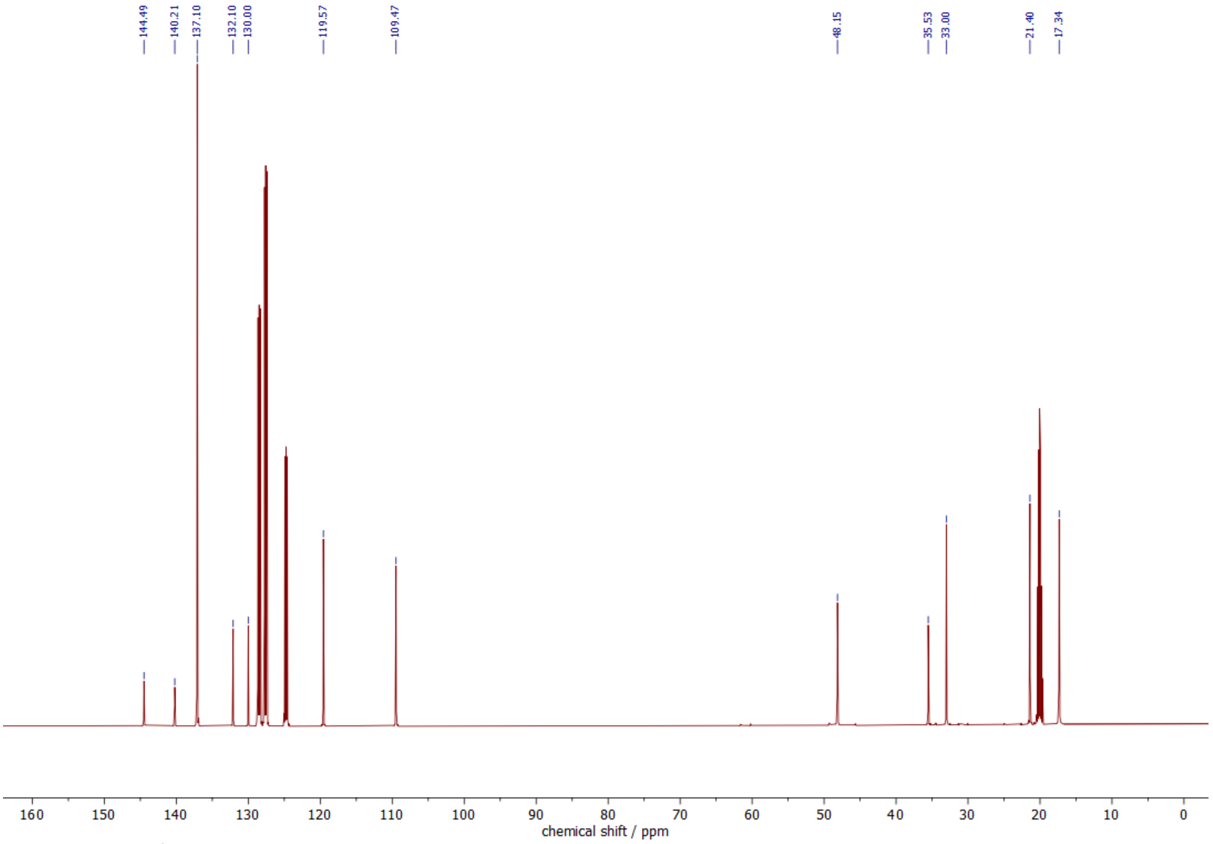

Figure S22. $^{13}C\{H\}$ NMR spectrum of **2**, Tol-d_8, RT (151 MHz).

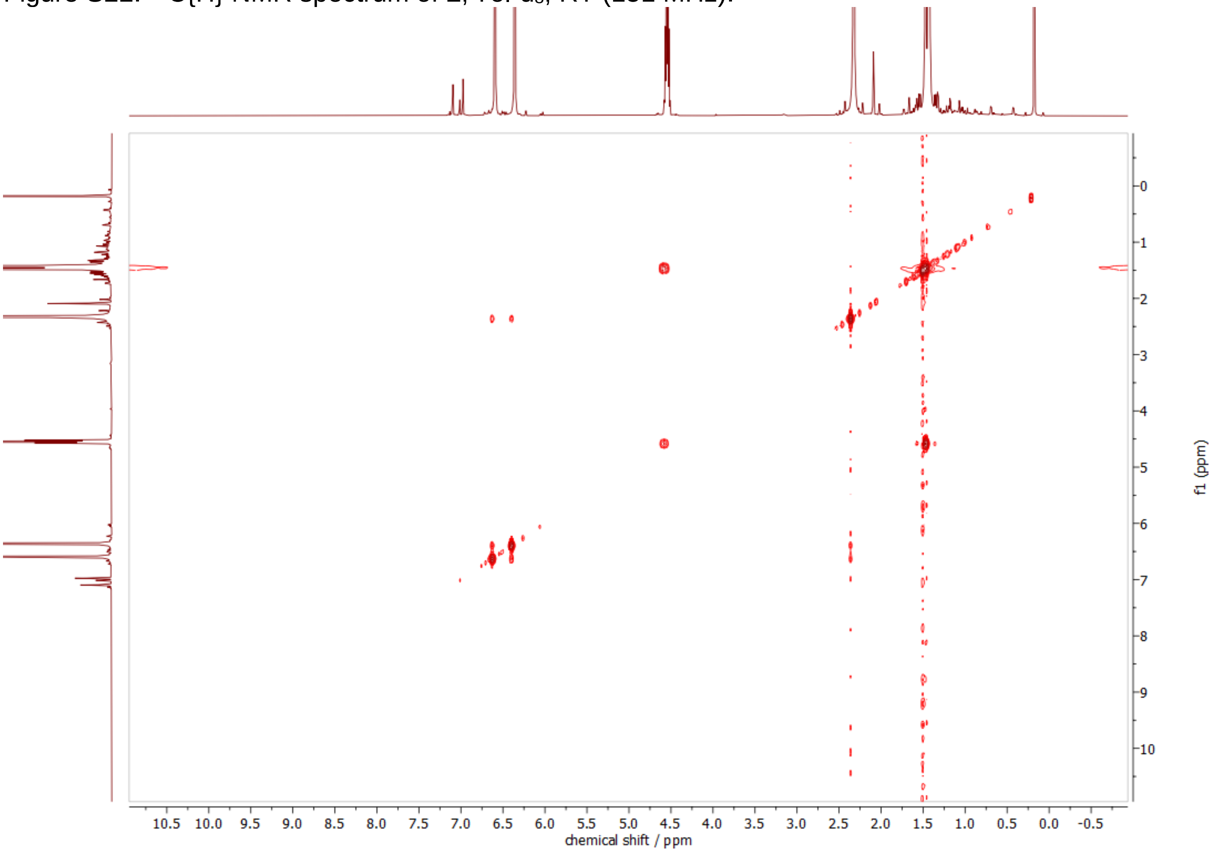

Figure S23. COSY NMR spectrum of **2**, Tol-d_8, RT.

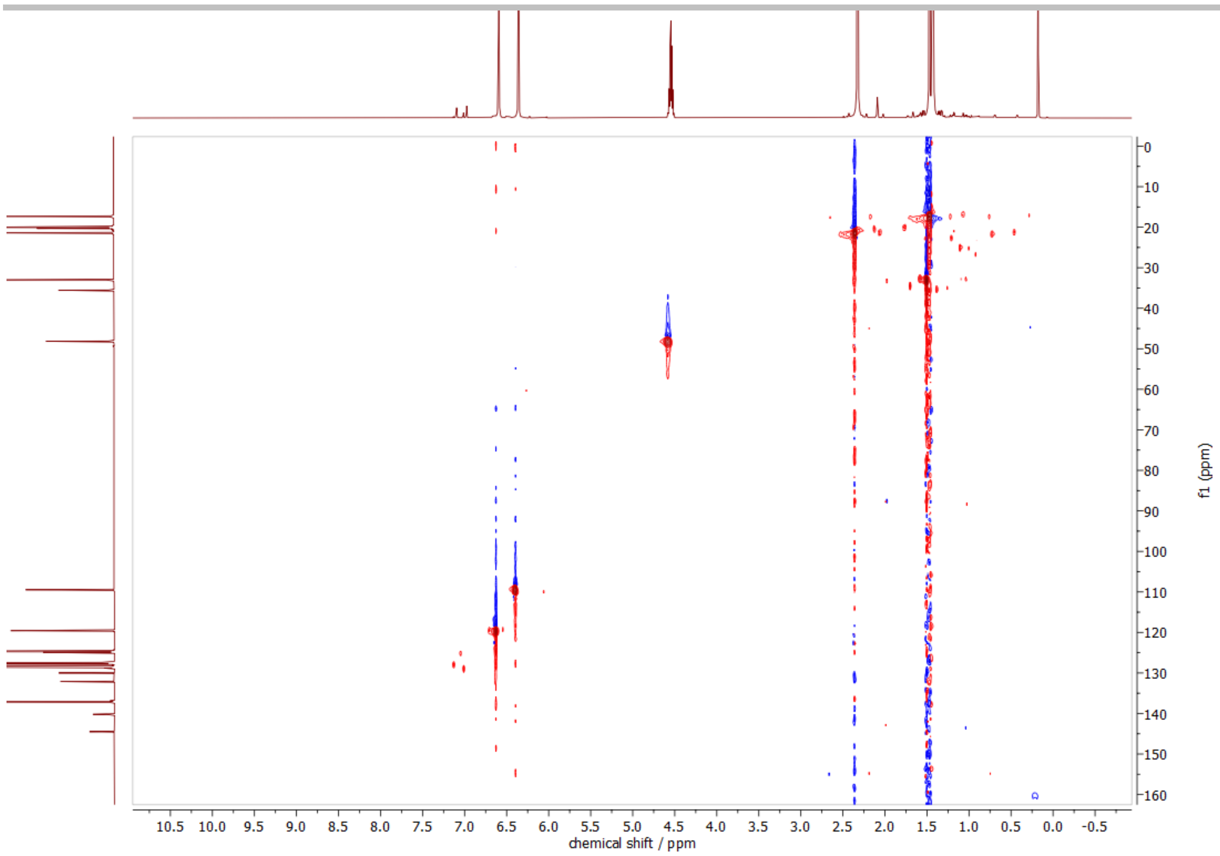

Figure S24. HSQC NMR spectrum of **2**, Tol-d8, RT.

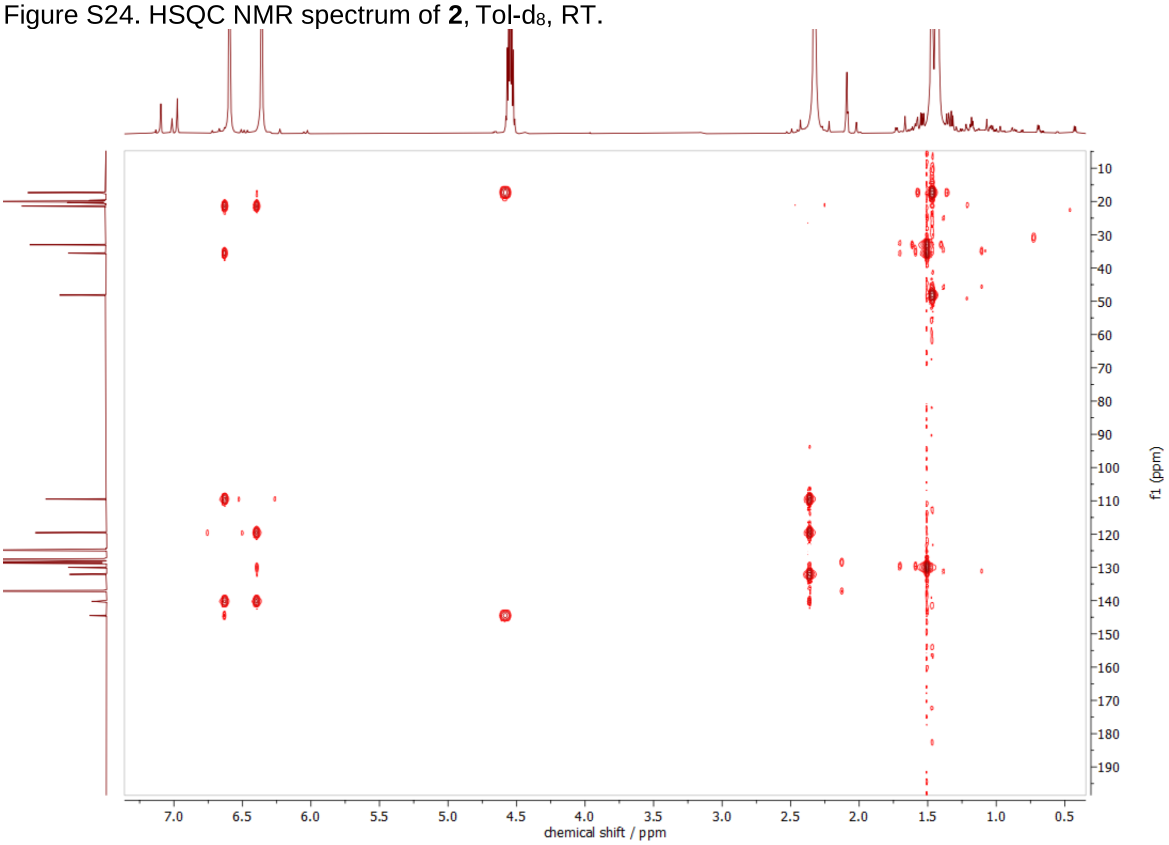

Figure S25. HMBC NMR spectrum of **2**, Tol-d8, RT.

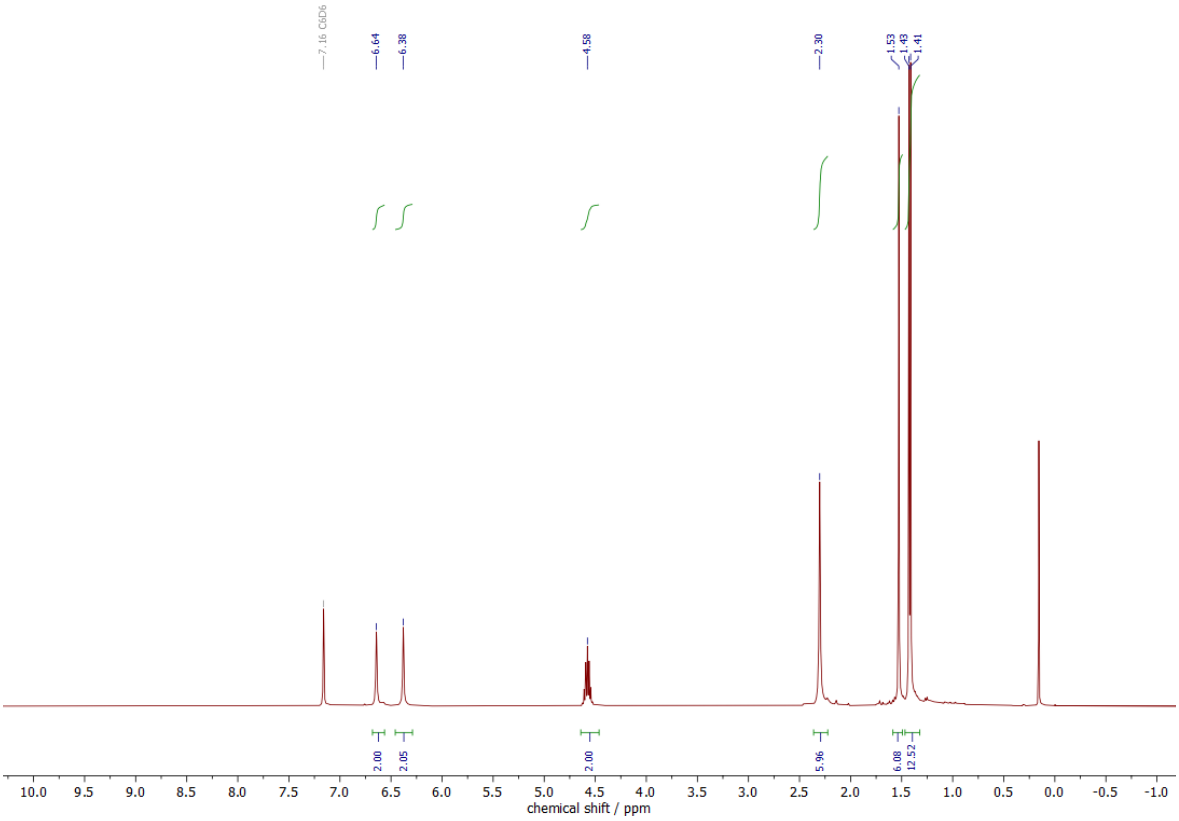

Figure S26. ^{1}H NMR spectrum of **2**, C_6D_6, RT (600 MHz).

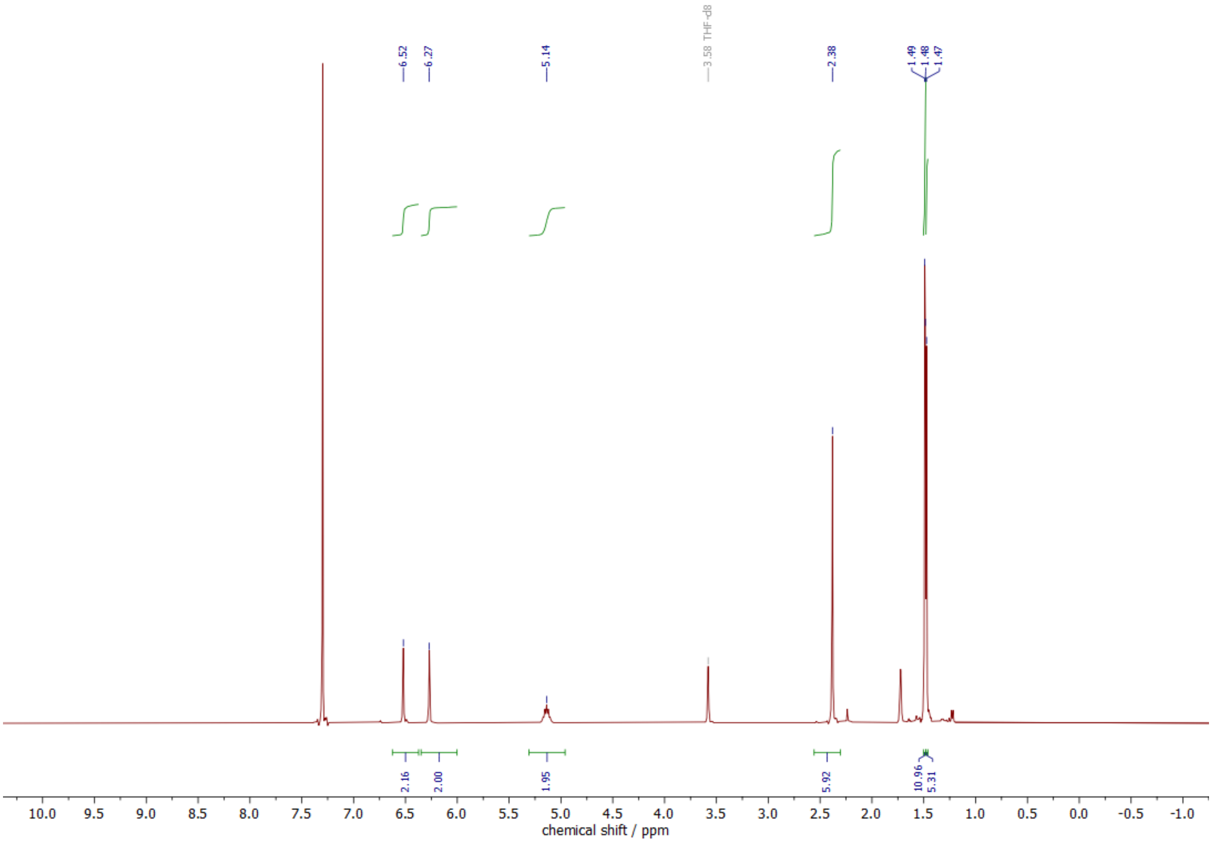

Figure S27. ^{1}H NMR spectrum of **2**, THF-d_8, RT (600 MHz).

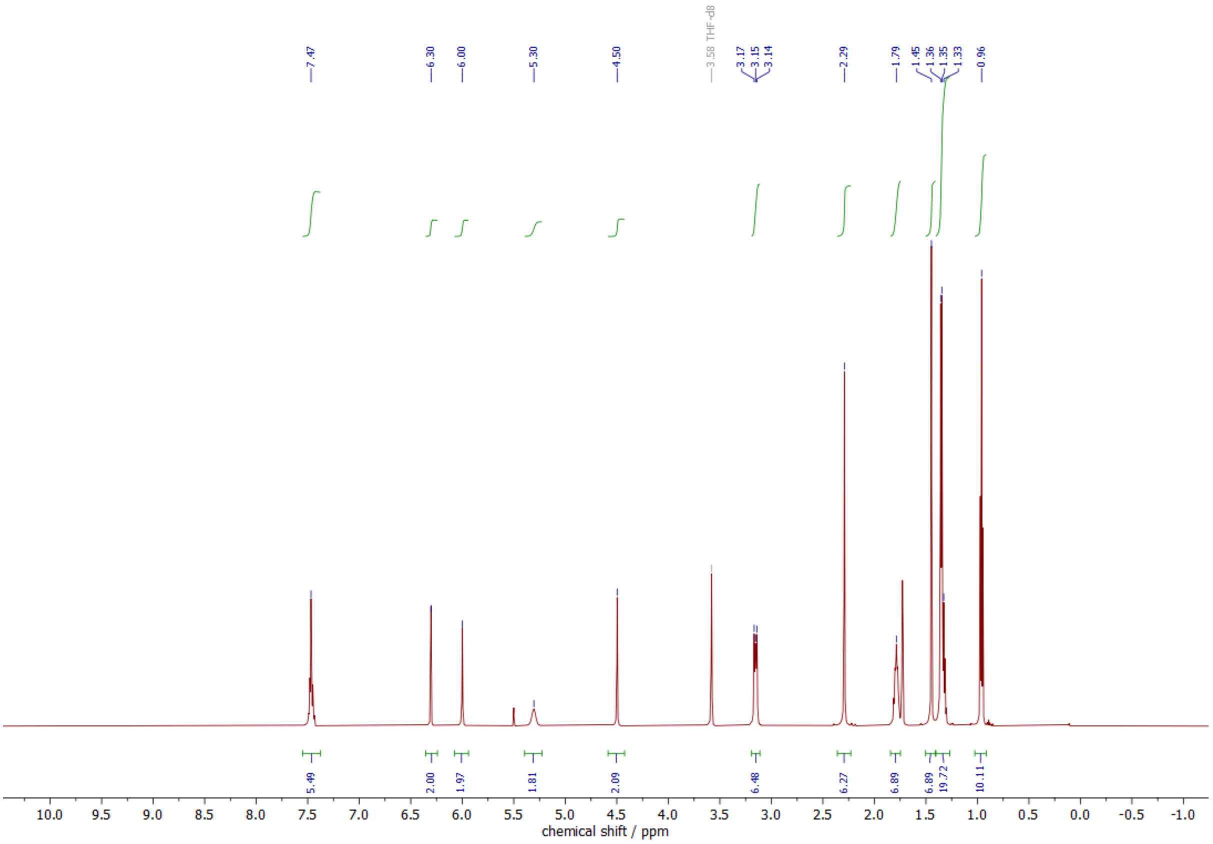

Figure S28. ^{1}H NMR spectrum of **3**, THF-d_8, RT (600 MHz).

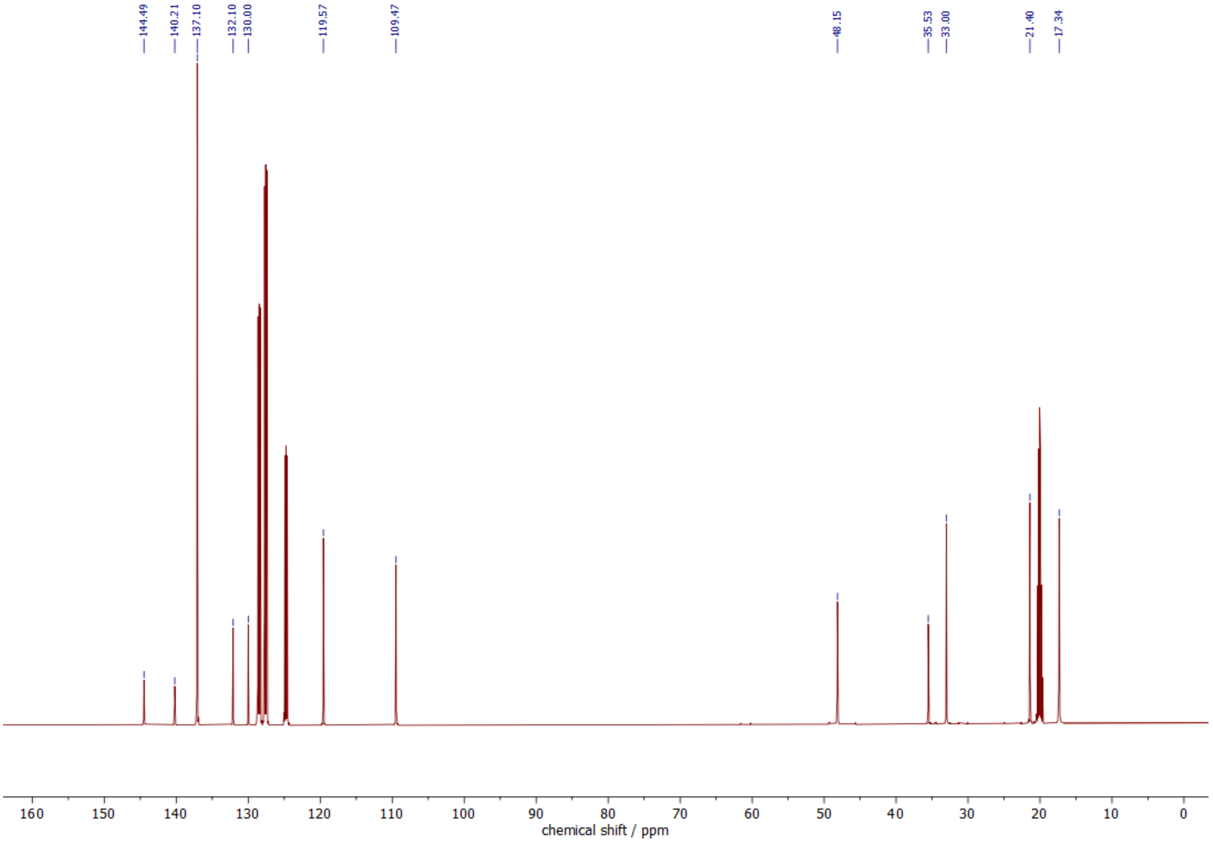

Figure S29. $^{13}C\{^{1}H\}$ NMR spectrum of **3**, THF-d_8, RT (151 MHz).

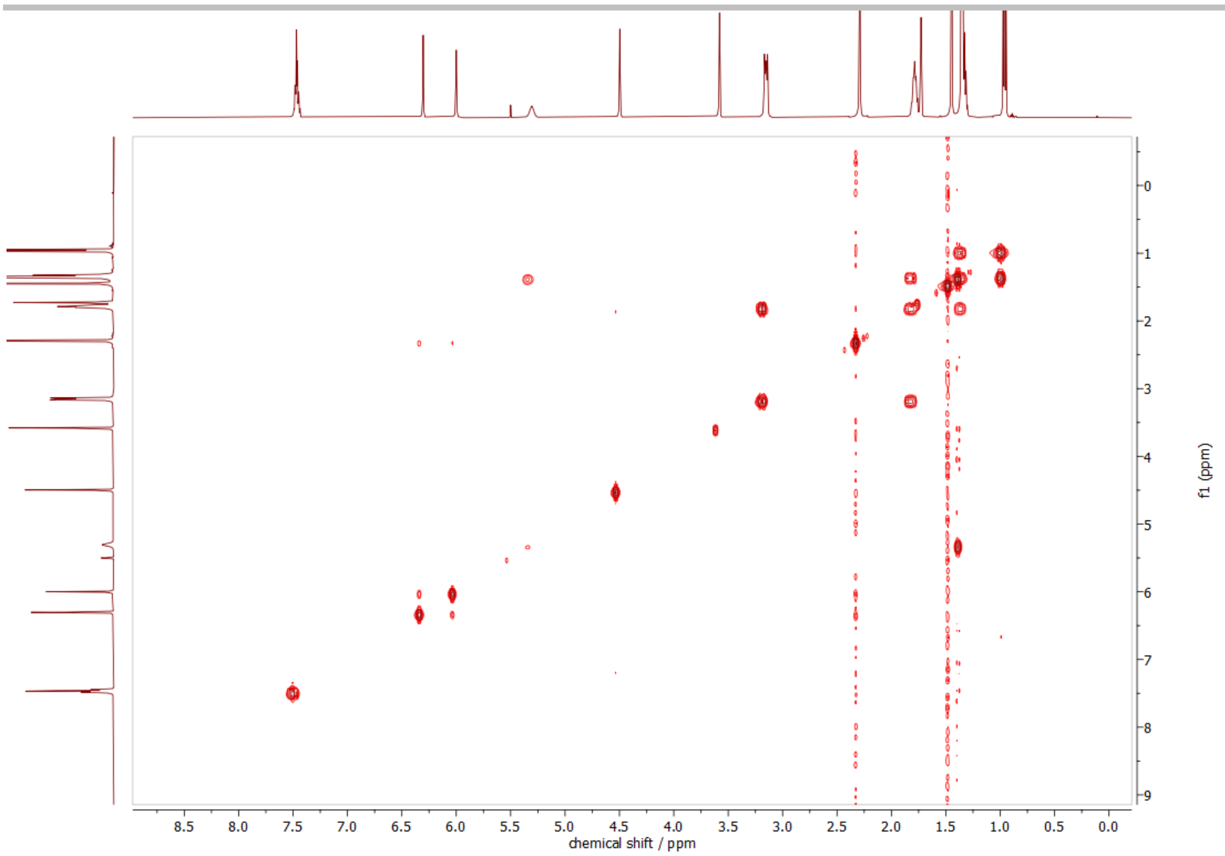

Figure S30. COSY NMR spectrum of **3**, THF-d_8, RT.

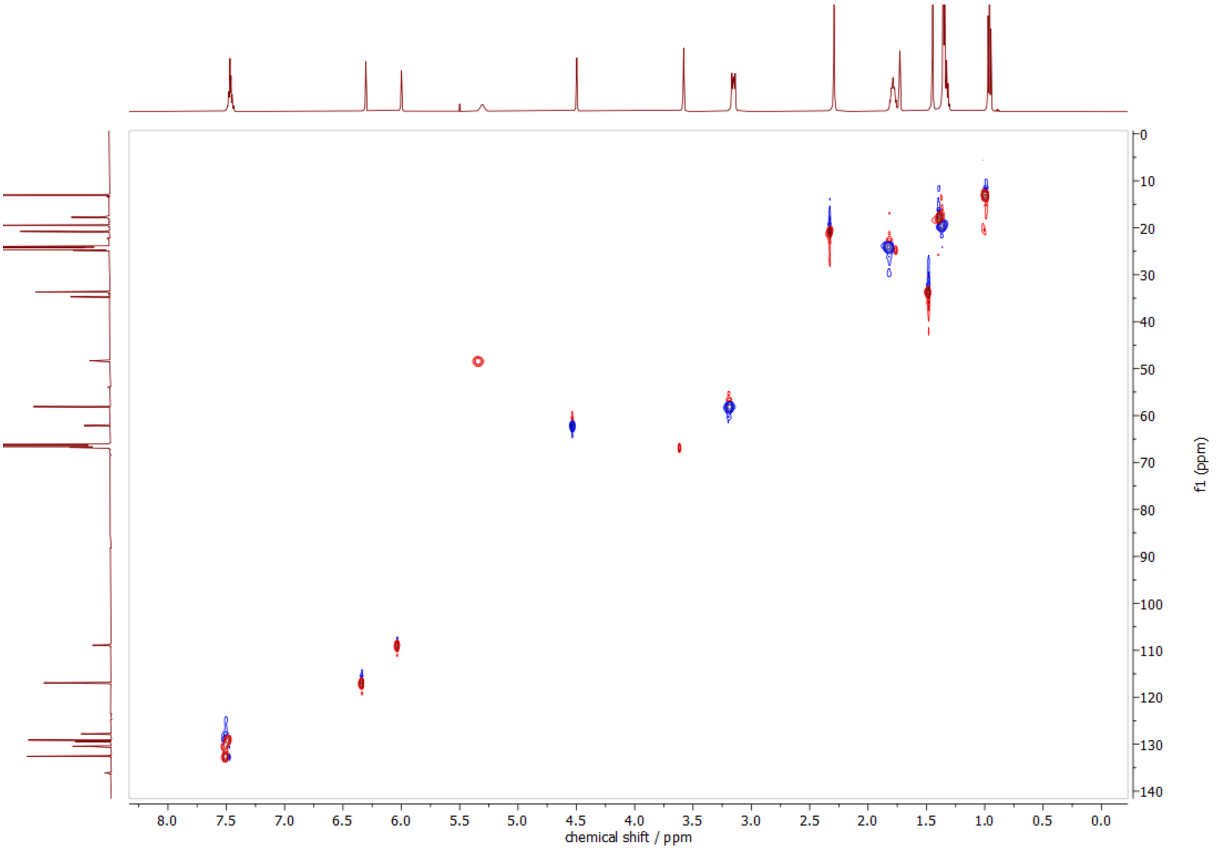

Figure S31. HSQC NMR spectrum of **3**, THF-d_8, RT.

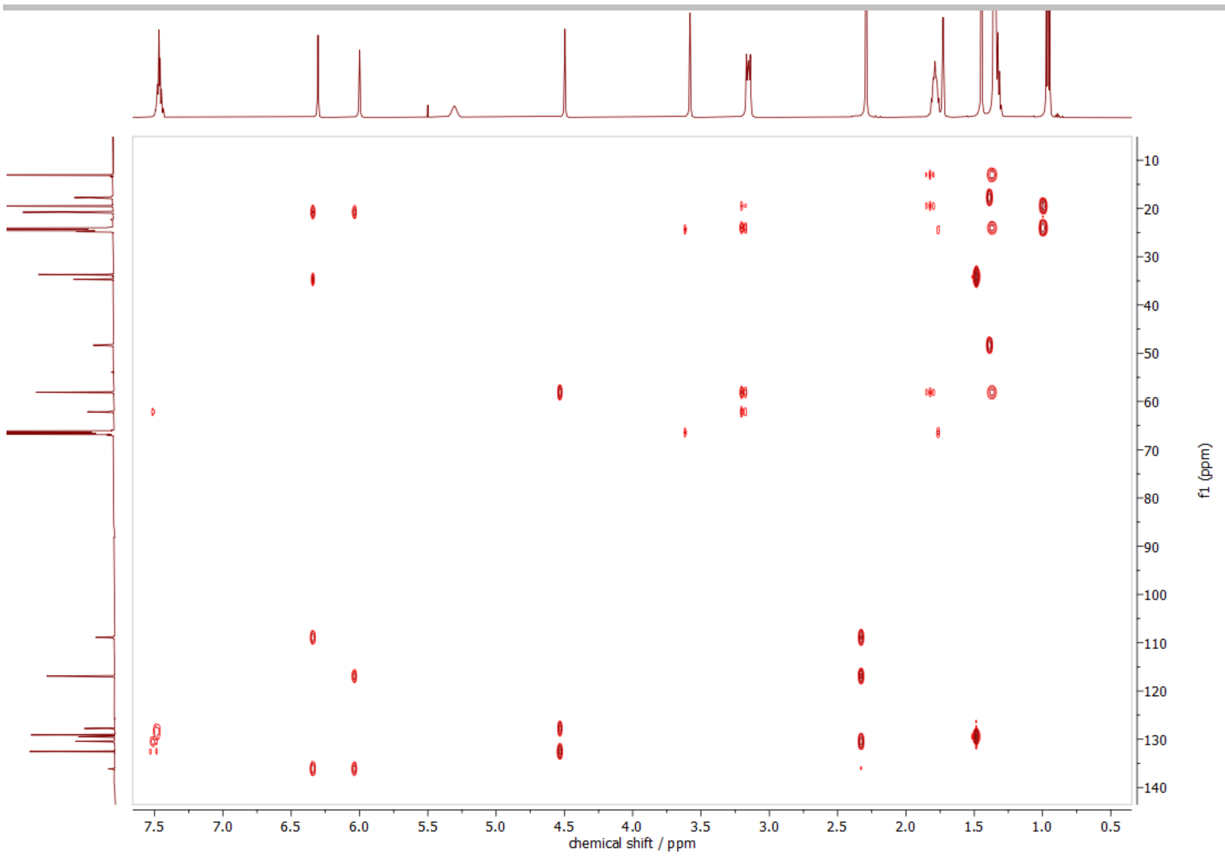

Figure S32. HMBC NMR spectrum of **3**, THF-d_8, RT.

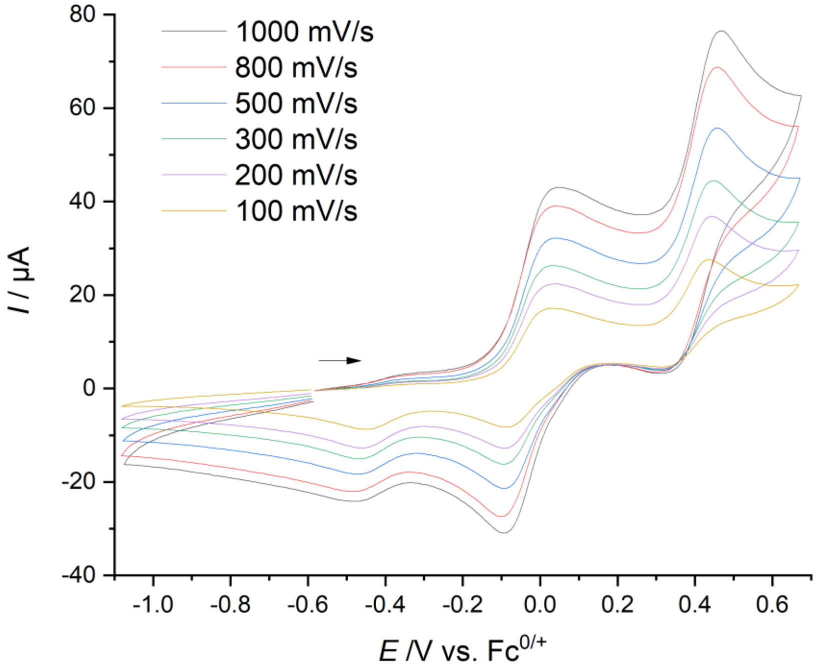

Figure S33. Cyclic voltammogram (MeCN, 0.1 M $[Bu_4N][PF_6]$) of a 1 mM solution of **3**. Potentials are referenced to $[Cp_2Fe]^{+/0}$.

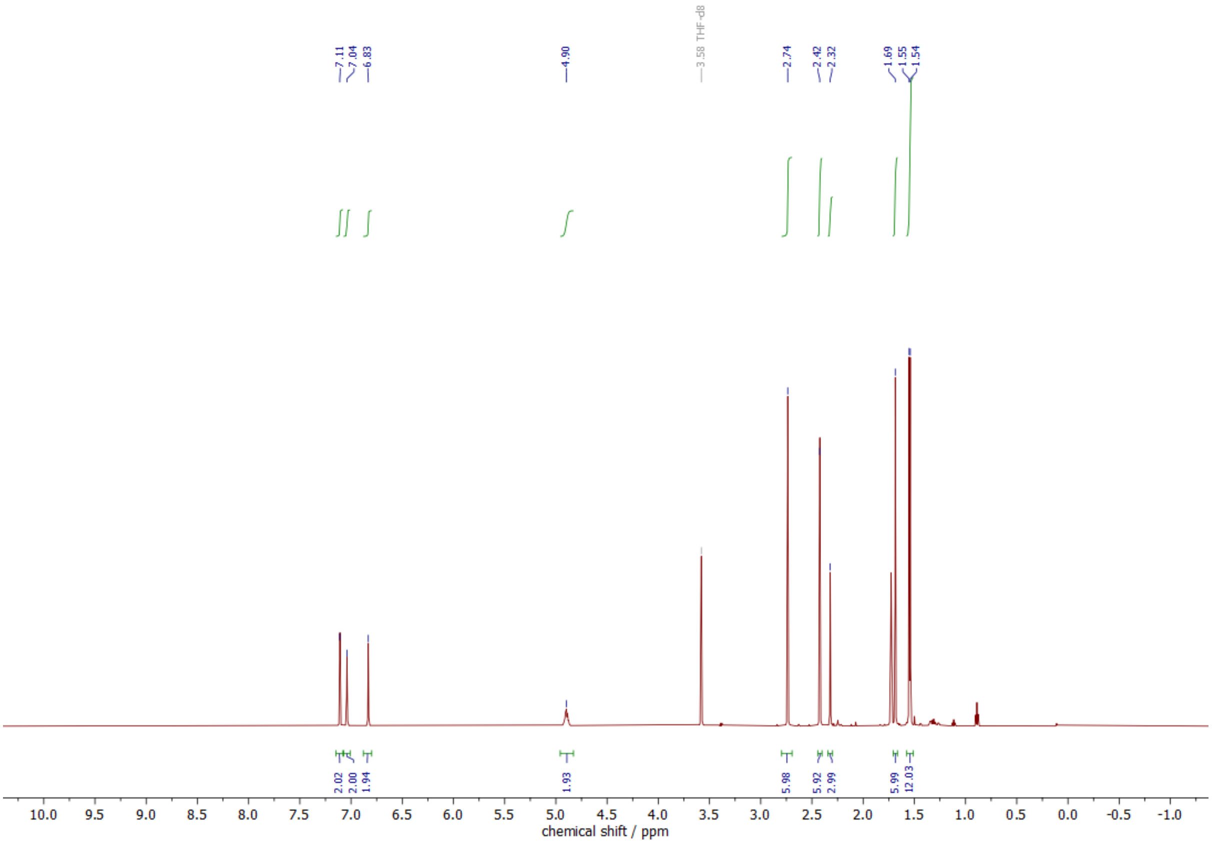

Figure S34. ^{1}H NMR spectrum of **4**, THF-d_8, RT (600 MHz).

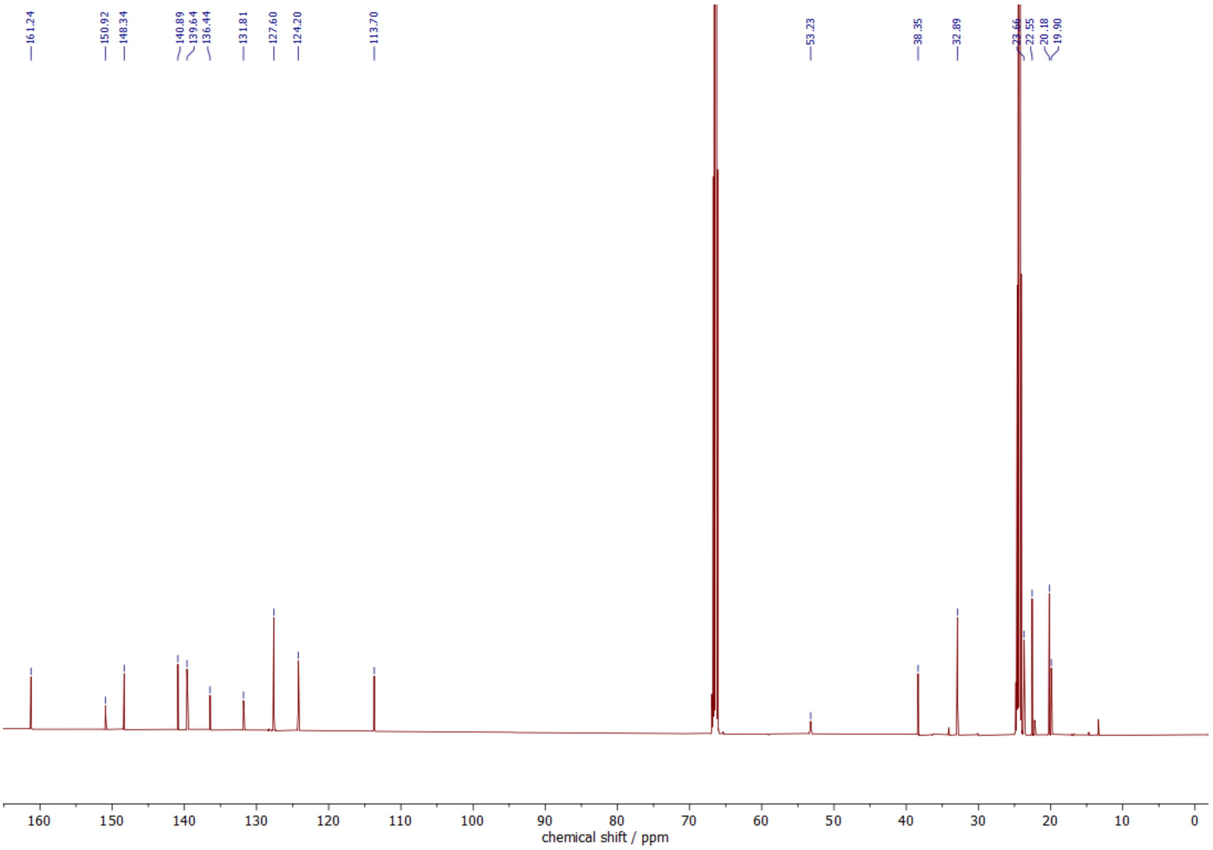

Figure S35. $^{13}C\{^{1}H\}$ NMR spectrum of **4**, THF-d_8, RT (151 MHz).

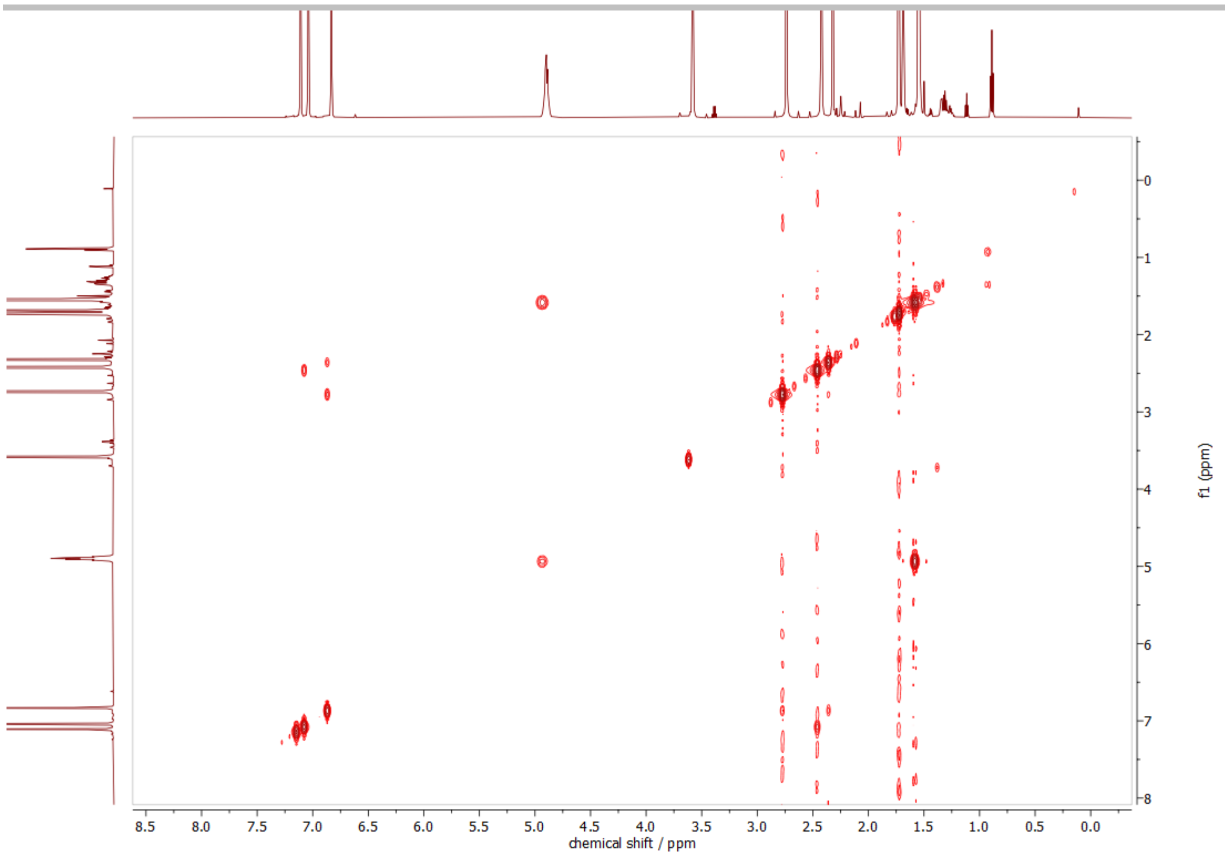

Figure S36. COSY NMR spectrum of **4**, THF-d_8, RT.

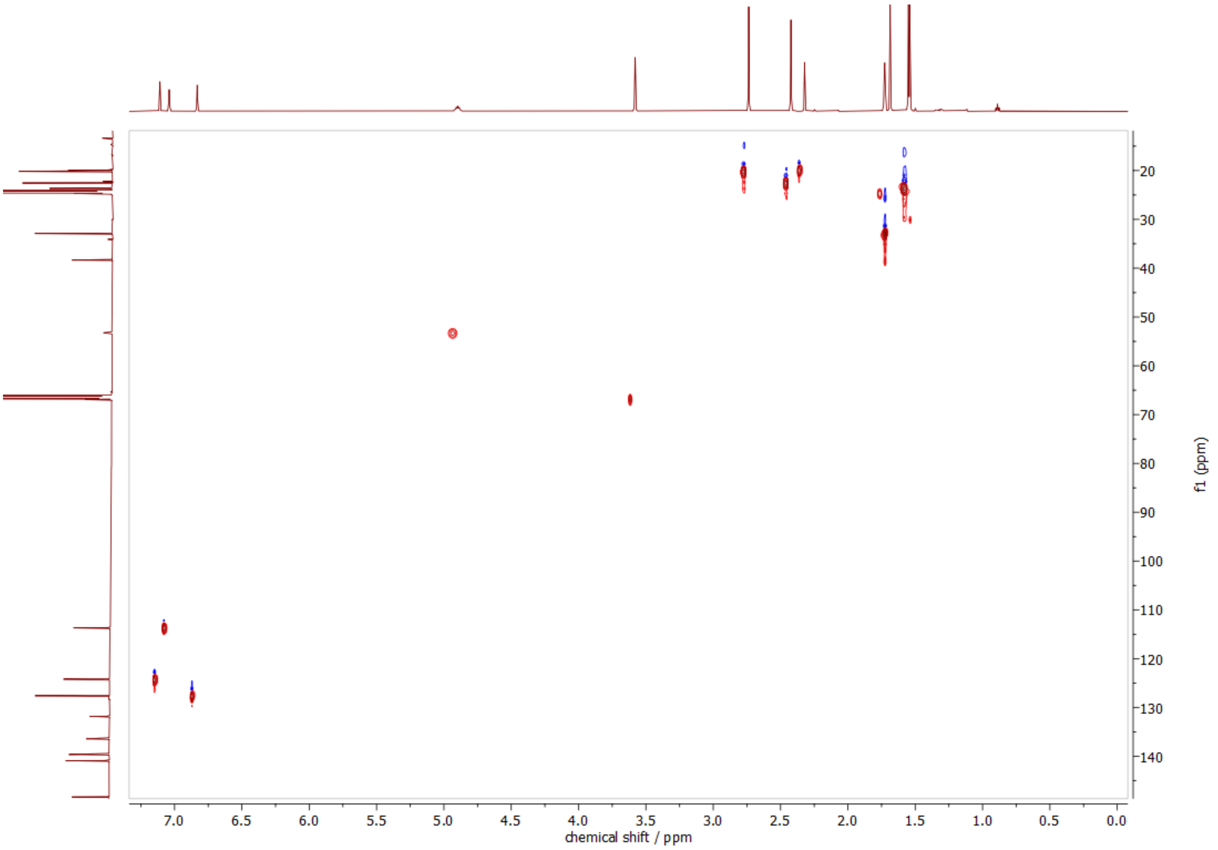

Figure S37. HSQC NMR spectrum of **4**, THF-d_8, RT.

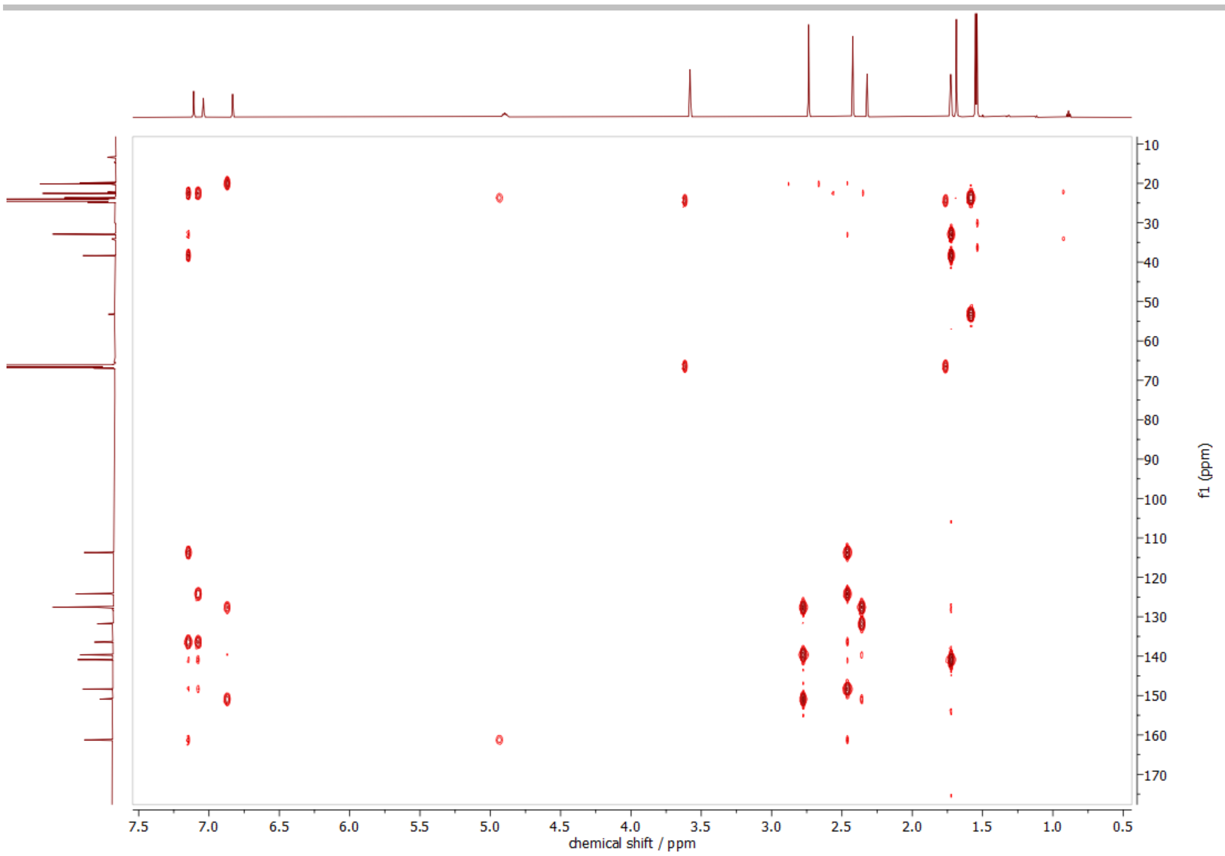

Figure S38. HMBC NMR spectrum of **4**, THF-d_8, RT.

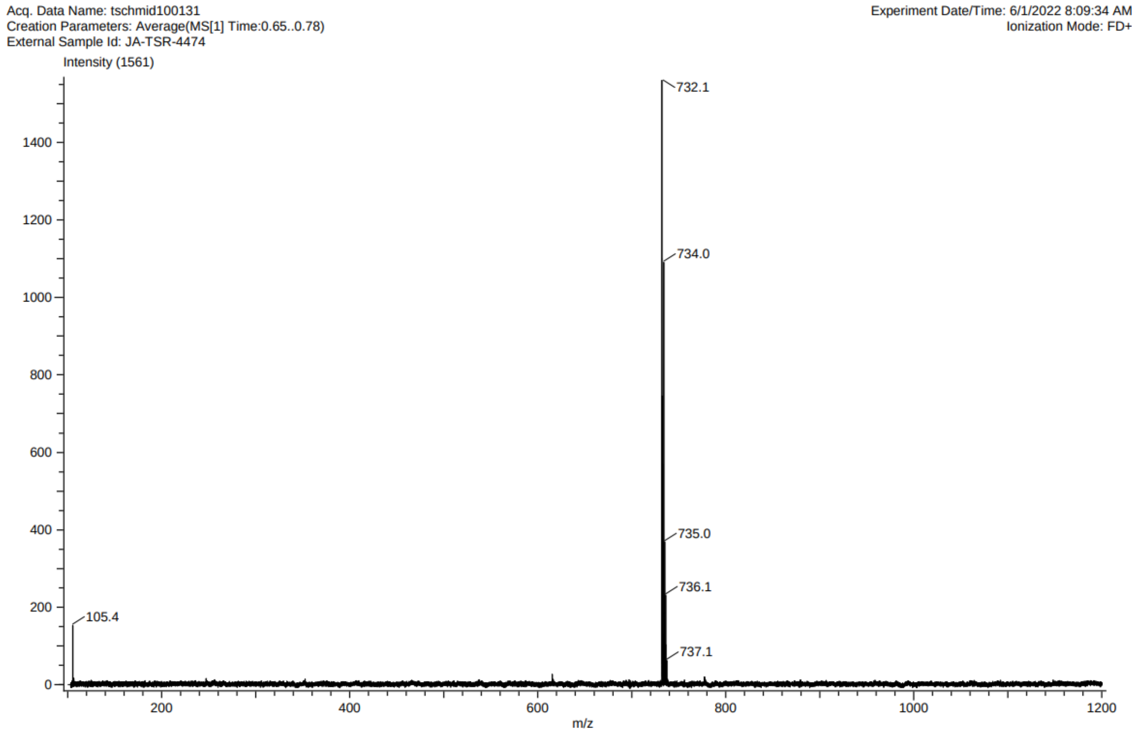

Figure S39. LIFDI-MS spectrum of **4**.

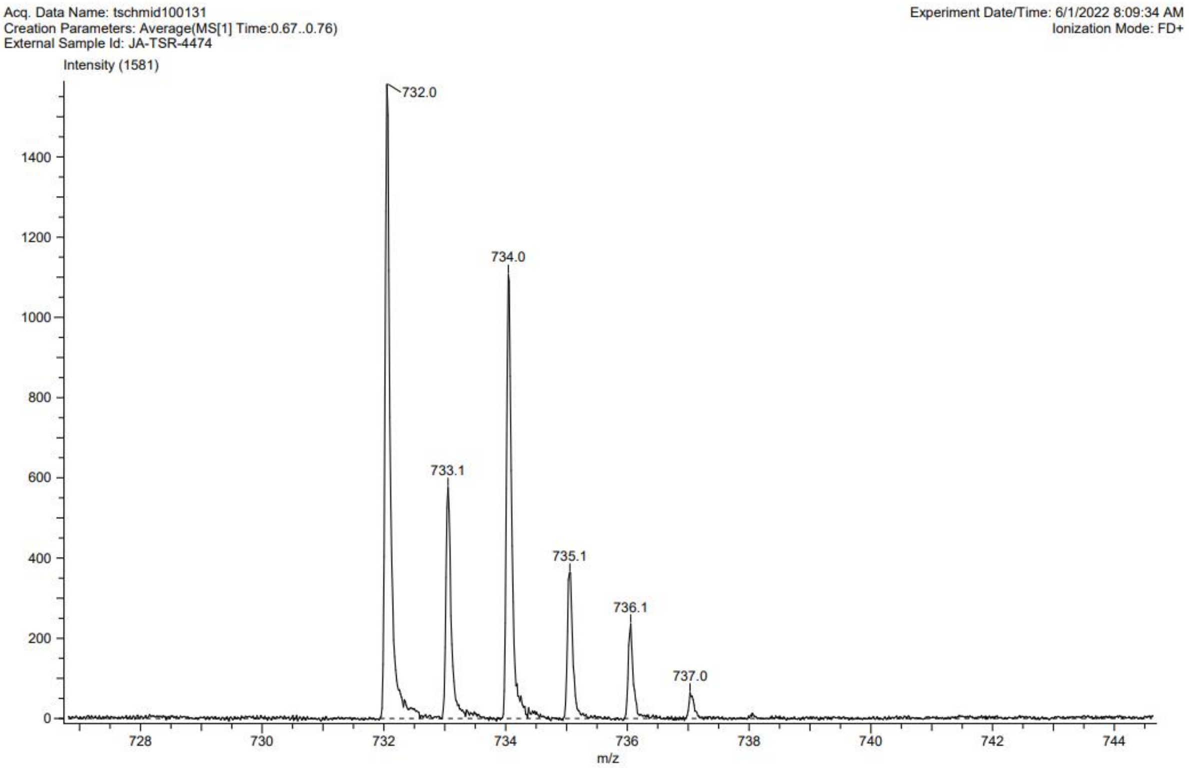

Figure S40. LIFDI-MS spectrum of **4**.

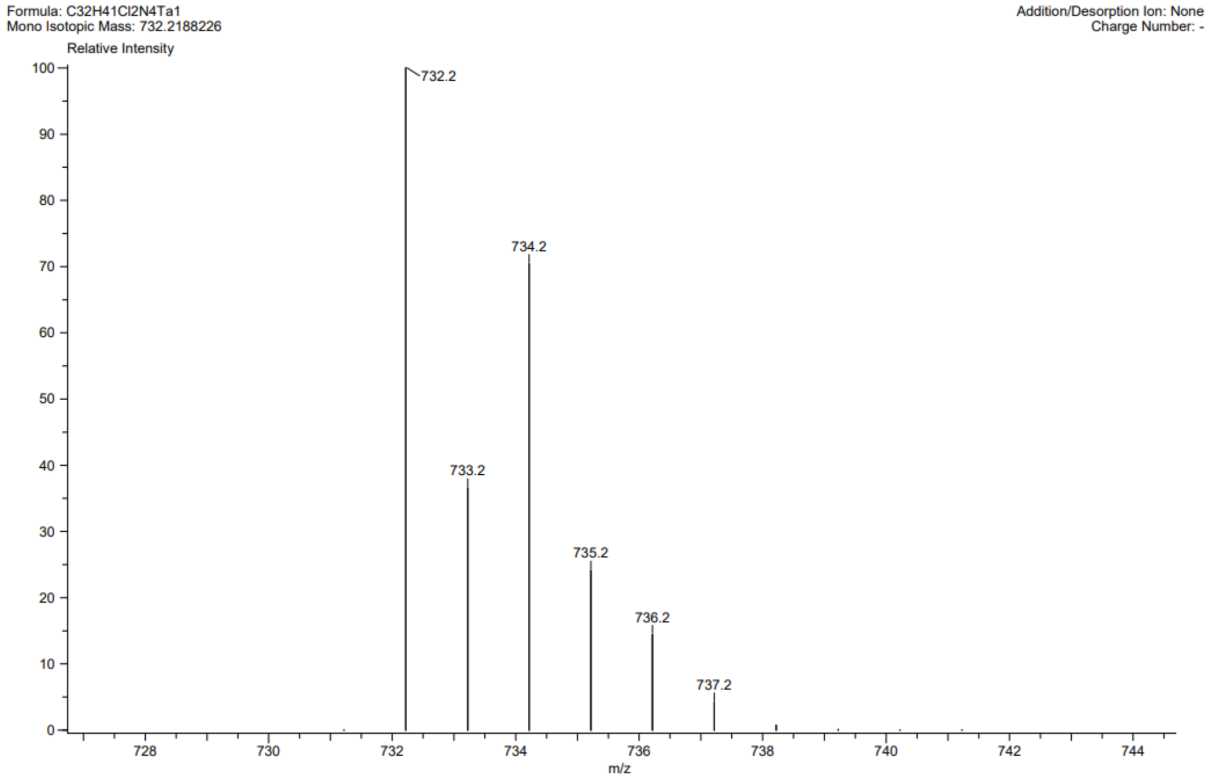

Figure S41. Calculated isotope pattern of **4**.

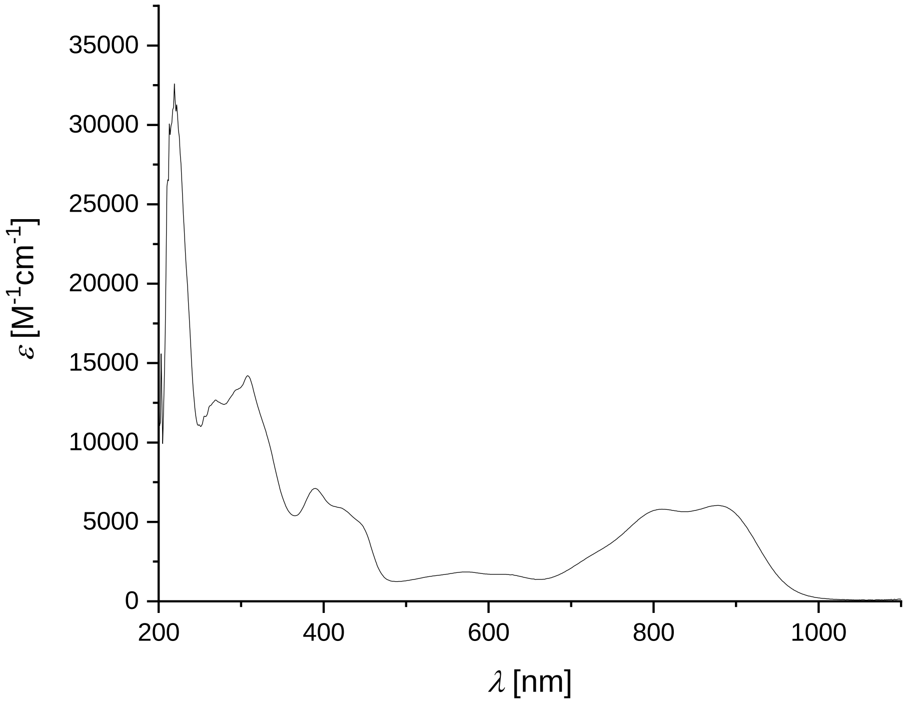

Figure S42. UV-Vis spectrum of **4**, THF, RT.

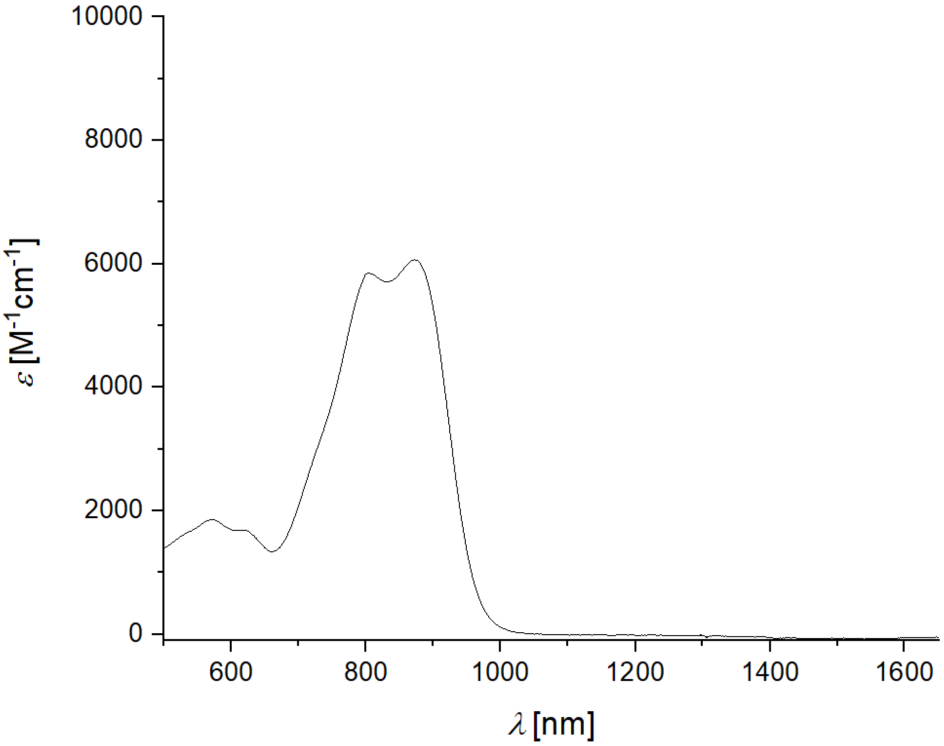

Figure S43. NIR spectrum of **4**, THF, RT.

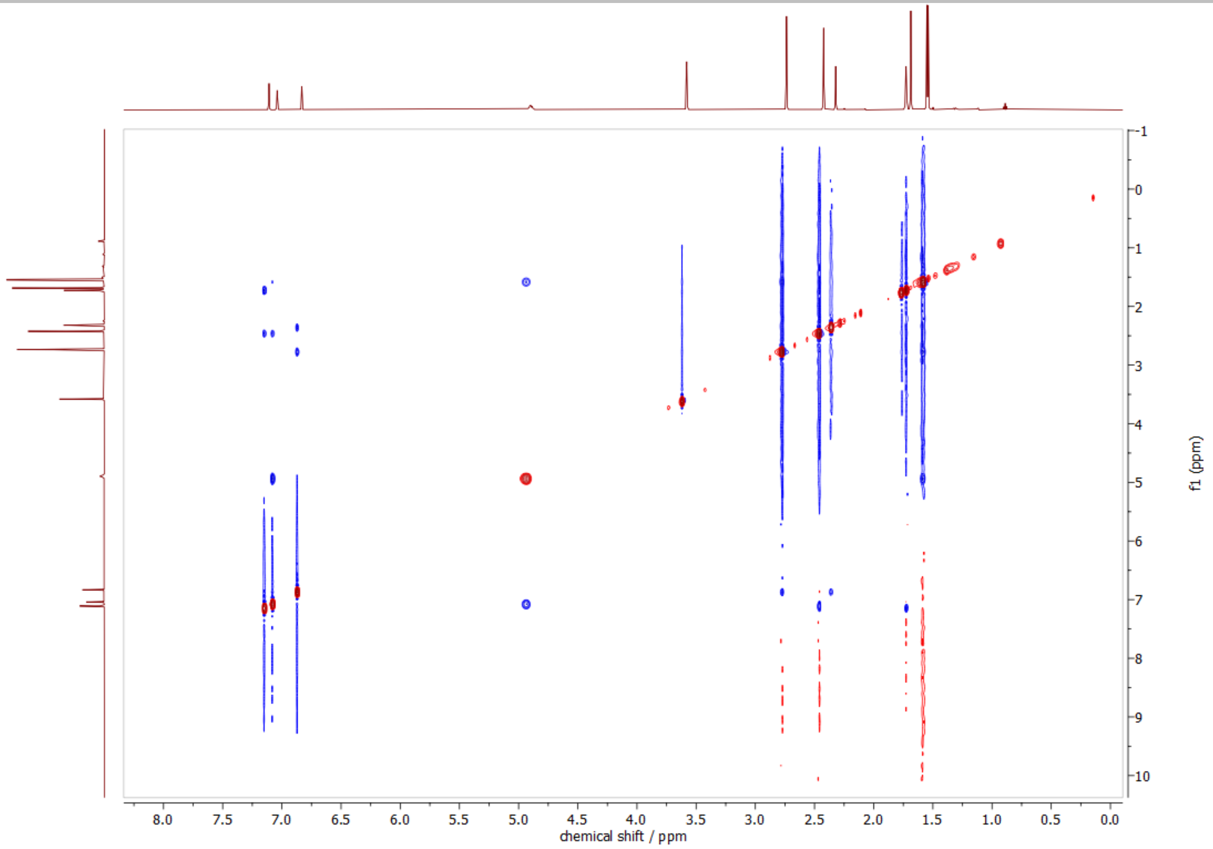

Figure S44. NOESY NMR spectrum of **4**, THF-d_8, RT.

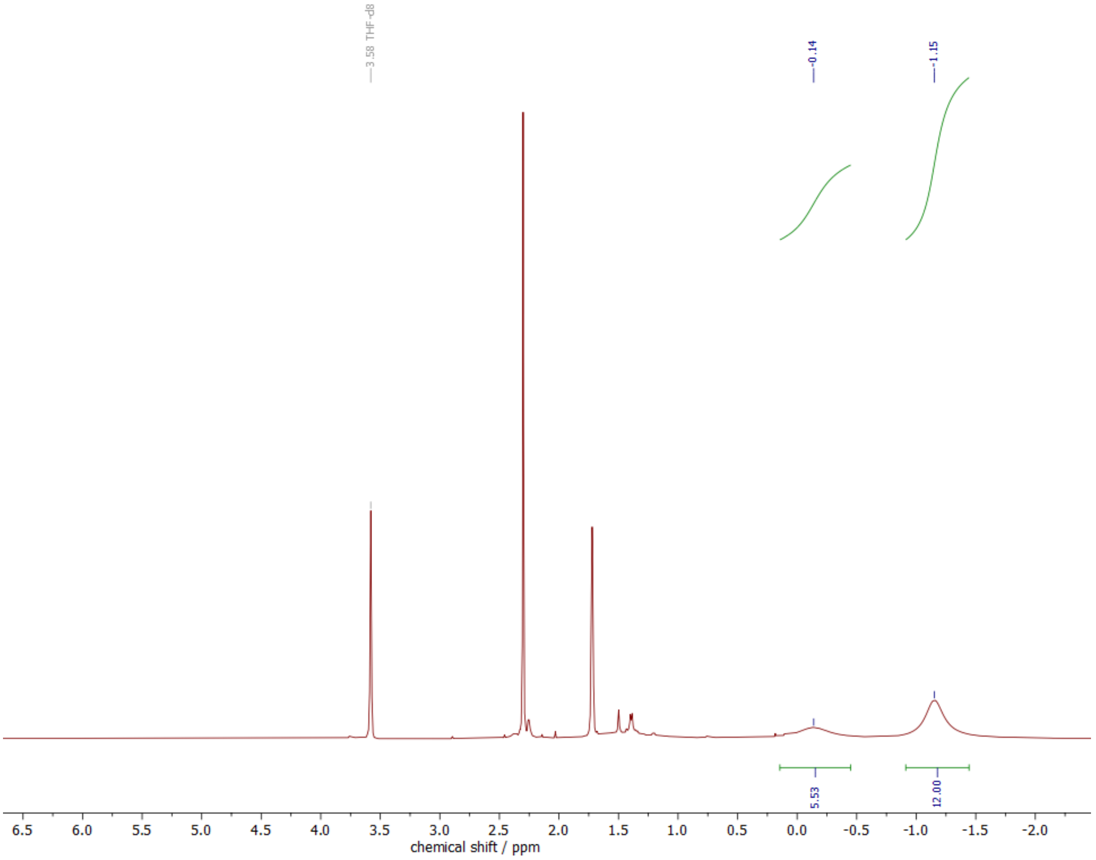

Figure S45. ^{1}H NMR spectrum of **5**, THF-d_8, RT, *residual toluene observed at ca. 2.3 ppm due to crystallization from toluene/pentane layering* (500 MHz).

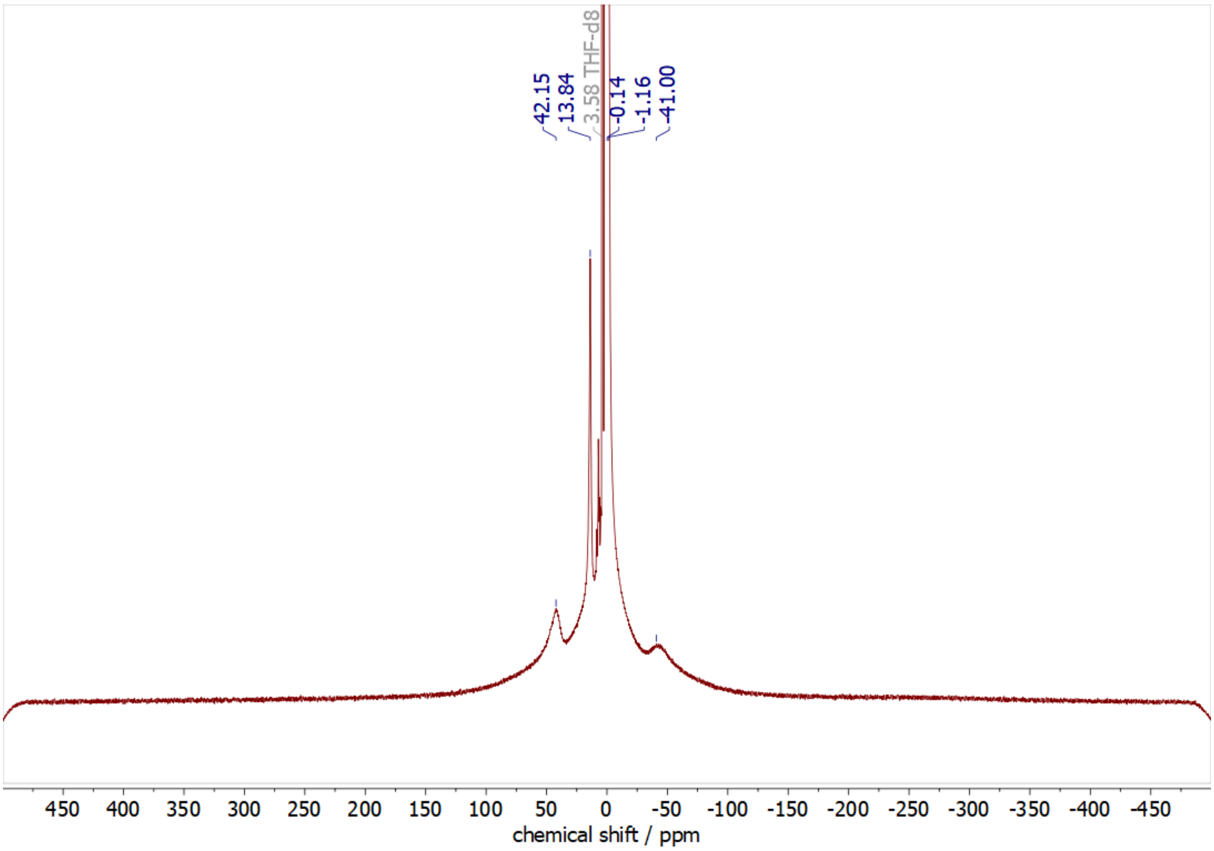

Figure S46. ^{1}H NMR spectrum of **5**, THF-d_8, RT (500 MHz).

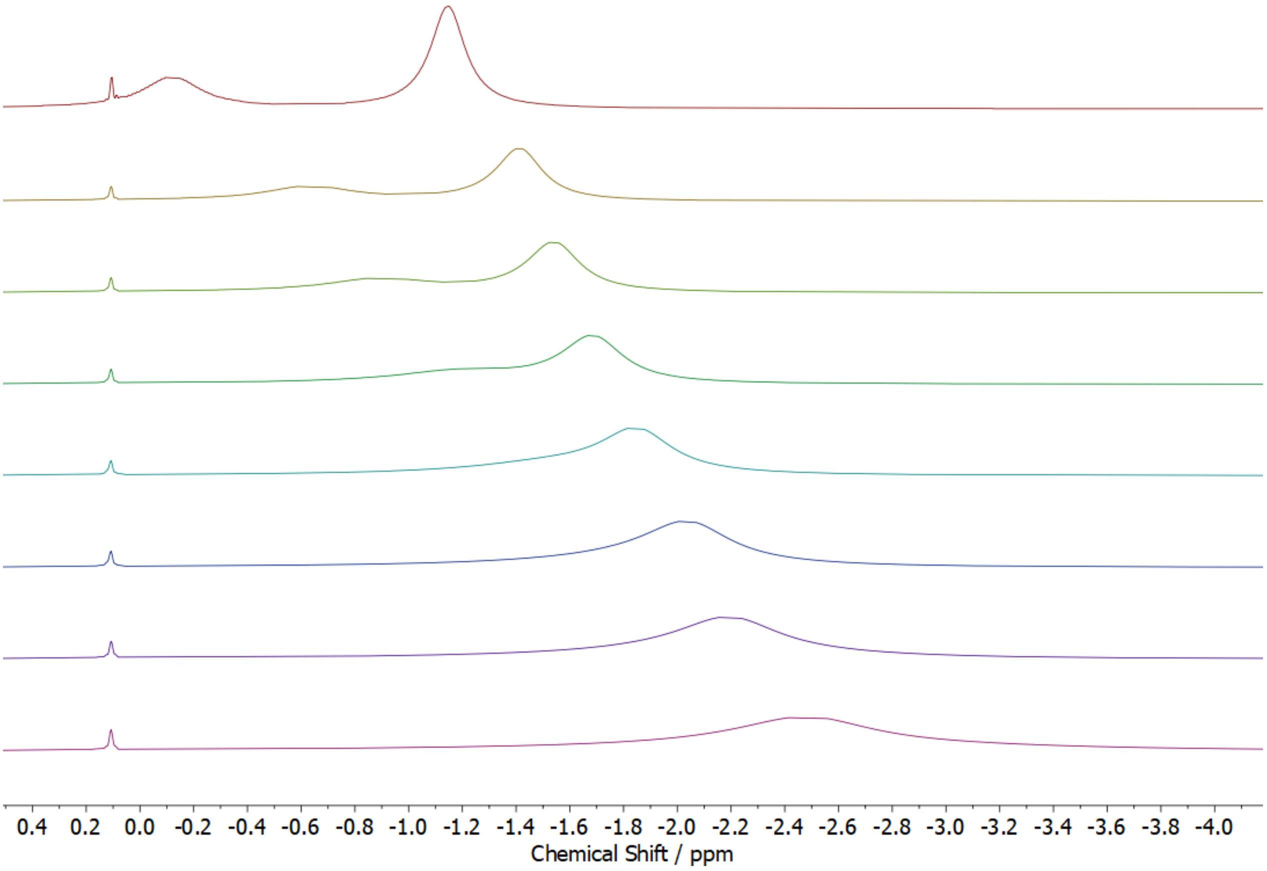

Figure S47. Temperature dependence of **5** (25°C (red), 0°C (yellow) then descending to –60°C in 10°C steps), THF-d_8 (500 MHz).

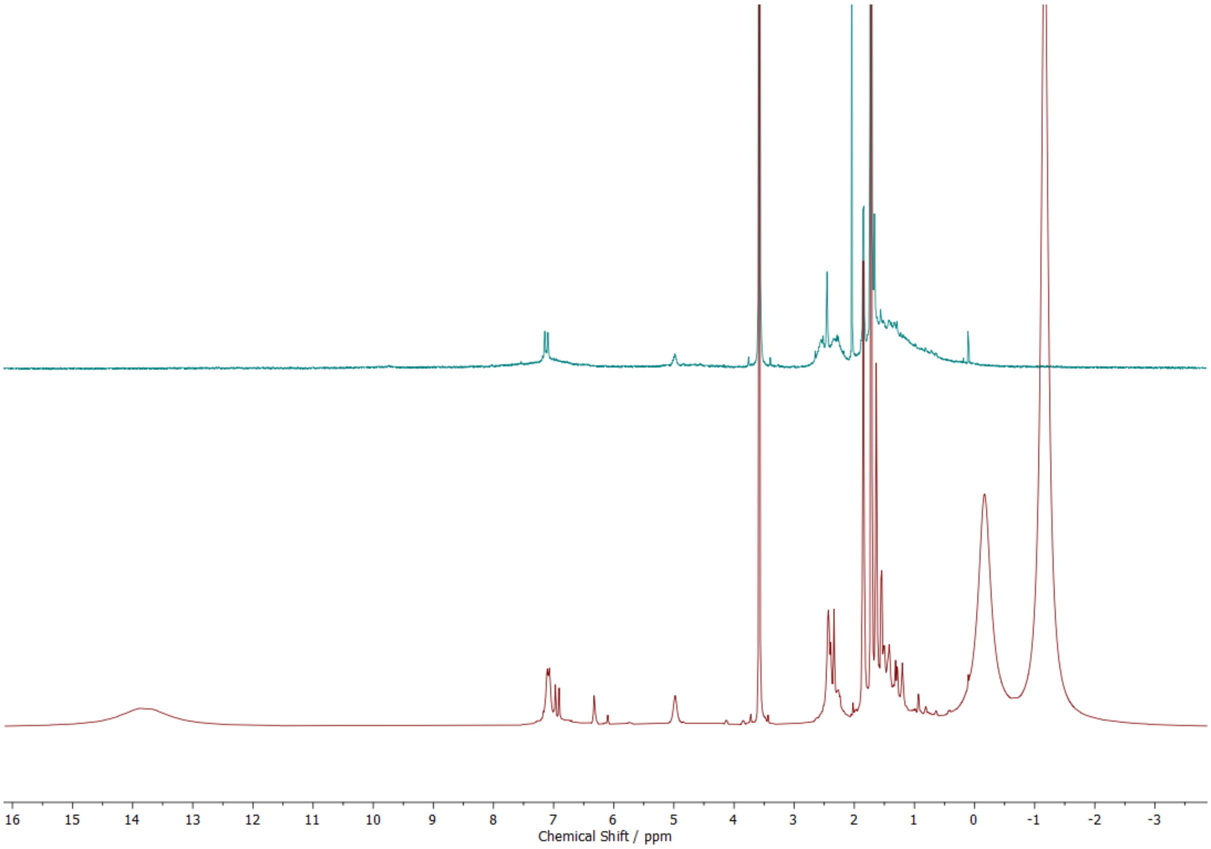

Figure S48. ^1H NMR spectrum of the reaction of **2** with O_2 after reaching room temperature (bottom) and after 12 hours (top) showing the instability of **5** under an O_2 atmosphere (400 MHz).

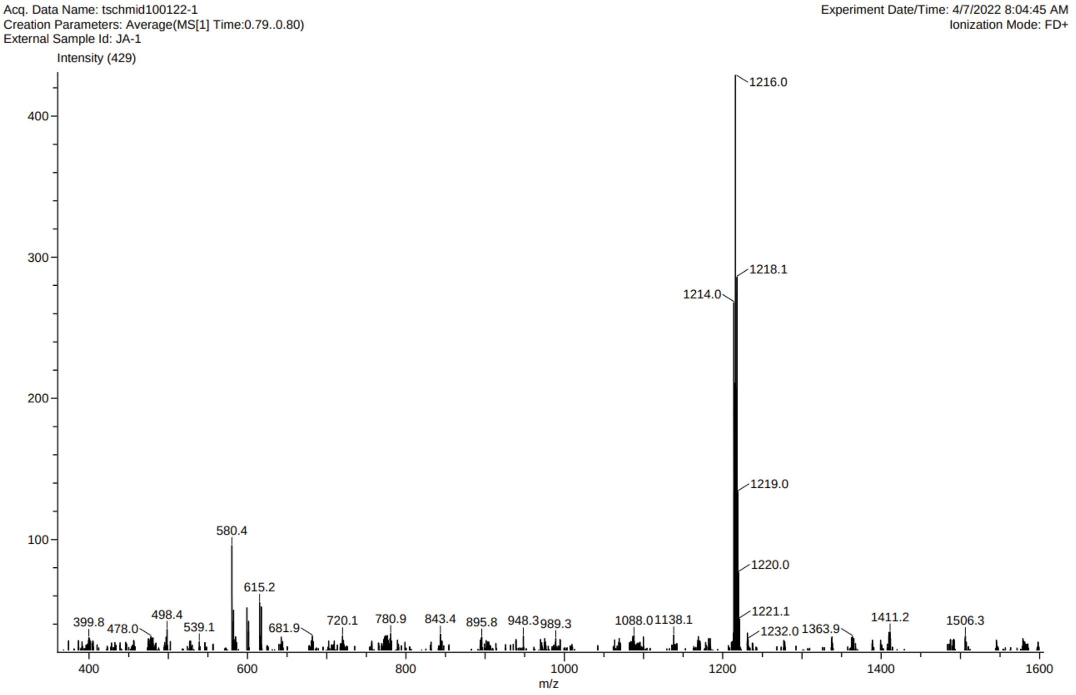

Figure S49. LIFDI-MS spectrum of **5**.

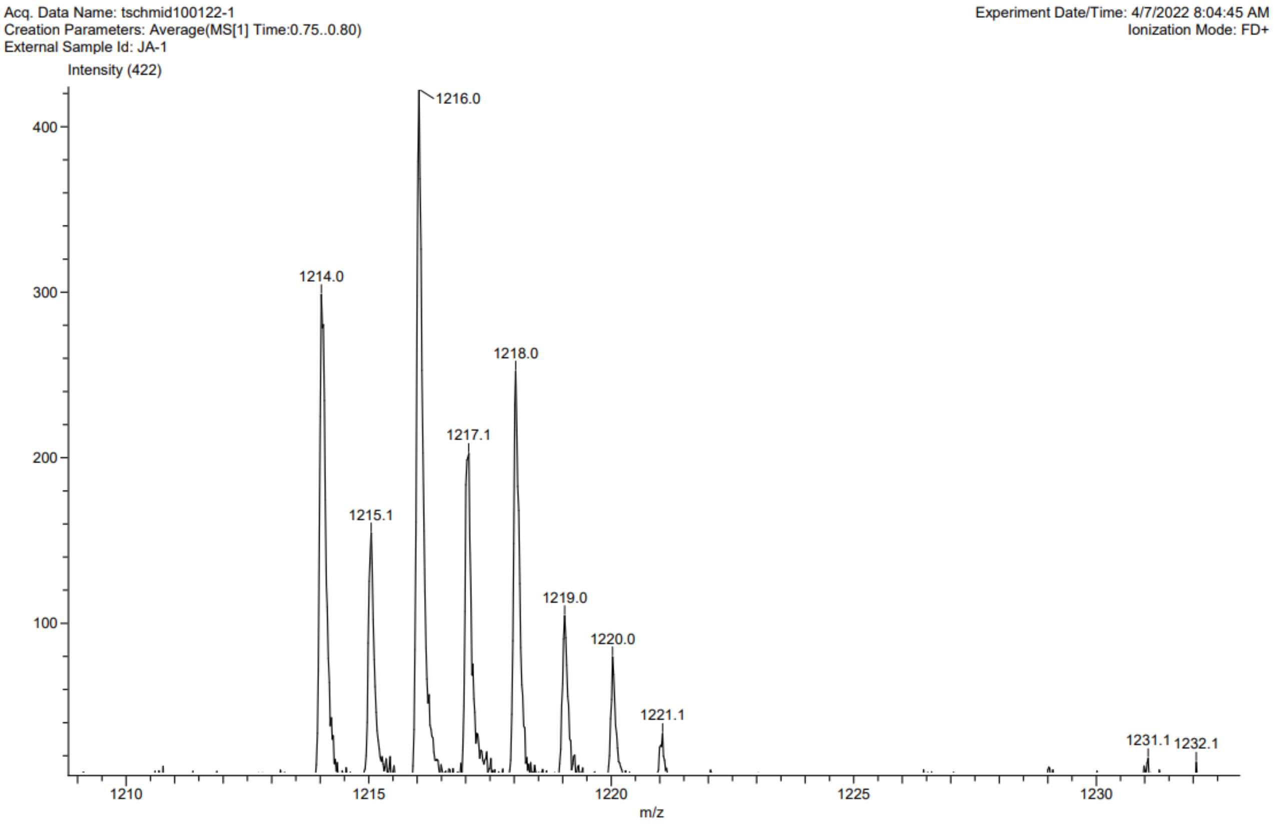

Figure S50. LIFDI-MS spectrum of **5**.

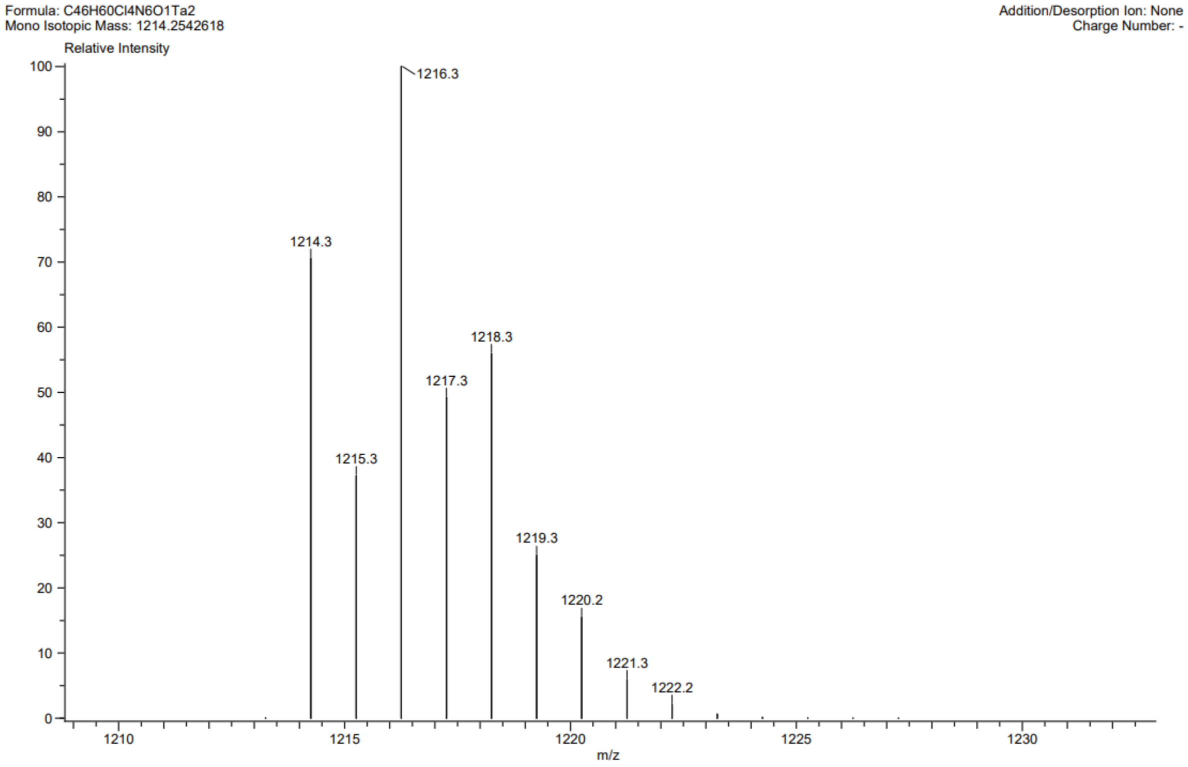

Figure S51. Calculated isotope pattern of **5.**

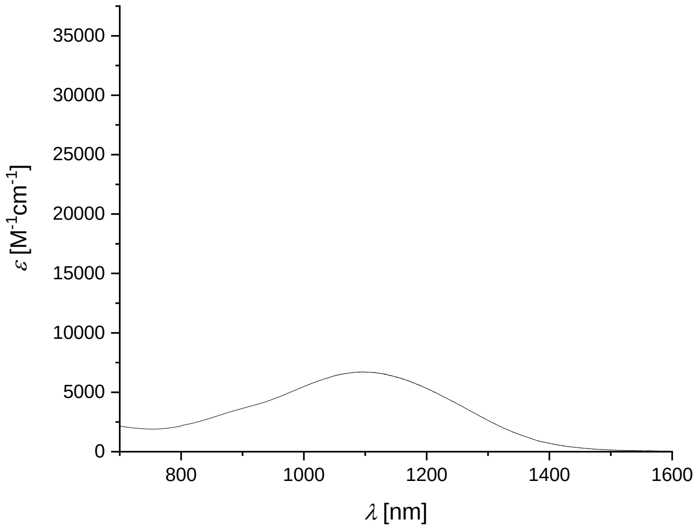

Figure S52. NIR spectrum of **5**, RT.

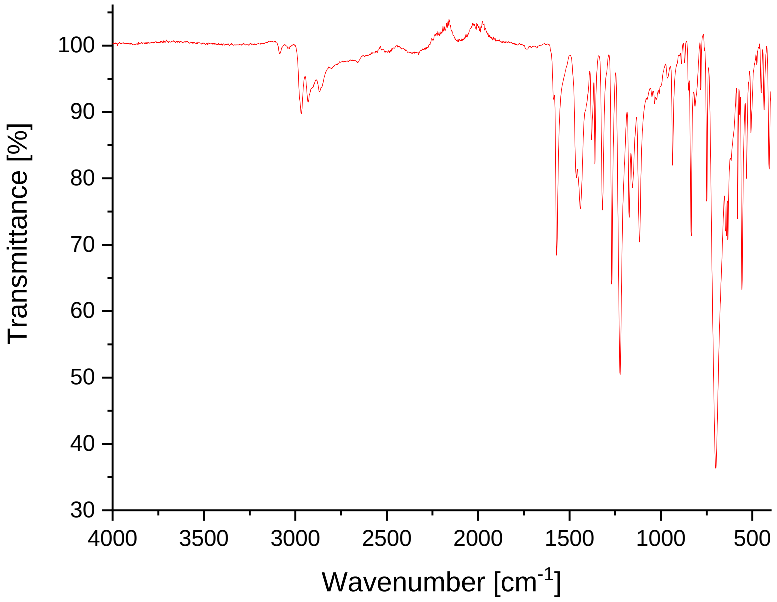

Figure S53. IR spectrum of **5**, RT.

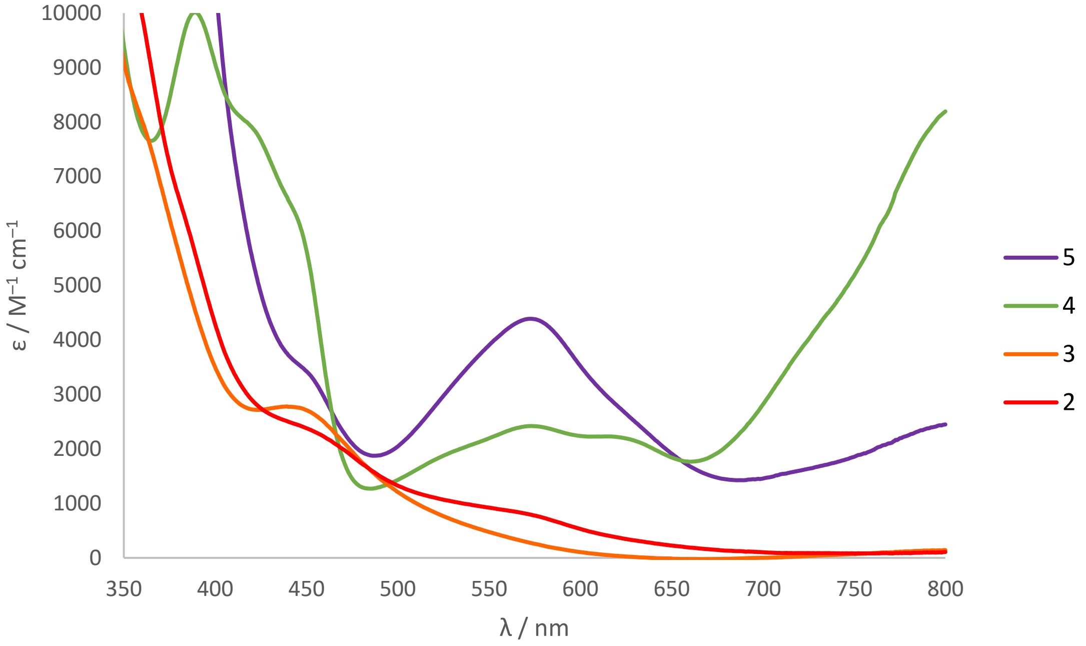

Figure S54. UV/Vis spectra of **2**, **3**, **4** and **5** collected at 298 K in THF.

EPR spectroscopy

The relative half-field intensity as shown in Figure 2 was simulated with *D* = 95 MHz, close to the N-N distance of 8.35 Å[4], however this value only accounts for about 1/3rd of the width of the signal at *g* = 1.965. Direct measure of the sample spin value, an additional means of deconvolution of the S = 1/2 and S = 1 components to what is shown in Figure S55, and measure of hyperfine couplings with pulse EPR were unsuccessful on account of short phase memory times. The *g* = 1.965 features are attributed to field-independent spin interactions, not g-anisotropy, as the width of 50 mT is same value as found in W-band measurements (*not shown*).

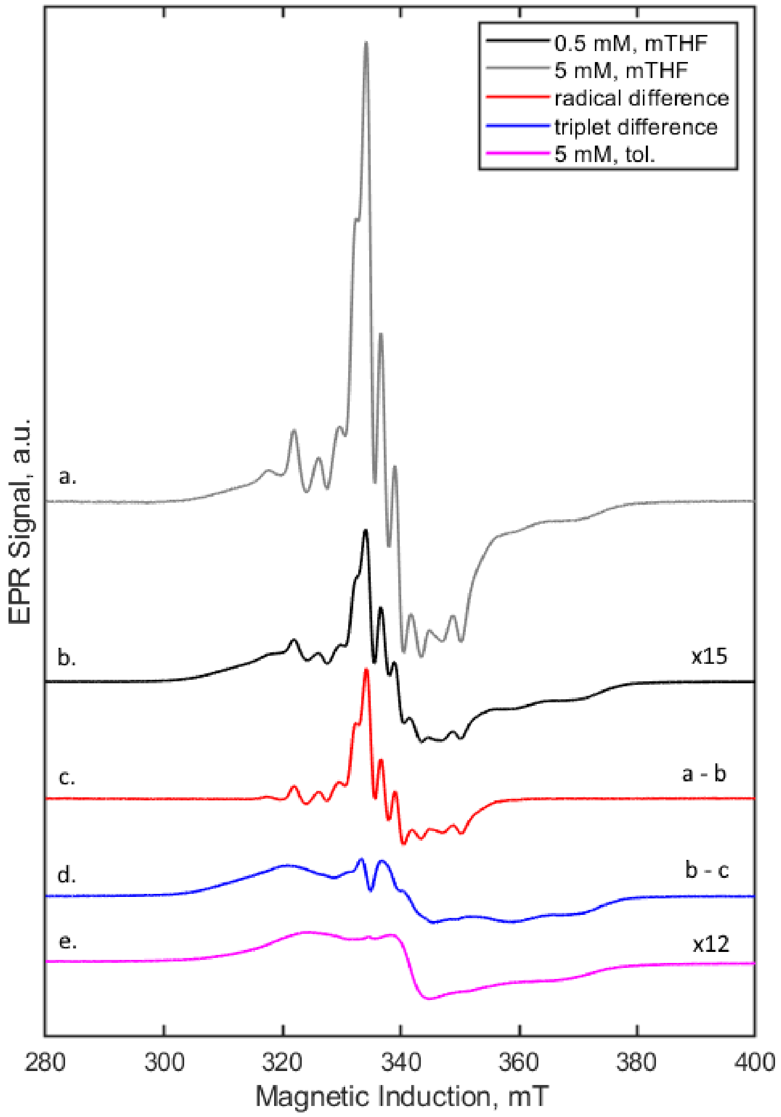

Figure S55. X-band spectral subtraction of the EPR signal in mTHF using two concentrations of **5** at 5 mM and 0.5 mM, in comparison to a sample prepared in toluene.

Crystallographic details

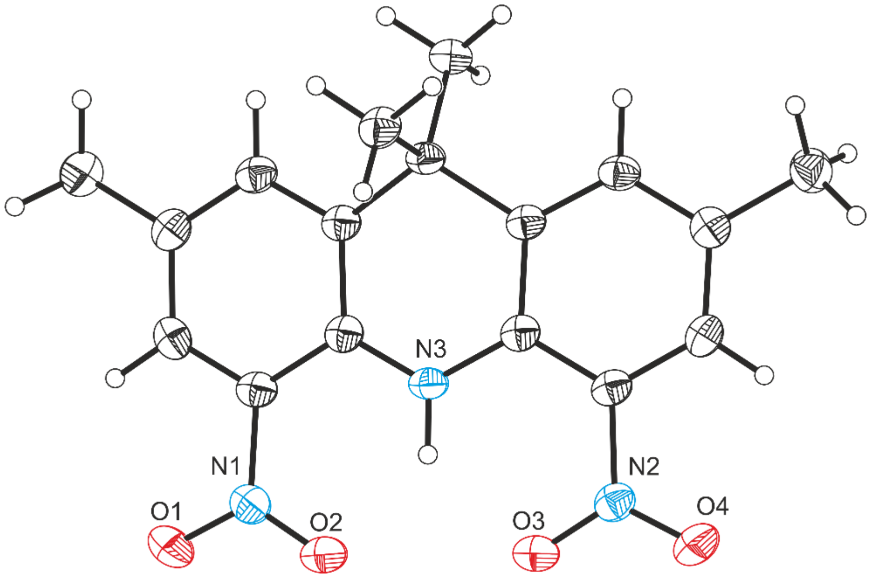

Figure S56. Molecular structure of **IV** in the solid state as determined by single crystal X-ray crystallography. Anisotropic displacement ellipsoids pictured at 50% probability.

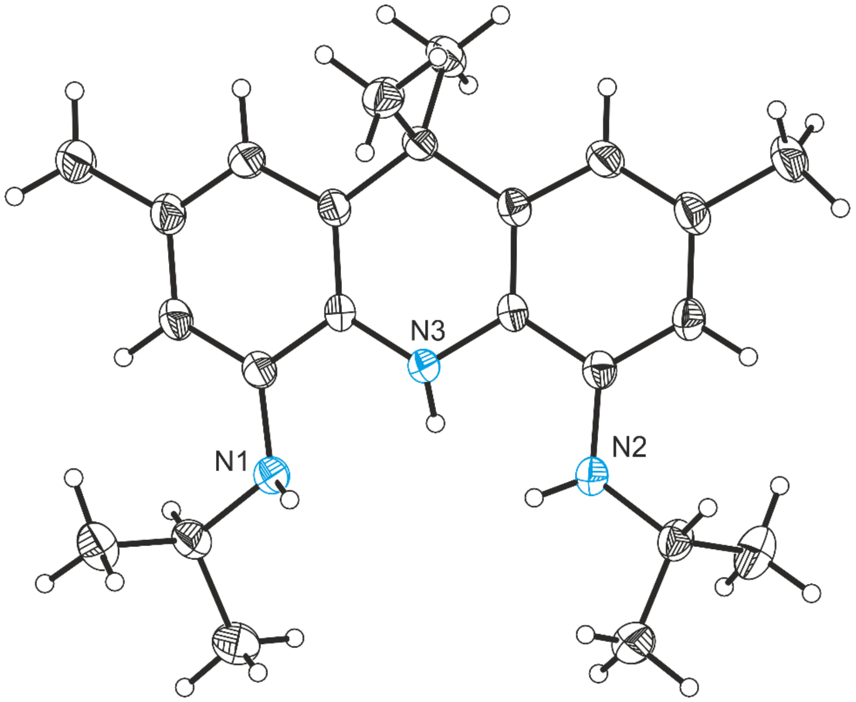

Figure S57. Molecular structure of **1** in the solid state as determined by single crystal X-ray crystallography. Anisotropic displacement ellipsoids pictured at 50% probability.

Table S2. Selected X-ray data collection/refinement parameters for **IV**, **1**, and **2**.

	**IV**	**1**	**2**
Formula	$C_{17}H_{17}N_3O_4$	$C_{23}H_{33}N_3$	$C_{23}H_{30}Cl_2N_3Ta$
CCDC	2191305	2191306	2191307
Fw [g mol^{-1}]	327.33	351.52	600.35
Crystal system	triclinic	orthorhombic	monoclinic
Space group	$P\bar{1}$	$P2_12_12_1$	$P2_1/n$
a (Å)	9.4895(9)	11.6569(2)	10.0652(1)
b (Å)	9.6453(7)	12.5846(2)	14.5301(1)
c (Å)	9.6452(7)	13.8220(3)	16.3868(1)
α (°)	91.177(6)	90	90
β (°)	110.784(7)	90	104.610(1)
γ (°)	112.662(7)	90	90
V (Å^3)	748.88(12)	2027.65(6)	2319.05(3)
Z	2	4	4
Radiation, λ (Å)	Cu Kα, 1.54184	Cu Kα, 1.54184	Cu Kα, 1.54184
Temp (K)	150(2)	150(2)	150(2)
ρ_{calc} (g cm^{-3})	1.452	1.152	1.720
μ (mm^{-1})	0.874	0.515	10.968
Reflections collected	8716	42848	49915
Independent reflections	2634	4240	4837
Parameters	225	256	270
R(int)	0.0360	0.0463	0.0494
R1/wR2,[a] I ≥ 2σI (%)	5.54/15.25	3.33/8.94	2.39/5.85
R1/wR2,[a] all data (%)	6.69/16.21	3.87/9.12	2.84/6.04
GOF	1.060	1.035	1.100

[a] $R1 = [\Sigma||F_o| - |F_c||]/\Sigma|F_o|$; $wR2 = \{[\Sigma w[(F_o)^2 - (F_c)^2]^2]/[\Sigma w(F_o^2)^2]\}^{1/2}$; $w = [\sigma^2(F_o)^2 + (AP)^2 + BP]^{-1}$, where $P = [(F_o)^2 + 2(F_c)^2]/3$ and the A and B values are 0.1065 and 0.07 for **IV**, 0.0587 and 0.20 for **1**, and 0.0281 and 3.18 for **2**.

Table S3. Selected X-ray data collection/refinement parameters for **3**, **4**, **5**·2pent and **5**·3tol.

	**3**	**4**	**5**·2pent	**5**·3tol
Formula	$C_{42}H_{64}Cl_3N_4Ta$	$C_{32}H_{41}Cl_2N_4Ta$	$C_{56}H_{84}Cl_4N_6OTa_2$	$C_{67}H_{84}Cl_4N_6OTa_2$
CCDC	2191308	2191309	21931310	2191311
Fw [g mol^{-1}]	912.27	733.54	1360.99	1493.10
Crystal system	monoclinic	orthorhombic	monoclinic	monoclinic
Space group	$P2_1/n$	*Pbca*	*I*2/*a*	*P*2/*c*
a (Å)	16.7797(2)	13.7777(1)	17.0129(1)	17.9545(2)
b (Å)	16.3818(2)	19.3808(1)	15.3543(1)	10.8983(1)
c (Å)	19.8112(2)	23.9553(1)	23.8668(3)	16.5626(1)
α (°)	90	90	90	90
β (°)	110.426(1)	90	110.719(1)	98.655(1)
γ (°)	90	90	90	90
V (Å^3)	1464.89(8)	6396.61(6)	5831.31(10)	3203.96(5)
Z	4	2	4	2
Radiation, λ (Å)	Cu Kα, 1.54184	Cu Kα, 1.54184	Cu Kα, 1.54184	Cu Kα, 1.54184
Temp (K)	150(2)	150(2)	150(2)	150(2)
ρ_{calc} (g cm^{-3})	1.187	1.523	1.550	1.548
μ (mm^{-1})	5.622	8.076	8.807	8.076
Reflections collected	138198	108871	72415	40377
Independent reflections	10653	6669	6091	6684
Parameters	461	363	365	432
R(int)	0.0826	0.0495	0.0353	0.0487
R1/wR2,[a] I ≥ 2σI (%)	3.54/8.64	2.28/5.33	2.14/5.45	2.35/5.01
R1/wR2,[a] all data (%)	5.03/9.87	3.13/5.77	2.50/5.77	3.49/5.55
GOF	1.033	1.126	1.054	1.069

[a] $R1 = [\Sigma||F_o| - |F_c||]/\Sigma|F_o|$; $wR2 = \{[\Sigma w[(F_o)^2 - (F_c)^2]^2]/[\Sigma w(F_o^2)^2]\}^{1/2}$; $w = [\sigma^2(F_o)^2 + (AP)^2 + BP]^{-1}$, where $P = [(F_o)^2 + 2(F_c)^2]/3$ and the A and B values are 0.0426 and 6.32 for **3**, 0.0216 and 8.07 for **4**, 0.0282 and 16.73 for **5**·2pent, and 0.0204 and 2.02 for **5**·3tol.

Computational details

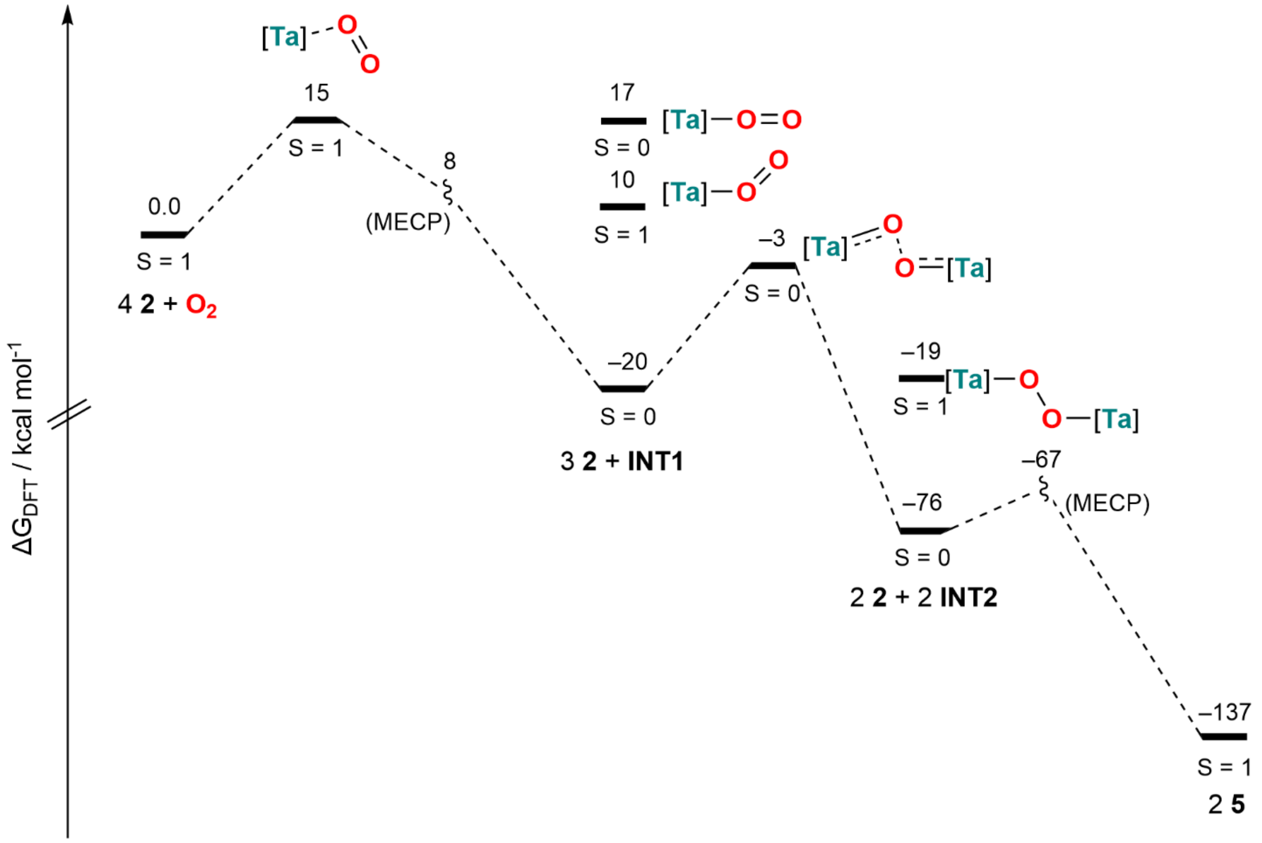

Figure S58. Computed mechanism for the splitting of dioxygen by **2** to produce **5**.

The electronic structures of **2**, **5**, and **INT2** were determined using Kohn-Sham DFT. For **5**, the triplet state and open-shell broken-symmetry singlet state were found to be essentially degenerate (J = 1.6 cm^{-1}), consistent with the low spin-density on tantalum and oxygen.

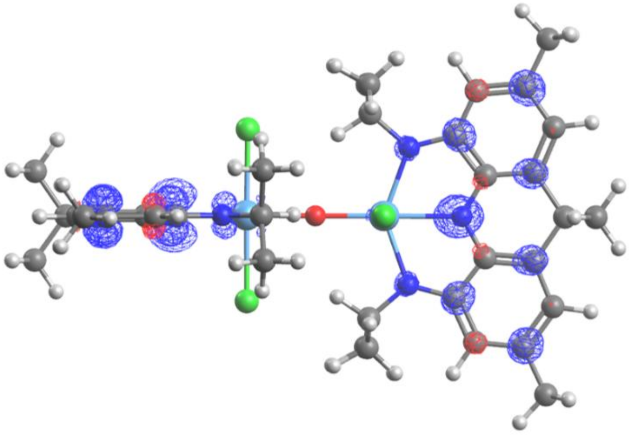

NNN	Ta	O
91.58%	8.07%	0.35%

Figure S59. Spin density plot for **5** (S = 1).

The frontier molecular orbitals of **2**, **5**, and **INT2** show the NNN pincer ligand goes through three different oxidation states over the course of the mechanism.

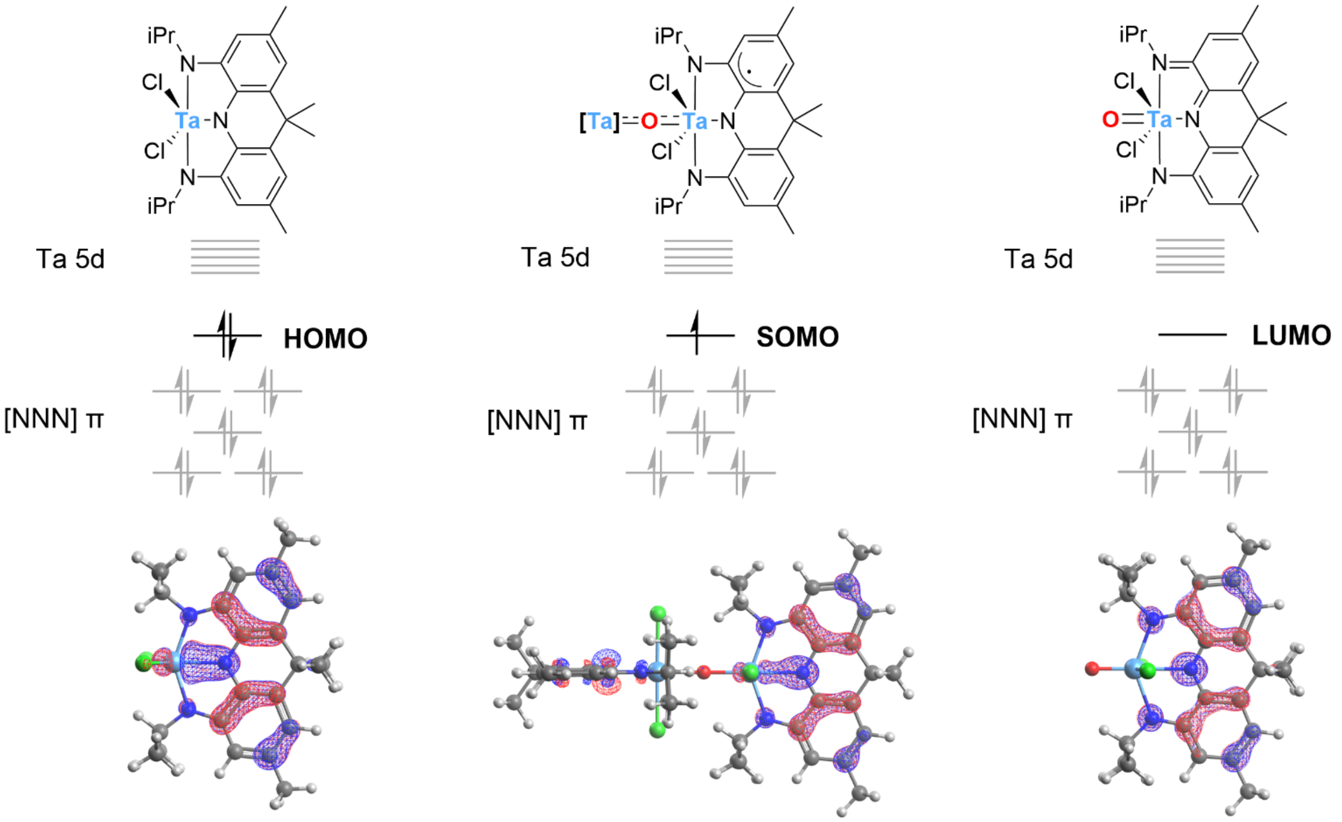

Figure S60. Schematic molecular orbital diagrams for **2**, **5**, and **INT2**, as well as plots of their redox-active ligand

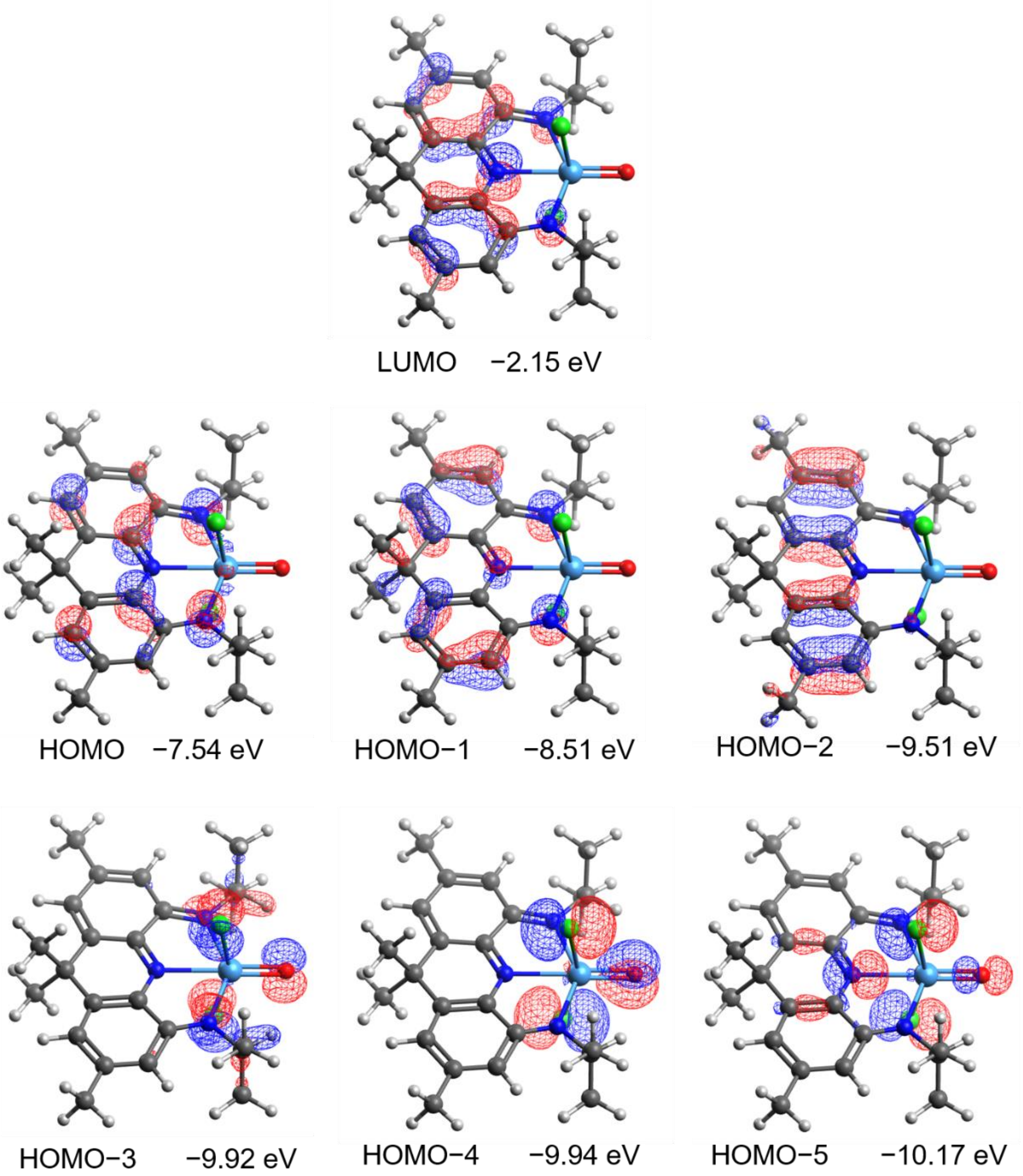

Figure S61. Isosurface plots of the frontier orbitals of **INT2**, showing ligand centered orbitals (LUMO – HOMO−2), orbitals with contributions from the Ta=O π bonds (HOMO−3 and HOMO−4), and the O centered lone pair (HOMO−5).

TDDFT calculations were performed on **2**, **4**, and **5**, giving rise to simulated spectra comparable to experimentally obtained UV-Vis and NIR absorption data.

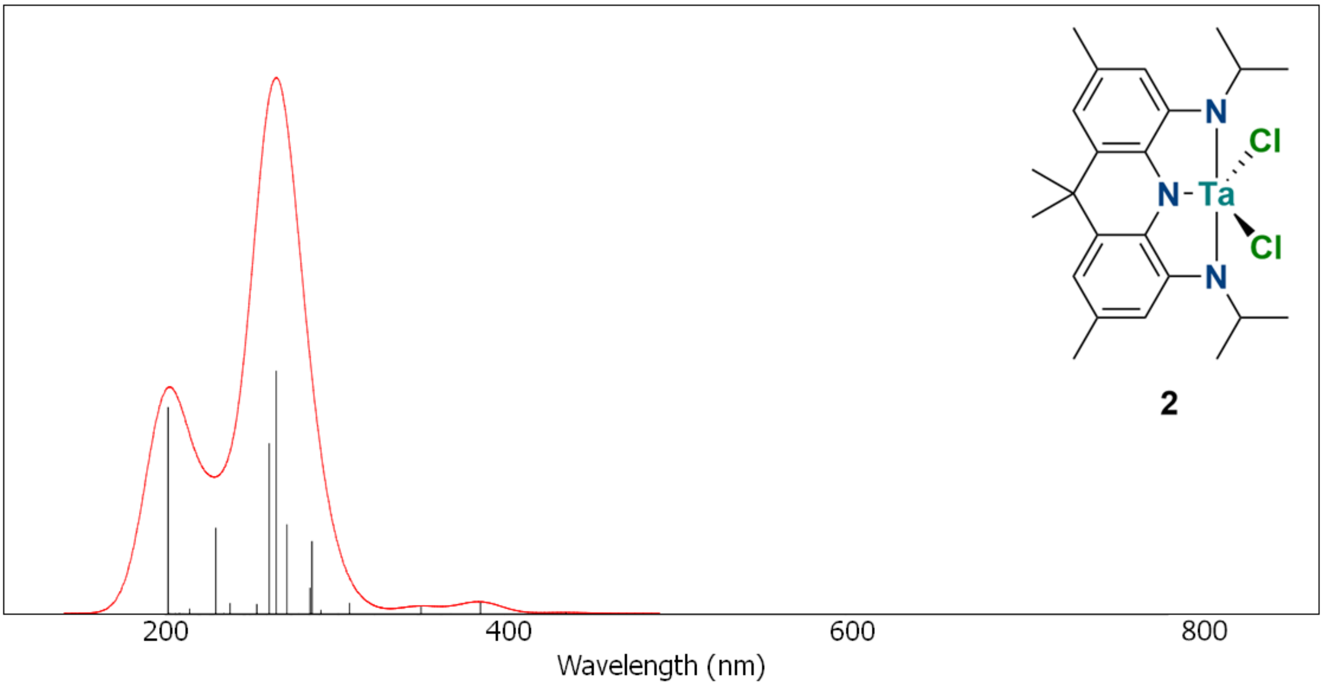

Figure S62. Simulated absorption spectrum for **2**. Lorentzian line shapes drawn at FWHM = 0 nm (black) and FWHM = 50 nm (red).

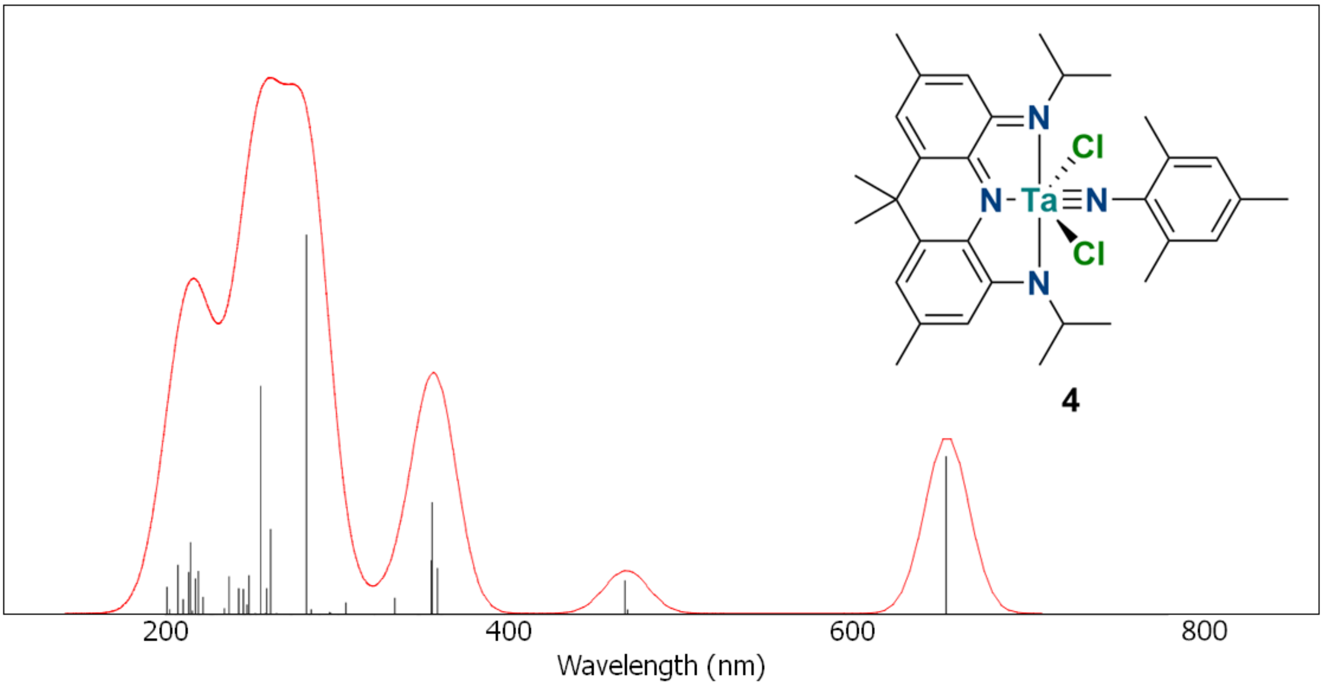

Figure S63. Simulated absorption spectrum for **4**. Lorentzian line shapes drawn at FWHM = 0 nm (black) and FWHM = 50 nm (red).

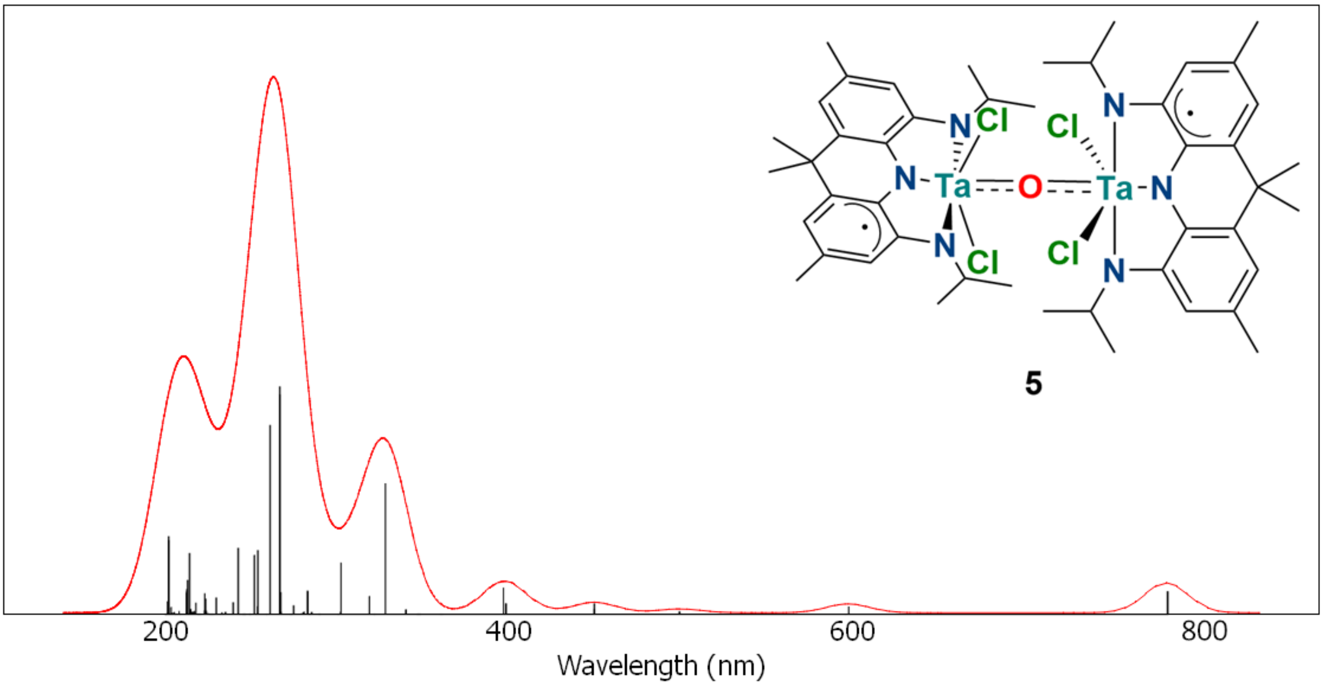

Figure S64. Simulated absorption spectrum for **5**. Lorentzian line shapes drawn at FWHM = 0 nm (black) and FWHM = 50 nm (red).

Table S4. Selected TDDFT calculated transitions for **2**, **4**, and **5**, and their major (> 0.1) donor/acceptor orbital contributions.

	Transition / nm	Donor Orbital	Acceptor Orbital	Weight
**2**	432.0	π(NNN)	d(Ta)	0.96
	382.1	π(NNN)	d(Ta)	0.95
	364.9	π(NNN)	d(Ta)	0.94
**4**	654.4	π(NNN)	π(NNN)	0.92
**5**	782.7	$\pi(NNN)_{\alpha}$	$\pi(NNN)_{A}$	0.47
		$\pi(NNN)_{B}$	$\pi(NNN)_{B}$	0.43
	782.6	$\pi(NNN)_{B}$	$\pi(NNN)_{B}$	0.47
		$\pi(NNN)_{A}$	$\pi(NNN)_{A}$	0.43

References

[1] a) A. Sattler, S. Ruccolo, G. Parkin, *Dalton Trans.* **2011**, *40*, 7777–7782; b) A. Hubbard, T. Okazaki, K. K. Laali, *J. Org. Chem.* **2008**, *73*, 316–319.

[2] a) F. You, J. Zhai, Z.-M. So, X. Shi, *Inorg. Chem.* **2021**, *60*, 1797–1805, b) E. W. Y. Wong, D. J. H. Emslie *Dalton Trans.*, **2015**, *44*, 11601-11612.

[3] H. Liu, N.-D. Wang, D.-M. Du, *Lett. Org. Chem.* **2010**, *7*, 114–120.

[4] S. Eaton, K. More, B. Sawant, G. Eaton. *J. Am. Chem. Soc*. **1983**, *105*, 6560-7.
